# Extended Pharmacokinetics Improve Site-Specific Prodrug Activation Using Radiation

**DOI:** 10.1021/acscentsci.4c00354

**Published:** 2024-06-21

**Authors:** Jeremy M. Quintana, Mikyung Kang, Huiyu Hu, Thomas S. C. Ng, Gregory R. Wojtkiewicz, Ella Scott, Sareh Parangi, Jan Schuemann, Ralph Weissleder, Miles A. Miller

**Affiliations:** † Center for Systems Biology, Massachusetts General Hospital Research Institute, Boston, Massachusetts 02114, United States; ‡ Department of Surgery, Massachusetts General Hospital and Harvard Medical School, Boston, Massachusetts 02114, United States; § Department of Radiation Oncology, Massachusetts General Hospital and Harvard Medical School, Boston, Massachusetts 02114, United States; ∥ Department of Radiology, Massachusetts General Hospital and Harvard Medical School, Boston, Massachusetts 02114, United States; ⊥ Department of Systems Biology, Harvard Medical School, Boston, Massachusetts 02115, United States

## Abstract

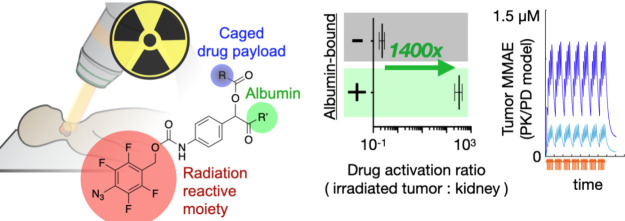

Radiotherapy is commonly used to treat cancer, and localized energy deposited by radiotherapy has the potential to chemically uncage prodrugs; however, it has been challenging to demonstrate prodrug activation that is both sustained *in vivo* and truly localized to tumors without affecting off-target tissues. To address this, we developed a series of novel phenyl-azide-caged, radiation-activated chemotherapy drug-conjugates alongside a computational framework for understanding corresponding pharmacokinetic and pharmacodynamic (PK/PD) behaviors. We especially focused on an albumin-bound prodrug of monomethyl auristatin E (MMAE) and found it blocked tumor growth in mice, delivered a 130-fold greater amount of activated drug to irradiated tumor versus unirradiated tissue, was 7.5-fold more efficient than a non albumin-bound prodrug, and showed no appreciable toxicity compared to free or cathepsin-activatable drugs. These data guided computational modeling of drug action, which indicated that extended pharmacokinetics can improve localized and cumulative drug activation, especially for payloads with low vascular permeability and diffusivity and particularly in patients receiving daily treatments of conventional radiotherapy for weeks. This work thus offers a quantitative PK/PD framework and proof-of-principle experimental demonstration of how extending prodrug circulation can improve its localized activity *in vivo*.

## Abstract 2

Data-driven computational modeling interprets how radiation can locally uncage prodrugs in tumors and captures the experimental *in vivo* performance of a novel long-circulating albumin-bound prodrug.

## Introduction

Roughly half of all cancer patients receive radiation therapy (RT). To balance efficacy with safety, most RT is delivered in dose fractions across consecutive days and weeks.^1−3^ Off-target exposure is minimized by using highly conformal RT techniques, such as volumetric modulated arc therapy (VMAT) X-rays, but such high-energy photons unavoidably expose tissues around their target. Proton therapy is an alternative RT modality that deposits a Bragg dose peak within a limited tissue depth.^4^ The beneficial dose deposition pattern of proton therapy reduces off-target irradiation and has been used in >170 000 patients worldwide.^5,6^

RT is frequently combined with chemotherapies that exhibit dose-limiting toxicities owing to their nonspecific action throughout the body.^7,8^ Current chemoradiotherapy regimens fail to cure many advanced cancers, and patients who achieve durable responses can be left with life-altering side effects. To minimize such off-target toxicities, numerous strategies have been investigated to enhance drug action and/or radiosensitization locally in irradiated tumor tissues while sparing nontumor sites. Prodrugs have been developed to activate in response to endogenous physicochemical cues such as hypoxia in the radioresistant tumor microenvironment.^9,10^ Transition metal nanoparticles and other materials have been designed to absorb radiation and enhance the local production of reactive oxygen species. These approaches have advanced to clinical trials in several cases^11^ but remain hampered by off-target effects and reliance on features that can vary from patient to patient, including tumor oxygenation and the ability of nanoparticles to evenly deposit in tumor tissue.

To overcome these limitations, a class of radiation-activated prodrugs has been recently reported to uncage selectively in response to RT-generated reactive species including free radicals generated during H_2_O radiolysis. Quaternary ammonium groups, *N*-oxides, phenyl azides, and dimethoxy benzyl alcohols have been recently identified as moieties that react with radiolysis products to uncage upon RT exposure.^12−17^ In principle, such prodrugs exhibit low native toxicity but become effective anticancer agents upon irradiation. They have the advantage of not relying on endogenous and heterogeneous physicochemical properties or biochemical processes of the tumor microenvironment; they do not rely on inorganic materials that may unevenly distribute or show toxicity, and they potentially apply to a broad range of drug classes including cytotoxic chemotherapies, targeted therapies, protein degraders, and others that may exhibit superior therapeutic indices and less cross-resistance compared to mere amplification of reactive oxygen species. In practice, however, these strategies are still affected by tissue oxygenation and off-target metabolism, and localized uncaging efficiencies remain unclear *in vivo*.^12,18,19^

Despite progress, it remains challenging to demonstrate radiation-activated prodrug approaches that are sustained, responsive to both proton and X-ray RT, and truly localized to tumor tissue. *In vivo* pharmacokinetic/pharmacodynamic (PK/PD) design principles for radiation-activated prodrugs are needed to translate new chemical advances into effective therapies. The PK/PD processes governing the dose and localization of a drug in the target tissue are complex. Even if RT uncages a prodrug with perfect efficiency and selectivity, such an approach could be useless if the uncaged drug freely redistributes in the body once activated or if it never reaches the tumor in the first place. Quantitative and computational frameworks have elucidated PK/PD mechanisms of traditional small molecules and drug-conjugates,^20−22^ but they remain underutilized for studying radiation-activatable therapeutics.

In this work, we hypothesized that the *in vivo* efficiency of radiation-activated prodrugs could be vastly improved through the design of a long-circulating construct that steadily accumulates in tumor tissue and releases chemotherapy payloads following clinically relevant doses or serial dose fractions of RT. Numerous strategies can extend the circulating pharmacokinetics of drug payloads and improve their delivery and retention at tumor sites,^23−25^ including through payload incorporation into long-circulating PEGylated nanoparticles,^26,27^ tumor-targeted antibodies,^28−32^ and serum albumin.^33−38^ We show how our modular chemical design for radiation-activated prodrugs can apply to each of these strategies and focus especially on serum albumin for its extended blood half-life (3 weeks in humans), relatively low molecular weight and high diffusivity compared to antibodies and nanoparticles, its extensive use in developing both molecularly targeted and passively accumulating therapies for cancer, and its ability to be taken up into tumors via oncogene-driven macropinocytosis and the “enhanced permeability and retention” (EPR) effect.^39,40^ Thus, we present the design, synthesis, computational PK/PD modeling, and *in vivo* evaluation of the **R**adioactivated **A**lbumin-**B**ound **i**nducible **T**herapeutic (RABiT) platform and show how it efficiently accumulates in tumor tissue, selectively releases drug payload, and synergistically blocks tumor growth in mice.

## Results

### RABiT Chemical Design, Synthesis, and Characterization

**Figure 1:**
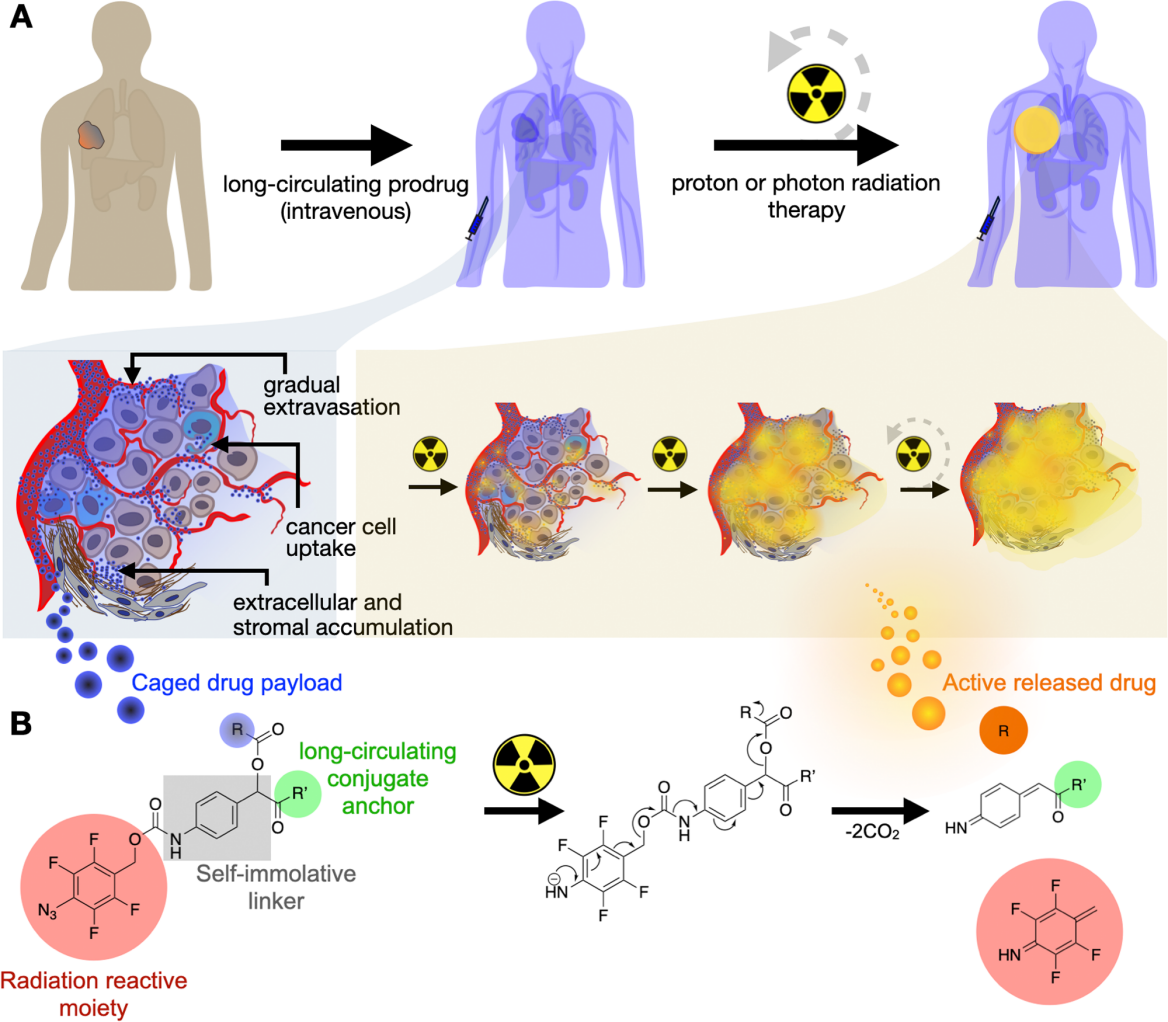
Concept and chemical strategy for local activation of long-circulating drug-conjugate using radiotherapy. (A) Caged drug payload, for instance, covalently anchored to long-circulating serum albumin or PEGylated nanoparticle, is systemically administered and gradually accumulates in tumors. Payload is released in tissue following single radiation treatments with external beams of high-energy protons or photons (X-rays), and extended pharmacokinetics align with dose fractionation regimens that are used clinically, whereby multiple smaller doses of radiation are given across days and weeks to maximize the therapeutic window. (B) Chemical design. Radiation-mediated reduction of a phenyl azide moiety triggers the release of a drug payload from its long-circulating anchor, such as serum albumin.

Hydrated electrons (e^–^_aq_) and hydrogen radicals (^•^H) are the major reducing species generated during H_2_O radiolysis and are produced with oxidizing species including hydroxyl radicals (^•^OH). Because ^•^OH-mediated uncaging is negatively affected by dissolved O_2_ during RT,^16^ we hypothesized that prodrug reduction could offer more robust activation *in vivo*. Therefore, we built on prior designs to synthesize a series of drug-conjugates based on radiation-sensitive phenyl azide caging.^41,42^ In principle, radiation-activated azide reduction triggers self-immolation of a 4-aminobenzyl alcohol linker to release an active cytotoxic payload from the albumin conjugate (Figure 1).

**Figure 2:**
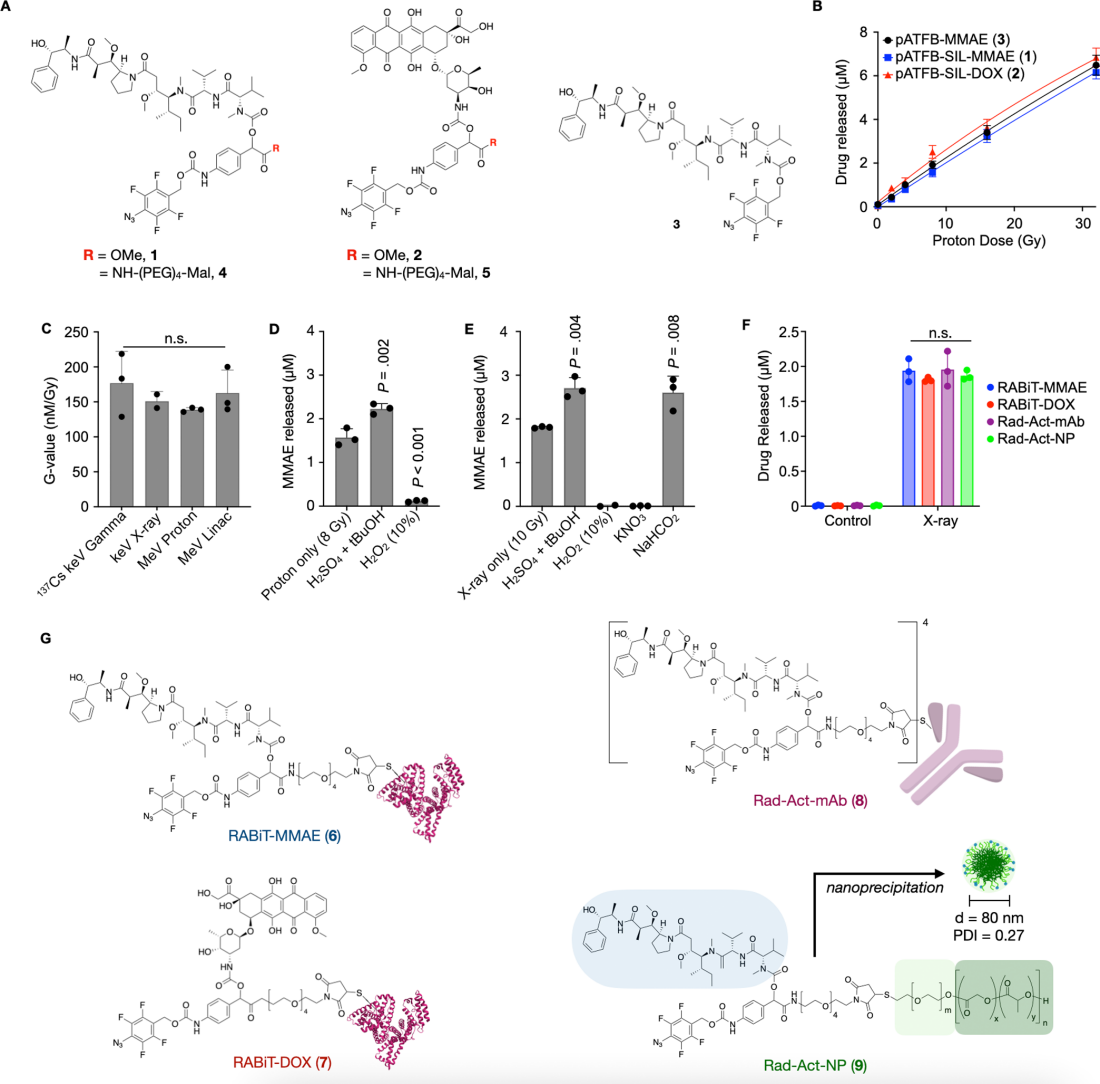
*In vitro* prodrug activation. (A) Structures of drug-conjugate precursors **1** and **2** and nonconjugating control **3**. (B) Monomethyl auristatin E (MMAE) or doxorubicin (DOX) released from 10 μM prodrugs **1**, **2**, and **3** after proton irradiation. (C–E) MMAE released from prodrug **1** under various irradiation methods at 10 Gy (C), with redox conditions after 8 Gy proton irradiation (D), and with redox conditions after 10 Gy X-ray irradiation (E). (F, G) 10 Gy radiation-mediated activation of prodrugs **4** and **5** conjugated to a series of drug delivery vehicles: serum albumin to form RABiT-MMAE **6** and RABiT-DOX **7** (**R**adioactivated **A**lbumin-**B**ound **i**nducible **T**herapeutic; HSA PDB 1AO6), a model tumor-targeted antibody **8**, and a model PEGylated polymer nanoparticle **9**. Data are *n* = 3, means ± SEM.

*Para*-azido-2,3,5,6-tetrafluorobenzyl alcohol (pATFB) precursor was synthesized as prior,^41,43^ conjugated via carbamate to a self-immolative 4-aminobenzyl alcohol linker, and reacted with either the microtubule destabilizing monomethyl auristatin E (MMAE) or doxorubicin (DOX) as model chemotherapy payloads, providing prodrugs **1** and **2**, respectively (Figure 2A and Scheme S1). We synthesized caged MMAE lacking covalent albumin-binding capacity as a control (**3**) (Figure 2A and Scheme S1). Ester hydrolysis of **1** and **2** followed by amide coupling of a maleimide polyethylene glycol linker (Mal-PEG_4_-NH_2_) yielded the final prodrugs **4** and **5**.

We tested whether prodrugs could be activated by X-ray and clinical proton beam sources; 10 μM aqueous solutions were irradiated, and active drug release was monitored by LC/MS. Release was roughly linear (Figures 2B and S1A) and similar between protons and photons via multiple radiation methods, including clinical proton and linear accelerator sources (Figure 2C). H_2_O radiolysis generates e^–^_aq_ (270 nM Gy^–1^) and ^•^H (62 nM Gy^–1^) reducing species,^44^ and we hypothesized that prodrug release was driven by phenyl azide reduction. To test this, we repeated the experiments under conditions that promote or quench hydrated electron availability.^45^ Irradiation in the presence of reducing agents such as sodium formate or 0.01 M sulfuric acid in 1 M *tert*-butanol (a known scavenger of hydroxyl radicals) increased radioactivation by ∼10%, while oxidizing agents such as potassium nitrate and hydrogen peroxide eliminated drug release (Figure 2D,E). Thus, prodrugs exhibited *G*-values of 1.4–1.6 molecules/100 eV (∼170 nM Gy^–1^), consistent with radiolysis-mediated reduction via e^–^_aq_ as a primary reductant. Nonirradiated prodrugs showed stability for weeks at 37 °C (Figure S1B). Radiation-mediated release occurred in fetal bovine serum (FBS), cell culture growth media (Figure S1C), and from pH 3 to 8 (Figure S2). Prodrug activation therefore depends on radiation and occurs under physiologically relevant conditions.

In principle, the maleimide groups of **4** and **5** support prodrug conjugation to diverse drug delivery vehicles, and we examined whether radiation-triggered drug release could equally occur when anchoring to various model vehicles: serum albumin, a therapeutic antibody, or a polymer micelle nanoparticle. We synthesized each of these drug-conjugates by reacting prodrug **4** or **5** by Michael addition with Cys-34 on albumin (**6**, RABiT-MMAE; **7**, RABiT-DOX), cysteines on the therapeutic antibody trastuzumab (**8**, Rad-Act-mAb), or free thiol on poly(lactic-*co*-glycolic acid)-*b*-poly(ethylene glycol)-thiol, PLGA-PEG-SH, which was formulated into nanoparticles via nanoprecipitation and self-assembly into polymer micelles (**9**, Rad-Act-NP). Syntheses followed published maleimide bioconjugation reactions, nanoprecipitation formulations, and purification by molecular weight cutoff filter centrifugation.^35,46^ After characterization of materials for drug loading (Figure S3), we confirmed they released their MMAE payloads with comparable efficiencies (Figure 2F,G). The modular chemical design strategy thus shows radiation-responsiveness when applied to distinct drug delivery vehicles, sources of radiation (photon versus proton), and drug payloads.

### RABiT Synergistically Combines with RT to Block Cell Growth and Clonogenicity

**Figure 3:**
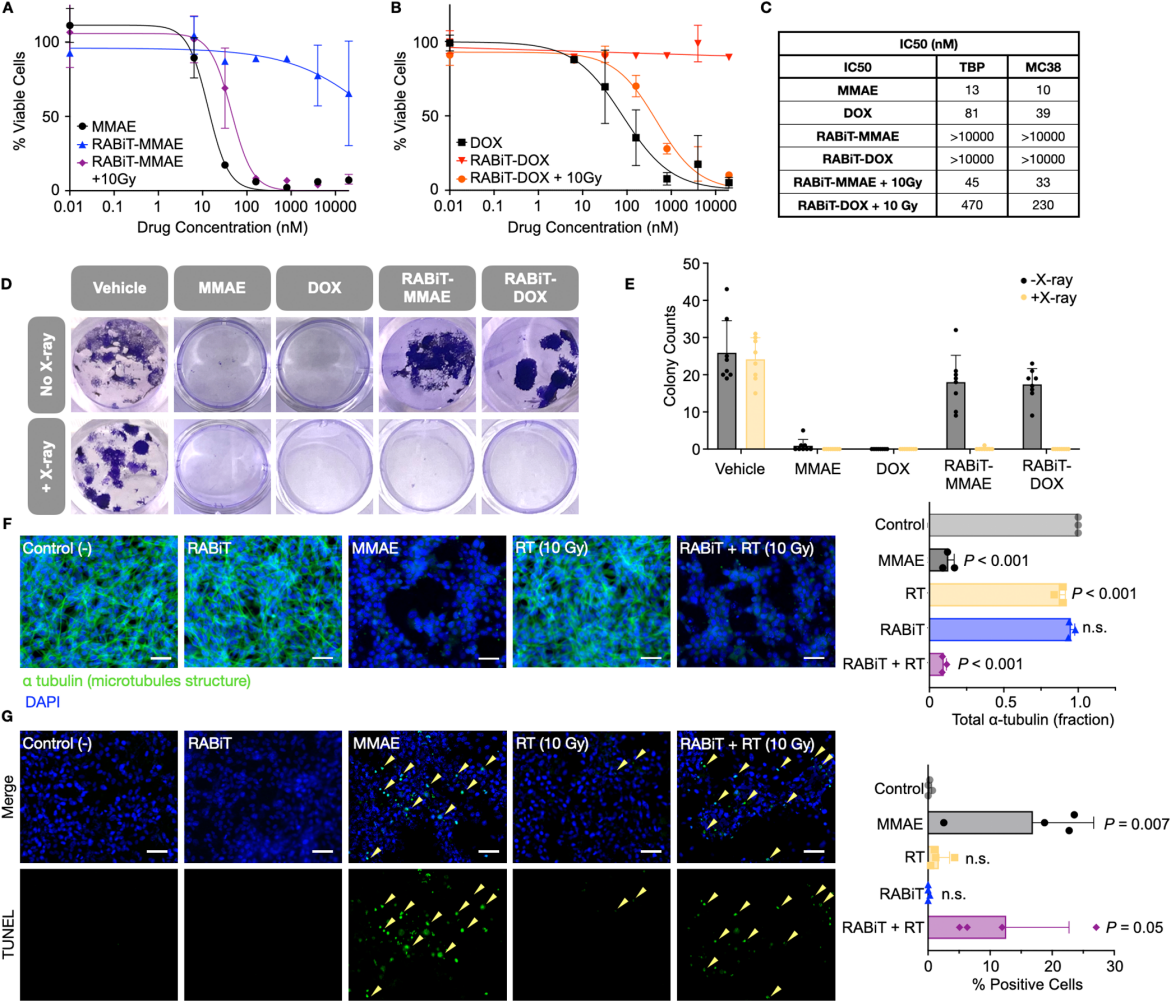
*In vitro* cytotoxic and clonogenic effects. (A, B) Effect of nonirradiated or 10 Gy irradiated RABiT-MMAE (A) or RABiT-DOX (B) on proliferation/cytotoxicity of TBP3743 cancer cells, measured at 72 h by resazurin. (C) Fifty percent proliferation/cytotoxicity inhibition (IC_50_) in TBP3743 and MC38 cancer cells. (D, E) Representative images of TBP3743 colony formation (D) and quantification (E). Data are means ± SEM. (F) Representative images (left) and quantification (right) of microtubule structure immunofluorescence 24 h after treatment in TBP3743 cells. Scale bar = 50 μm. (G) Representative images (left) and quantification (right) of apoptosis by TUNEL staining. Data are means ± SEM. Scale bar = 100 μm.

We evaluated the biological effects of prodrugs using TBP3743 cells derived from a mouse model of anaplastic thyroid cancer (ATC). ATC is an aggressive and deadly rare disease that often shows resistance to conventional chemoradiotherapy.^47^ RABiT-MMAE prodrug irradiation elicited a ∼600-fold increase in drug toxicity as measured by a 72 h cell proliferation assay in ATC cells (Figure 3A), and similar results were seen in another cancer cell line (MC38 colorectal cancer cells, Figures 3B,C and S4A,B) and by using another model drug payload, doxorubicin (RABiT-DOX). Similar patterns of cytotoxic response were observed with the nanoparticle (Rad-Act-NP) and model antibody (Rad-Act-mAb, evaluated only for antigen-independent response) designs, showing how prodrug caging blocks cytotoxicity for multiple days under physiologically relevant conditions and restores cytotoxicity following RT-mediated payload release (Figure S4C,D). A clonogenic assay on ATC cells tested the combined effect of RT and RABiT, revealing their combination blocked colony formation and yielding a statistically synergistic effect (Figure 3D,E; two-way ANOVA interaction term *P* < 0.001). RABiT was therefore effective in cells and elicited a synergistic biological effect *in vitro*.

We next examined the mechanism of synergistic chemoradiotherapy using RABiT in the cells. In principle, RABiT payloads are caged and are unable to engage with their molecular target without irradiation. Focusing on RABiT-MMAE, we performed immunofluorescence to image the microtubule structure of ATC cells following treatment, and we found that neither caged RABiT-MMAE nor RT given individually had large effects on microtubule structure (Figure 3F). In contrast, RABiT-MMAE combined with RT destabilized cellular microtubules, similar to that seen with the fully uncaged MMAE positive control. We used the TUNEL (terminal deoxynucleotidyl transferase dUTP nick end labeling) assay to assess DNA fragmentation resulting from apoptosis following RABiT-MMAE treatment, and we found that RT restored the ability of RABiT-MMAE to elicit apoptosis, similarly as seen with the fully uncaged MMAE positive control (Figure 3G). These data indicate that RT uncages the RABiT-MMAE payload and restores its ability to destabilize microtubules and induce apoptosis in cancer cells.

### Computational Pharmacokinetic/Pharmacodynamic Modeling Reveals *In Vivo* Mechanisms of Prodrug Activity

**Figure 4:**
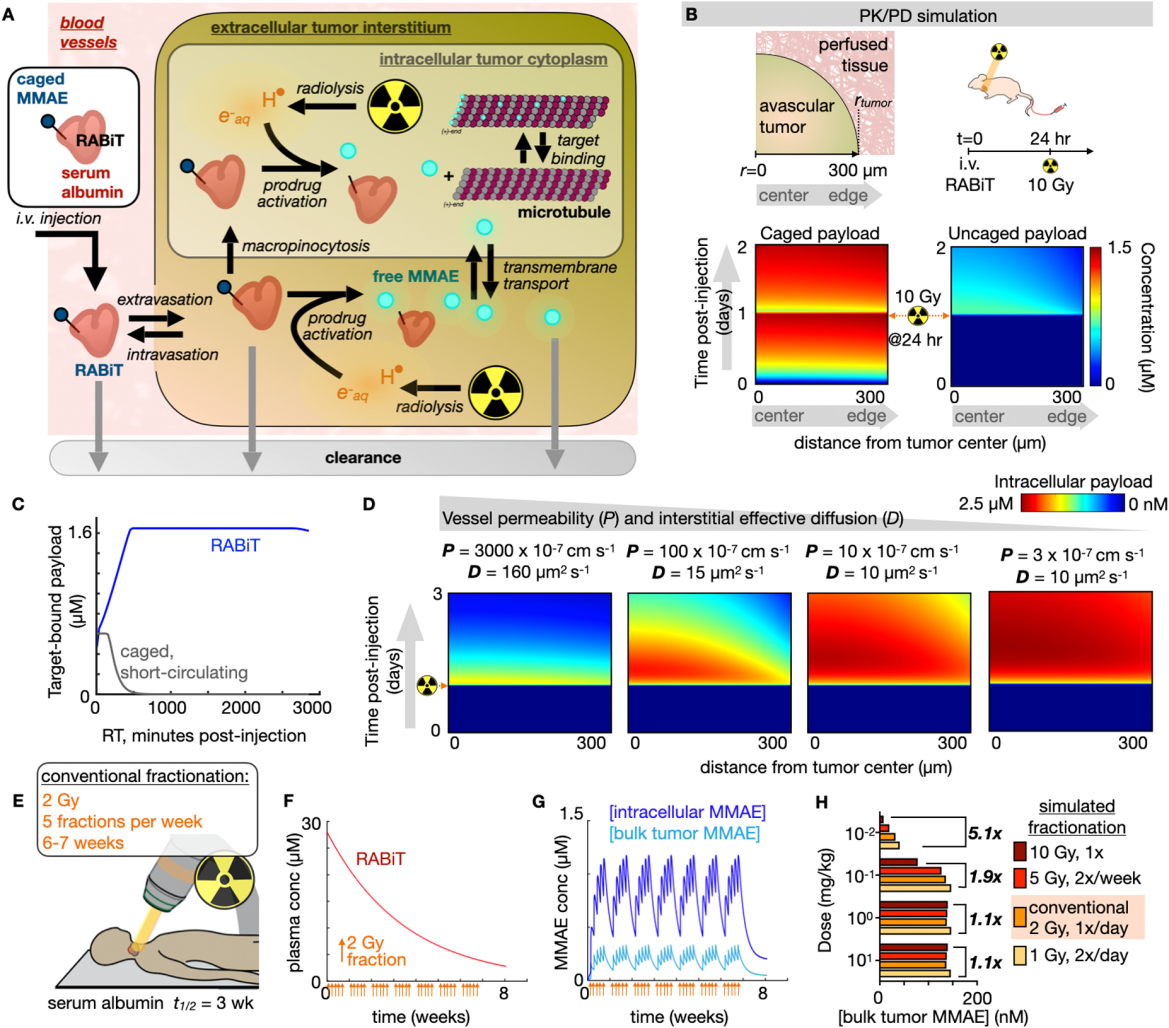
Quantitative pharmacokinetic and pharmacodynamic (PK/PD) modeling of radiation-activated prodrugs. (A) Overview schematic of the reaction/diffusion multicompartment model of RABiT activity within tumor tissue. (B) Model simulation of RABiT payload delivery in tumor-bearing mice, based on intravenous administration followed by 10 Gy irradiation 24 h later. Tumor geometry and dosing schedule (top) match the simulation results of the intact RABiT construct (left heatmap) and the uncaged, released payload (right heatmap). (C) Simulated intracellular concentration of uncaged MMAE binding to its microtubule target in tumors, averaged across 24 h following 10 Gy irradiation. (D) Simulated accumulation of uncaged RABiT payload across pairs of vessel permeability and interstitial diffusion that roughly mirror values, from left to right, of doxorubicin, MMAE, albumin, and monoclonal antibody. (E) Model translation to human pharmacokinetics and a conventional clinical radiotherapy regimen. (F, G) Simulated concentrations of circulating intact RABiT (F) and activated payload in tumors (G) during a conventional dose fractionation regimen. (H) Simulation relating dose fractionation regimens with active MMAE concentration, averaged over 1 week of treatment.

We developed a computational framework to understand localized prodrug activation *in vivo*. We first considered the degree to which RT-mediated drug activation within blood vessels contributes to on-target delivery of the tumor. The vessel volume fraction (VVF) of tumors is typically 1–10%.^48−52^ When extravascular tissue prodrug concentrations are in excess based on the *G*-value (170 nM Gy^–1^), radiolysis products become the limiting factor in determining active payload yields. Under these conditions, which we observe for RABiT (Figure 4), most payload is therefore theoretically activated extravascularly.

For payloads that are nevertheless activated within vessels, the second consideration is how much reacts locally with nearby tissue before flowing elsewhere in the body. The “extraction fraction” (EF) of a molecule is related to its “vessel depletion number” and is an estimate of the ratio of extravasation from vessels into extravascular tissue compared to convective transport through and away from tissue due to blood flow.^21,53^ The EF can be defined by the following equation:
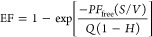
where *P* is the vessel permeability, *F*_free_ is the fraction unbound drug, *S*/*V* is the ratio of vessel surface area to tissue volume, *Q* is the blood flow rate, and *H* is the hematocrit. The EF approaches 1 for small-molecule drugs with high permeability in tissues with many small-diameter vessels and lower perfusion, and such drugs transport from blood into surrounding tissue on their first pass. However, many molecules exhibit lower vessel permeability and/or high plasma protein binding, which can decrease their EF to <10%, including as estimated for MMAE (Figure S5A). EF becomes even lower for poorly vascularized tumors with low vascular surface area relative to their perfusion rates (Figure S5B).

Based on the above analysis, we constructed a simplified multicompartment reaction/diffusion model of prodrug PK/PD that describes the spatial distribution of caged RABiT-MMAE and its uncaged payload in tumors following RT treatment (Figure 4A). We parametrized the model using known rate constants, geometries, and pharmacokinetic values from the literature (Table S1). Plasma concentration profiles, *G*-values, and biodistribution measurements for RABiT were also used (Table S2). We modeled tumor lesions as simplified avascular spheres surrounded by well-perfused tissue and vasculature. The model accurately captures the gradual accumulation of RABiT into tumor tissue, the release of active MMAE payload with radiation, its binding to microtubules, and the eventual clearance of MMAE (Figure 4B).

We used the PK/PD model to estimate how the extended circulating half-life of RABiT affects on-target drug binding in irradiated tumors. Since albumin circulates for days in mice and gradually accumulates in tumor tissue, the model predicted that on-target prodrug activation increases with greater delays between the time of RABiT injection and the time of RT, plateauing after roughly 8 h once RT-generated radicals become rate-limiting (Figure 4C). In contrast, a model small molecule performs best when irradiated quickly after or during infusion. These results suggest that the extended circulating half-life of RABiT promotes its ability to deliver active drug to tumor tissue, without requiring careful co-timing of drug and RT administration, and in principle supporting serial RT dose fractions.

We performed parametric sensitivity analysis to assess how individual model features and rate constants affected the overall prodrug activity. Simulations were recomputed after iteratively adjusting each rate constant by ±10% (Figure S6). Most adjustments yielded intuitive responses; for instance, decreasing the dissociation *k*_off_ rate of MMAE to microtubules was modeled to increase overall target engagement following RT. In contrast, even though low vessel permeability (*P*_payload_) and low effective interstitial diffusion (*D*_eff_) are often barriers to traditional drug delivery in tumors, the model predicted the opposite for the case of RABiT. Slowly transporting materials are able to gradually accumulate in tumor tissue due to the sustained circulating half-life of RABiT, and low *P*_payload_ and *D*_eff_ retain uncaged payload locally at the tumor site once activated. To illustrate, we simulated RABiT behavior with adjustments to *D*_eff_ and *P*_payload_ (Figure 4D), holding all other parameters constant. Diffusion and permeability are typically correlated, and so we used pairs of *D*_eff_ and *P*_payload_ values relevant to various model compounds, ranging from doxorubicin (*P* = 3000 × 10^–7^ cm s^–1^, *D* = 160 μm^2^ s^–1^) as a permeable small molecule at the high end to MMAE and albumin as intermediate representatives and to a monoclonal antibody at the low end (*P* = 3 × 10^–7^ cm s^–1^, *D* = 10 μm^2^ s^–1^). Overall, this modeling indicates that RABiT supports the use of lower-permeability drug payloads and a broad range of co-timing with RT treatments.

We used the computational model to predict how RABiT may perform under ideal clinical scenarios in adult patients. Serum albumin circulates for roughly 3 weeks in humans, and definitive RT treatment regimens typically involve dose fractionation of 1.8–2 Gy per day, 5 days per week, for 6–7 weeks. By recalibrating model parameters to an adult patient undergoing such conventional RT fractionation, the simulations predict that a single infusion of RABiT could circulate in the body long enough to support serial drug activation responses to daily RT fractions (Figure 4E–G). Intratumoral active MMAE cumulatively increases with daily weekday RT and stably oscillates as levels decrease during weekend treatment gaps (Figure 4G). Although daily ∼2 Gy fractions are most widely used clinically, other hyperfractionated, hypofractionated, and single-dose ablative regimens are possible. Simulations indicate that RT given more frequently and at lower doses will promote more evenly sustained tumor drug concentrations and will promote higher average concentrations under some dose scenarios (Figure 4H). This latter effect is due to transient and localized depletion/consumption of RABiT during RT: dividing RT treatments into multiple smaller fractions allows RABiT to locally replenish as it continually circulates in the body. In contrast, fewer larger fractions of RT achieve higher maximum concentrations of active drug at high RABiT doses when RT-generated radicals are limiting (Figure S7A). Regardless, the model predicts feasibility in achieving MMAE concentrations that are cytotoxic to a broad range of murine and especially human cancer cell models (Figure S7B,C). The computational model thus offers guidance on maximizing RABiT efficacy using clinically relevant RT fractionation schemes and highlights its potential for sustained and cumulative drug release.

### RABiT Exhibits a Sustained Circulating Half-Life and Efficiently Accumulates in Tumor Tissue

**Figure 5:**
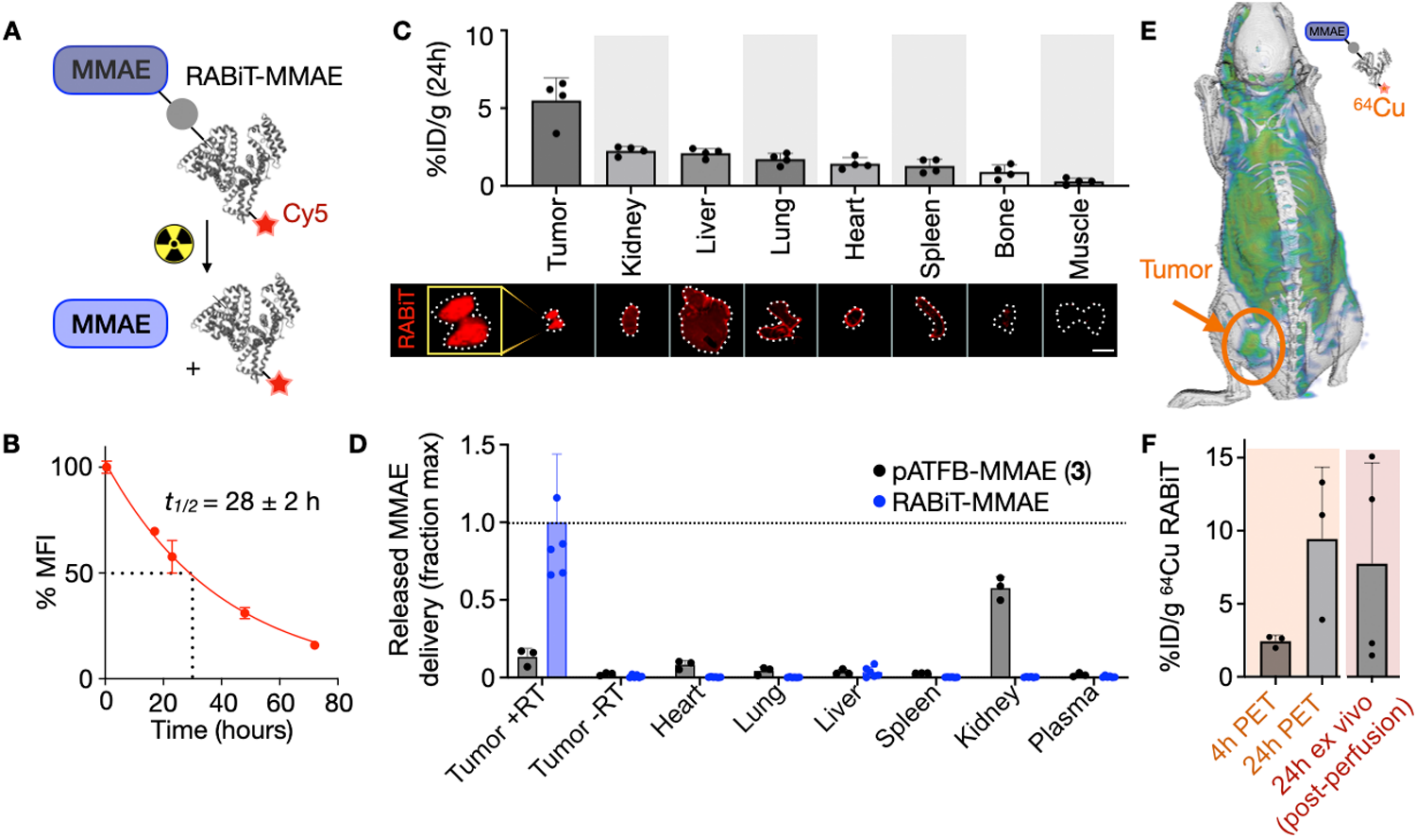
RABiT pharmacokinetics and biodistribution. (A) Schematic of Cy5 labeled RABiT-MMAE. (B) Blood concentration of fluorescent RABiT-MMAE following intravenous injection into naïve mice. (C) Representative imaging and quantification of fluorescent RABiT-MMAE in mice bearing TBP3743 ATC tumors, 24 h post-injection; scale bar = 5 mm. (D) LC/MS quantification of free active MMAE from tissue, 24 h post-injection and immediately following 10 Gy tumor irradiation. Data normalized to max value measured across all tissues (irradiated tumor). (E) *In vivo* PET/CT imaging of RABiT-MMAE labeled with the Cu-64 PET tracer. (F) Quantification of RABiT-MMAE Cu-64 levels in tumors based on PET imaging or by *ex vivo* analysis of dissected tumor tissue. Data are means ± SEM.

We next investigated the sustained *in vivo* ability of RABiT to accumulate in tumor tissue. All animal experiments were performed in accordance with guidelines from the Institutional Subcommittee on Research Animal Care. We used Cy5-NHS (*N*-hydroxy succinimide) to label albumin and form a fluorescent RABiT (Figure 5A), which exhibited a circulating blood *t*_1/2_ of 28 ± 2 h in healthy C57BL/6J mice (Figure 5B), comparable to native mouse serum albumin (MSA; *t*_1/2_ = 35 h); the ortholog human serum albumin (HSA) in humans circulates for 3 weeks.^54^ After flushing mouse vasculature with PBS to flush tissues of blood, we found that fluorescent RABiT accumulated in an anaplastic thyroid cancer (ATC) model at 5.5 ± 1.4% injected dose per gram (% ID/g) tissue, 24 h post-injection (TBP3743 tumors in B6129SF1/J mice) (Figure 5C).^39^ RABiT thus exhibited favorable and sustained pharmacokinetics and biodistribution.

We next measured the *in situ* RABiT payload release in irradiated tumors. RABiT-MMAE or pATFB-MMAE (**3**) was intravenously injected in ATC tumor-bearing mice. Then, 24 h later, isolated tumors on one side were irradiated with 10 Gy (320 keV), and tissues were immediately processed for LC/MS quantification of RABiT payload release. Irradiation increased free MMAE by 130-fold compared to levels in contralateral nonirradiated tumors (Figure 5D). Furthermore, 7.5-fold more free MMAE was found in irradiated tumors using RABiT compared with pATFB-MMAE (**3**). Additionally, pATFB-MMAE (**3**) showed higher off-target free drug activity in the kidney (180-fold), heart (30-fold), and lung (30-fold). Albumin conjugation in RABiT thus promoted selective and efficient tumor delivery while minimizing undesired off-target activation.

To confirm the extended pharmacokinetics of the RABiT-MMAE platform, we performed *in vivo* PET/CT (positron emission tomography/X-ray computed tomography) imaging of labeled RABiT-MMAE in tumor-bearing mice. We stably conjugated the PET tracer chelator, DOTA (1,4,7,10-tetraazacyclododecane-1,4,7,10-tetraacetic acid) to RABiT-MMAE albumin using NHS-ester, chelated with Cu-64, and imaged distribution at 4 and 24 h post-injection (Figure 5E). PET/CT indicated RABiT tumor accumulation gradually increased from 2.4% ID/g at 4 h to 9.4% ID/g at 24 h, and such delayed uptake is consistent with extravascular accumulation. Reinforcing this point, RABiT uptake remained similar following cardiac perfusion to flush blood from tissue, followed by tumor dissection and Cu-64 quantification by gamma counting (Figure 5F); furthermore, tumor accumulation was not significantly different from that of the fluorescent RABiT, also measured after flushing blood from tissue (Figure 5C, *P* = 0.73, Kruskal–Wallis test and Dunn’s multiple comparisons test). High off-target RABiT levels at 24 h, as seen by PET/CT (Figure 5E), are consistent with the high RABiT concentration in blood, estimated as 38% ID/g (Figure 5B). Taken together, these data indicate RABiT is long-circulating, gradually accumulates in tumor tissue, and can be selectively triggered by radiation to activate payload locally despite high levels in off-target tissues and blood.

### RABiT Safely Combines with RT to Block Tumor Progression with Mitigated Toxicity

**Figure 6:**
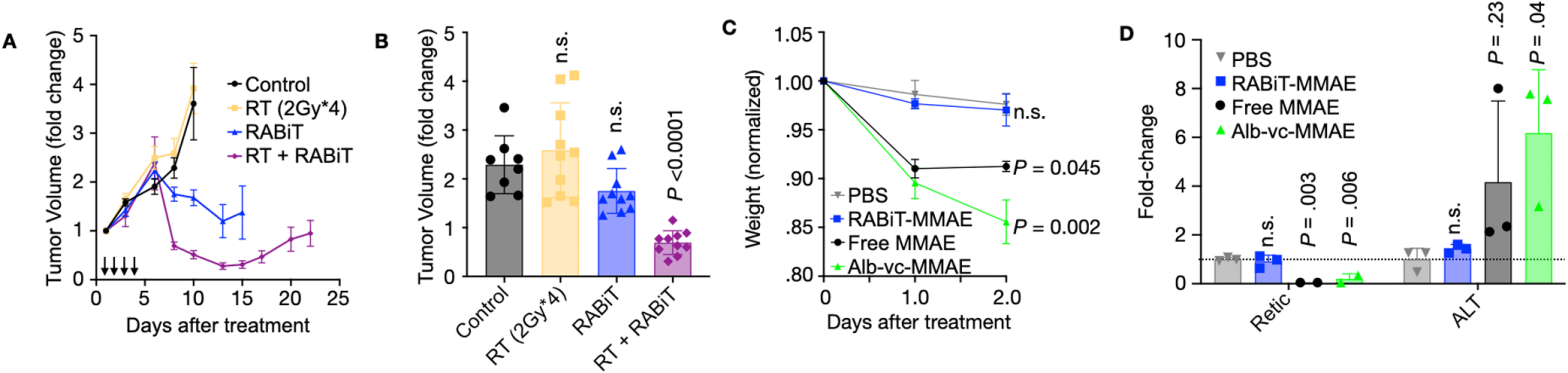
RABiT *in vivo* efficacy and safety. (A, B) Mice bearing TBP3743 ATC tumors were treated with 13.9 μmol kg^–1^ RABiT-MMAE and/or 2 Gy X-ray radiation for 4 consecutive days, and growth was monitored by caliper over time (A) and shown with individual tumor sizes on day 8 (B). *n* = 17 total mice (34 total tumors). (C–E) Naïve C57BL/6 mice receiving 13.9 μmol kg^–1^ RABiT-MMAE, Alb-vc-MMAE, or free MMAE were weighed (C), and after 2 days, blood was analyzed for alanine transaminase (ALT) and reticulocyte count (D). Data are means ± SEM.

RABiT is long-circulating, and our PK/PD modeling suggested RT could be effective given hours or even days following RABiT administration. Therefore, we hypothesized that RABiT could be efficiently combined with fractionated RT to control tumor progression in tumor-bearing mice. Upon formation of syngeneic ATC tumors, mice were treated with 13.9 μmol kg^–1^ (10 mg kg^–1^ MMAE equivalent) RABiT followed by 2 Gy conformal RT (in a 320 keV small animal radiation research platform) 3 h later for 4 consecutive days. RABiT synergistically blocked tumor growth when combined with RT (*P* < 0.002, two-way ANOVA interaction term) (Figure 6A,B). At this dose, the prodrug alone had some effect (*P* = 0.02), which may be attributed to the basal activity of RABiT metabolites, degradation products, and low but detectable nonirradiated payload release. All treatments showed no obvious toxicities or weight loss (Figure S8A).

We evaluated the acute toxicity of RABiT compared to formulations delivering MMAE payload via a valine–citrulline cathepsin cleavable linker to serum albumin (Alb-vc-MMAE) or as a free unconjugated drug. Mice were given a single 13.9 μmol kg^–1^ MMAE dose, delivered as RABiT-MMAE, Alb-vc-MMAE, or solvent MMAE by intravenous injection. Body weight dropped for Alb-vc-MMAE and solvent MMAE but not for RABiT (Figure 6C). On day 2, animals were autopsied for toxicity evaluation. Alb-vc-MMAE and solvent MMAE, but not RABiT, increased alanine transaminase (ALT; *P* = 0.043) and decreased reticulocyte counts (*P* = 0.005), which indicated hepatotoxicity and bone marrow toxicity, respectively (Figures 6D and S8B,C). This experiment, combined with our prior data,^39^ indicated a maximum tolerated dose of Alb-vc-MMAE of roughly 1.0–2.0 μmol kg^–1^; 5–10× this dose-equivalent of RABiT showed no acute toxicity in mice, and a cumulative 20–40× dose showed no apparent effect on body weight (Figure S8). These data indicate RABiT payload caging stably blocks toxicity in nonirradiated tissues compared to uncaged and cathepsin-caged formulations.

## Discussion

This work shows RABiT promotes efficient *in situ* local drug activation in tumor tissue and expands radiation-activatable chemistry to fractionated RT and ion beam therapy using high-energy protons. We use a combination of tissue imaging, LC/MS, and computational modeling to measure and interpret the biodistribution of the RABiT vehicle and the controlled release of its payload by radiation. RABiT is based on bridging potent drug payloads to serum albumin via a radiation-labile self-immolative linker, and we demonstrate RABiT as highly stable *in vivo*, showing very little off-target drug release or off-target toxicity in nonirradiated tissues. We observe that linking prodrug to serum albumin achieves several goals: (i) it extends the circulation half-life and enables RABiT to accumulate at higher levels and for a longer time period in tumor tissue. (ii) It protects the prodrug from premature renal clearance and off-target metabolism and unintended activation. Lastly, (iii) it fully cages drug activity by not just blocking the ability of drug payload to engage its target but also blocking the ability of payload to reach the appropriate cell compartment (for instance, the nucleus for doxorubicin). The RABiT platform accommodates diverse drug payloads and may have particular value in promoting the localized delivery of molecules that have low cell permeability, poor circulating pharmacokinetics, and susceptibility to off-target metabolism or sequestration in the body.

Serum albumin has several advantages as a platform for long-circulating radiation-activatable drug delivery. Notably, 65 kDa RABiT is anticipated to have an 8 nm hydrodynamic diameter compared to 10–15 nm for ∼150 kDa antibody-drug-conjugates and 80–120 nm for clinical PEGylated liposomes and, therefore, may penetrate tumor tissue more uniformly.^55^ Albumin accumulates via oncogene-driven macropinocytosis in multiple cancer types,^39,56^ and our experiments in mice indicated its tumor uptake can approach levels achievable with some molecularly targeted methods. This is valuable since no known antibody-drug-conjugates have shown clinical success for treating ATC and many other relevant aggressive cancer types. Although we have found that most serum albumin within tumor tissue has been taken up by cells in various mouse models by 24 h post-injection,^40,56^ including in ATC,^39^ serum albumin is known to bind proteins associated with the extracellular matrix, including osteonectin/SPARC (secreted protein acidic and rich in cysteine). Traditionally, this extracellular sequestration would be detrimental to drug delivery and would prevent the drug payload from being internalized by cancer cells and released, for instance, by endolysosomal cathepsin-mediated cleavage. In contrast, the RABiT platform does not require cellular internalization for payload release and activation. In this work, we demonstrate RT activates payloads both in serum media (Figure S1C) and once RABiT has been taken up by cells (Figure 3F,G). Computational modeling indicates contributions to activity from both interstitial/extracellular and intracellular pools of RABiT (Figure 4). This feature may be important in expanding the types of molecular targeting strategies that may be considered in the future, for instance, by targeting components of the tumor-associated extracellular matrix itself^35^ or non-internalizing molecules that are overexpressed on the cancer cell surface. Payloads such as MMAE are cell-permeable once released from their drug carriers and can promote bystander killing of neighboring cells.^57^ The radiation-activated strategy may therefore be relevant for targeting tumor-associated stromal cells via targets such as fibroblast activation protein (FAP),^58^ targeting tumor-associated macrophages that phagocytose albumin and other drug delivery vehicles,^59,60^ and potentially in concert with delivering therapeutic radionuclides to targets such as FAP and somatostatin receptor subtype 2 (as with clinically used lutetium Lu-177 dotatate).

This work presents a quantitative PK/PD framework for understanding mechanisms of localized drug delivery using radiation-activated prodrugs, and we use it to interpret experimentally observed RABiT behaviors *in vivo*, to extrapolate results to different types of drug payloads and behaviors in humans, and to guide further experiments. We encouragingly found that RABiT is tolerated in mice at high doses that allow it to be delivered far in excess of RT-generated radical species that serve as reactants for prodrug activation: *G*-values of ∼170 nM Gy^–1^ were observed in cell culture, and RABiT biodistribution measurements revealed a ∼5.5% ID/g tumor uptake, which correlates to a roughly 10 μM prodrug concentration. With this RABiT dose, measurements therefore suggest that H_2_O radiolysis products are limiting at nearly all clinically relevant RT doses (<50 Gy). Under this regime, the vessel volume fraction combined with the extraction fraction of drug payload (VVF × EF) indicates that extravascular rather than intravascular prodrug contributes most to the overall pool of RT-activated payload that accumulates in tumor tissue. The PK/PD model reflected this finding by showing that RABiT becomes more efficient as more time elapses between its systemic administration and local RT treatment. Perhaps counterintuitively, the model showed that sustained RABiT pharmacokinetics allowed payloads with low diffusivity and permeability to accumulate in tumor tissue and be retained there locally once activated by RT. This suggests that RABiT or similar strategies for extending pharmacokinetics will be important for prodrug payloads that are generally larger in molecular weight and/or show high protein binding. We anticipate the PK/PD framework will be useful in understanding diverse RT-activated prodrug strategies using distinct carrier vehicles such as antibodies, liposomes, and peptides, as well as distinct small-molecule, peptide, and protein-based therapeutic payloads.

We acknowledge limitations to the study presented here. The computational PK/PD model helped interpret mechanisms of RABiT drug delivery but made several simplifying assumptions. Free MMAE is known to rapidly clear from the blood,^61^ and we therefore focused the computational model on extravascular drug behaviors in the tumor. Free MMAE in the blood was modeled as being cleared before extravasation into tumor tissue. Future work may consider modeling accumulation of released payload in off-target tissues and fluids, including the blood, liver, kidney, and other sites, such as the bone marrow, that are known sites of payload toxicity. Future studies may also model background rates of drug metabolism and activation in nonirradiated tissue, more detailed tumor/vascular geometries, intratumor heterogeneity in drug distribution, and an examination of human PK/PD with such considerations. We use a syngeneic immune-competent mouse model to evaluate RABiT, and our prior work examining albumin delivery in this model has shown low levels of uptake in lymphocytes compared to tumor cells,^59^ therefore raising the possibility that RABiT may be less immunosuppressive compared to traditional chemoradiation. RT and MMAE can combine to stimulate immune responses,^61^ and future work should investigate how RABiT may impact the immune response and potentially synergize with immunotherapy. In this work, we show *in vitro* how multiple forms of ionizing radiation, including from clinical linac and proton beam sources, are comparable in their ability to trigger prodrug release. This sets the stage for future RABiT evaluation in orthotopic disease models and with clinical RT sources used *in vivo*, including through the use of proton irradiation and MV linear accelerators.

Overall, this work presents the design, computational analysis, and *in vivo* proof-of-principle demonstration of RABiT as a safe and effective long-circulating platform for the localized delivery of chemotherapy drugs specifically to irradiated tumor tissues. The generalized approach is poised for future studies that extend to new designs and biomedical applications as well as studies to prepare for clinical translation.

## Supporting Information Available

Experimental materials and methods, chemical syntheses and characterizations including NMR spectra, Figures S1–S8, and Tables S1 and S2 (PDF)
