# Supplementary material for: Extended Pharmacokinetics Improve Site-Specific Prodrug Activation Using Radiation
Source: ACS Cent Sci. 2024 Jun 21;10(7):1371–82. doi: 10.1021/acscentsci.4c00354 (PMC11273447; doi:10.1021/acscentsci.4c00354)
Supplement: Supplementary file 1 — oc4c00354_si_001.pdf [file oc4c00354_si_001.pdf]

# Supporting Information

**Extended pharmacokinetics improve site-specific prodrug activation using radiation**

Jeremy M. Quintana[1,4], Mikyung Kang[1,4,6], Huiyu Hu[1,2], Thomas S.C. Ng,[1,4] Gregory R. Wojtkiewicz[1,] Ella Scott,[1] Sareh Parangi[2], Jan Schuemann[3], Ralph Weissleder[1,4,5]*, Miles A. Miller[1,4]*

**Affiliations**

[1] Center for Systems Biology, Massachusetts General Hospital Research Institute, Boston, MA 02114

[2] Department of Surgery, Massachusetts General Hospital and Harvard Medical School, Boston, MA 02114

[3] Department of Radiation Oncology, Massachusetts General Hospital and Harvard Medical School, Boston, MA 02114

[4] Department of Radiology, Massachusetts General Hospital and Harvard Medical School, Boston, MA 02114

[5] Department of Systems Biology, Harvard Medical School, Boston, MA 02115

[6] Current address: School of Health and Environmental Science, College of Health Science, Korea University
145 Anam-Ro, Seongbuk-Gu, Seoul, 02841, Republic of Korea

* Correspondence:
Miles A. Miller, PhD
Center for Systems Biology
Massachusetts General Hospital
185 Cambridge St, CPZN 5206
Boston, MA, 02114
617-726-8226
miles.miller@mgh.harvard.edu

Ralph Weissleder, MD PhD
Center for Systems Biology
Massachusetts General Hospital
rweissleder@mgh.harvard.edu

**Contents**

1. Methods
2. Supplemental Figures
3. Organic Synthesis
4. Characterization
5. References

# 1.Methods

***Irradiations:*** A total of four irradiation modalities were used:

*X-ray irradiation*: Unless stated otherwise, X-ray irradiation used a X-RAD 320 cell and small animal irradiator with a 320 keV energy and dose rate of 325 ± 10 cGy $min^{-1}$ at room temperature (Precision X-ray). Parameters for X-ray irradiation were 320 kV, 12.5 mA, and HVL ~ 1mm Cu.

*Gamma irradiation*: Irradiation was performed on a dual source $^{137}$Cs Gammacell 40 Exactor (Best Theratronics) with a dose rate of roughly 50 cGy/min.

*Protons*: Proton beam irradiations were performed at the Francis H. Burr Proton Therapy Center with a single field impinging vertically on the samples. The samples were placed at the center of the spread-out Bragg peak (SOBP) with a range of 13 cm and a modulation width of 7 cm, resulting in a flat dose distribution at and around the sample with an LET ~2.3 keV/*µ*m and a dose rate of ~0.5 Gy/s. The LET was estimated using Monte Carlo simulations with TOPAS which has previously been well tested for the FHBPTC beamline.[1, 2]

*MV Linac*: For clinical energy (6 MV) photon irradiations were performed at the MGH Clark Center in the Lunder building using Varian Truebeam Linacs. A solid water phantom size of 5 or 10 cm depth was used to simulate the internal scatter expected with biological tissues.

***Drug release measurement:*** 10 µM solutions of each prodrug were prepared in PBS (pH 7.4, <1% DMF), in 96 well plates. After irradiation, samples were analyzed by LC/MS on a Waters instrument equipped with a Waters 2424 ELS Detector, Waters 2998 UV-Vis Diode array Detector, and a Waters 3100 Mass Detector. Samples were run on an XTerra MS C18 column

(125., 5 µm, 4.6 mm X 50 mm column) using a gradient of 5-95% acetonitrile in water (0.1% formic acid) over 1.5 minutes, followed by 95% acetonitrile for 0.5 minutes at a flow rate of 5 mL/min. Drug concentrations were determined by comparing the AUC from the ELSD or isolated mass chromatographs (+ESI, 718.8 Da for MMAE, 544.4 Da for DOX) of each sample to a standard calibration curve (7 points minimum). Redox conditions were compared by preparing the above samples in either 10 mM sodium formate, 10 mM potassium formate, 10% hydrogen peroxide, or 0.01 M sulfuric acid in 1 M *tert*-butanol. These samples were then irradiated and analyzed using the same LC/MS analysis methods described above.

***RABiT preparation and cytotoxicity assay:*** RABiT-MMAE/DOX was prepared by adding 2.25 mg of the respective prodrug (**4** or **5**, as a solution in 100 µL dry DMF) to a solution of 100 mg mouse serum albumin (Msa) in 1 mL PBS (pH 7.4). This mixture was shaken for 2 hrs at room temperature, then filtered to remove unconjugated prodrug using a 10 kDa MWCO spin filter. The final conjugate solution was stored at 4°C until further use. Cytotoxicity experiments were performed by seeding 5,000 cells per well overnight in a 96-well plate (Corning) before adding of each drug/conjugate. MMAE or prodrug (with or without 10 Gy radiation to potentiate drug release) were prepared at varying concentrations (100 µM and subsequent 5-fold serial dilutions down to 0.2 nM) in media in a 96-well plate. The drug/prodrug solutions were then added to the respective wells containing cells (20 µL into 180 µL to achieve a 10-fold dilution). Empty wells with only media or vehicle-treated cells were used as controls. After the addition of the corresponding drugs and a 72 hr incubation, the quantity of live cells was determined by PrestoBlue (ThermoFisher, USA) according to the provider's protocols.

***Rad-Act-mAb preparation:*** Trastuzumab (15 mg, 0.1 µmol) was dissolved in PBS (1 mL) containing 1 mM tris(2-carboxyethyl)phosphine (TCEP) and incubated for 2 hours at room temperature. The TCEP was removed via spin filtration (50 kDa MWCO spin filter, 7000 rcf for 5 min x 3) and the protein was diluted to 1 mL in PBS before prodrug **4** was added to achieve a final concentration of 400 µM. This mixture was shaken vigorously for 2 hours at room temperature, then filtered via spin filtration (50 kDa MWCO spin filter, 7000 rcf for 5 min x 5) to remove unbound prodrug. The final solution was diluted in PBS to achieve a protein concentration of 100 µM as determined via UV/vis absorbance on a Nanodrop ND-1000 spectrophotometer.

***Rad-Act-NP preparation:*** A solution of prodrug **4** (1 mM) and thiol poly(ethylene glycol)-*block*-poly(lactide-*co*-glycolide) (2 mM, PEG average Mn 5000, PLGA Mn 15,000, lactide:glycolide 50:50, Sigma Aldrich, USA) in 100 µL DMF was incubated for 2 hours at room temperature before the addition of a further 100 µL of 8 mM poly(ethylene glycol)-*block*-poly(lactide-*co*-

glycolide) (PEG average Mn 5000, PLGA Mn 15,000, lactide:glycolide 50:50, Sigma Aldrich, USA). This mixture was added dropwise to a conical vial containing 50 mL of nanopure water. This suspension was then shaken at room temperature for 4 hours before it was concentrated via spin filtration (30 kDa MWCO spin filter, 3000 rcf for 30 min). The final suspension was diluted to a final volume of 1 mL in PBS.

***Prodrug 4-conjugate characterizations:*** the degree of labeling (DOL) was determined by treating an aliquot of each conjugate (100 μM) with 10 mM TCEP in PBS for 30 minutes at room temperature. The solution was then analyzed by LC/MS to quantify the amount of MMAE released (**Fig. S3a**), which was determined by comparison to a standard calibration curve, as described above. This process was repeated for the polymer nanoparticles to determine the drug:polymer ratio, based on the initial concentration of polymer. The polymer nanoparticles were analyzed via size and zeta potential measurements on a Malvern Zetasizer Nano-ZS before and after incubation at 37°C in PBS (pH 7.4) over 72 h. Measurements were collected in triplicate, and reported as mean ± s.e.m (**Fig. S3b**).

***Colony formation assay:*** $1 \times 10^4$ TBP3743 cells were placed in each well of 96-well plates and allowed to incubate overnight. Subsequently, the cells were treated with either a control solution or RABiT conjugate, followed by a 6-hr incubation period. The cells were then exposed to 8 Gy X-ray (320 kV, 12.5 mA). Following a 48-hr incubation post-treatment, the cells were harvested and re-seeded into individual wells of a 6-well culture plate in fresh medium at a concentration of 200 cells/mL, allowing them to form colonies over a period of 1 to 3 weeks. Once colonies had developed, they were fixed with 100% methanol for 20 minutes, stained with a crystal violet staining solution (Sigma) for 20 minutes at room temperature, and then rinsed with distilled water. Plating efficiency (PE) and survival fraction were calculated as previously described previously[3]: Survival fraction = (number of colonies formed after treatment) / (number of cells initially seeded × PE) × 100%, Plating efficiency (PE) = (number of colonies formed for untreated cells) / (number of cells initially seeded) × 100%. The plating efficiency for cells not exposed to irradiation was determined to be 95%.

***Immunofluorescence and TUNEL assays:*** $1 \times 10^4$ TBP3743 cells were seeded in each well of 96-well plates overnight. TBP3743 cells were then treated with vehicle, RABiT or MMAE (at a concentration of 100 nM). After 6 hrs, the cells were washed three times with PBS, replaced with fresh cell culture media, irradiated with 10 Gy X-rays, and incubated for 24 (for TUNEL marker staining) or 48 hrs (for α-tubulin). Cells were then fixed with 4% PFA for 30 mins at room temperature, washed in PBS, permeabilized in 0.5% Triton

X-100 in PBS for 30 mins, and blocked with a 10% serum from the same species as the secondary antibody for 20 mins at room temperature. α-tubulin Alexa Fluor 488 mouse monoclonal antibody (DM1A, 1:100, Invitrogen) were used for staining. Apoptotic cells in the tumors were determined using an DeadEnd Fluorometric TUNEL System (Promega) according to the manufacturer's instructions. All samples were mounted with mounting solution containing DAPI to stain the nuclei and were imaged using a fluorescent microscope (Revolve, Discover Echo). Images were analyzed using ImageJ to determine the relative amount of α-tubulin (normalized to the vehicle control) or the percentage of positive cells in each image.

***Mouse experiments and cell culture:*** All animal research was performed under guidelines from the Institutional Subcommittee on Research Animal Care and with approval from the Institutional Animal Care and Use Committee at Massachusetts General Hospital. Mice were housed in pathogen-free vivarium with controlled temperature, humidity, and light/dark cycling. Syngeneic mouse cell lines underwent mouse pathogen testing (IDEXX) before use and were subjected to routine testing for mycoplasma contamination (Mycoplasma PCR test Kit, Applied Biological Materials). MC38 cells were provided by M. Smyth (Peter MacCallum Cancer Centre, Victoria, Australia), while TBP3743 cells were prepared and cultured as previously described.[4] Briefly, cells were cultured in DMEM with 10% FBS and penicillin/streptomycin under an atmosphere of 5% $CO_2$. Nu/nu mice were obtained from COX-7 at Massachusetts General Hospital, while B6129SF1/J and C57BL/6J mice were purchased from the Jackson Laboratory (JAX).

***In vivo anti-tumor efficacy assay:*** A total of $5 \times 10^5$ TBP3743 cells in PBS were implanted subcutaneously into the flanks of female B6129SF1/J mice 6-10 weeks old. When tumors were palpable (3-4 mm diameter), mice were randomized and injected intraperitoneally with vehicle control or RABiT-MMAE (10 mg $kg^{-1}$ free MMAE) in 100 µL PBS. 6 hrs later, mice were anesthetized with intraperitoneal injection of xylazine (2 mg/kg) and ketamine (60 mg/kg) to enable for local x-ray irradiation of 2 Gy to subcutaneous tumors. The x-ray irradiation was performed using the above conditions, with mice positioned so that the tumors were located within the corner of the irradiation field, 40 cm from the source. This treatment protocol was repeated for four consecutive days. Body weights were monitored and tumor volumes were calculated every 1-3 days using two sets of digital caliper measurements and the equation $V = 4/3\pi r^3$. Prespecified euthanasia criteria included ulceration, tumor sizes greater than 10 mm in diameter, body condition score ≤ 2, or weight loss greater than 20%. Longitudinal tumor growth was

plotted as means for each group until any animals in the group reached the predefined humane experimental endpoint. Tumor growth values of control and RT-only treatment groups are used in contemporaneous projects of the lab including Kang et al.[5]

***Cell extraction:*** For *in vitro* drug activation in cell culture, 10 cm plates of TBP3743 cells were grown to ~80% confluence before each respective drug/prodrug was added (10 μM in DMEM) . These cells were incubated for 24 hrs before being washed with fresh media then irradiated, followed by a further 3 washes with PBS and lysis via addition of 100 μL of Lysis buffer II (ThermoFisher). Using a cell scraper, the lysate was collected, diluted 4-fold with acetonitrile, and centrifuged (20,000 rcf for 5 min) to pellet the remaining cell debris and precipitated protein. The supernatant was then collected and analyzed by LC/MS to determine the amount of drug released.

***Tissue extraction:*** Tumor-bearing mice were treated with 10 mg/kg RABiT-MMAE, then monitored for 24 hrs before the tissues were harvested. Flank tumors were X-ray irradiated (10 Gy) and all tissues were placed in Lysis buffer II (ThermoFisher, 200 μL). The tissues were finely minced and incubated for 30 minutes on ice, then diluted 4-fold with acetonitrile and centrifuged (5,000 rcf for 5 min). The supernatant was filtered through 3 kDa MWCO spin filters to remove any remaining tissue fragments and small proteins. The flowthrough was analyzed by LC/MS to determine the amount of drug released. Measurements were collected in triplicate and compared to a drug calibration curve to determine concentrations, normalized to the mass of the collected tissues.

***Circulation half-life:*** RABiT-MMAE was conjugated with Cy5-NHS (Lumiprobe) in carbonate buffer (pH 8) for 1 hr prior to removal of free dye via spin filtration (10 kDa MWCO). The purified conjugate was prepared to a concentration of 200 mg/mL protein, and 100 μL was injected into each female 12 week old C57BL/6J (JAX) mouse via tail vein. Retro orbital blood samples were then collected at various times up to 72 h post-treatment (n=2 per time point). Samples were centrifuged, plasma collected and stored at 4°C until the end of the experiment, diluted 10-fold with PBS, loaded into a 96-well plate, and analyzed on a TECAN fluorescence plate reader. The mean fluorescence values were fit to a monoexponential decay to determine the circulation half-life.

***Biodistribution:*** RABIT-MMAE, modified with Cy5 as described above, was injected via the tail vein (10 mg/mL) into four 10-12 week old female B6129SF1/J mice bearing TBP3743 tumors. Tissues were harvested from the mice after 24 hours, and biodistribution measurements were made by quantifying fluorescent imaging taken on an azure sapphire. Quantification of the images was performed using imageJ to compare the relative fluorescence of each tissue to a

calibration curve constructed from known concentrations of Cy5-amine (minimum 5 point curve) as described previously.[6]

***Computational modeling.*** Computational modeling of RABiT PK/PD was performed in Matlab using coupled differential equations as described previously.[7] Tumor geometry was modeled as a symmetrical avascular sphere. Model equations and boundary conditions are depicted in Fig. 4 and as equations in Table S1. Parameter values and associated references for the model are provided in Table S2. Partial differential equations used the method of lines technique implemented in Matlab. Model assumptions include negligible transport of free payload into tumor tissue, due to low extraction fraction and short circulating half-life. Sensitivity analysis was performed by adjusting parameter values +/- 10%, recomputing model, and calculating the average caged, uncaged, and target-bound payload over the 24 h window following time of initial RT. Model translation to human pharmacokinetics scaled the RABiT circulating half-life to roughly that of humans (3 weeks) and used a plasma volume of 3.5 L for a 70 kg individual. Radiation fractionation used the following, for 7-day simulations (day 1-day 7), with RABiT administered on day 1: 1 Gy every 12 hours from day 2 to day 6; 2 Gy daily from day 2 to day 6; 5 Gy on day 2 and day 4; 10 Gy on day 2.

***In vivo drug safety evaluation:*** 10 mg/kg free MMAE or equimolar Alb-vc-MMAE, RABiT-MMAE or vehicle control were injected by tail vein into 12 week-old female C57BL/6 mice (JAX) through the tail vein. Body weights were measured daily. On the second day post-treatment, whole blood was obtained through terminal cardiac puncture under isoflurane anesthesia (2% at 1L $min^{-1}$) on a heated stage and stored in EDTA-coated tubes for downstream evaluation. Complete blood count, manual reticulocyte count, and blood chemistry panel analyses were performed by MGH Veterinary Clinical Pathology Laboratory using HemaTrue and DriChem analyzers. Blood count analyses for two samples were affected by clotting and excluded. In some cases, low sample volume allowed only partial completion of the blood chemistry panel. Initial sample sizes of n = 3 were used for all groups and dropout due to these issues is indicated in graphs, resulting in n = 2 samples in some cases. Calcium, cholesterol, globulin, and total protein were not analyzed due to insufficient sample volumes. Besides ALT and retic, values showed no statistically significant change with treatments compared to control. Alb-vc-MMAE was synthesized by adding 1 mg of vc-MMAE (0.8 µmol) to a solution of 50 mg Msa (0.8 µmol) in 1 mL PBS. The conjugate was incubated at room temperature for 2 hours, then purified by spin filtration using a 10 kDa MWCO filter (5x in PBS) and diluted to a concentration of 200 mg/mL Msa (4 mg/mL vc-MMAE). Prodrugs were injected in 20 µl DMSO and 80 µl PBS by tail vein.

***In vivo PET imaging and biodistribution:*** To a solution of RABiT-MMAE was added a solution of NHS-DOTA (6 eq.) in DMSO. This mixture was basified (pH ~8.5) via addition of saturated sodium bicarbonate, then incubated for 1 hour at room temperature before purification using a 10 kDa MWCO spin filter (12,000 rcf for 5 min x 3). The solution was then diluted in citrate buffer (pH 5) and a solution of Cu-64 (University of Wisconsin, Department of Medical Physics) was added (2 mCi/10mg RABIT). After incubation at 37°C for 45 minutes, the solution was again purified via spin filtration using a 10 kDa MWCO spin filter (12,000 rcf for 5 min x 3). The purified protein was diluted in PBS to a concentration of 3 mCi/mL (>95% of Cu-64 chelated), then injected intravenously into mice bearing TBP3743 tumors. Mice were intravenously injected with ~250 uCi of probe and imaged at 4 hr and 24 hrs post injection on a Siemens Inveon Postron Emission Tomography (PET) and Computed Tomography (CT) small animal imaging system.  The acquisition time for the positron emission tomography scans was 30 min for the 4hr and 60 min for the 24hr time points and the images were reconstructed with the OSEM/3DMAP (ordered subsets expectation maximization using maximum a posteriori) algorithm with 2 OSEM iterations and 20 MAP iterations.  Computed Tomography (CT) scans were imaged over 360 projections and reconstructed into 110 μm isotropic voxels using a modified Feldkamp conebeam reconstruction algorithm (Cobra, Exxim Inc.). PET-CT images were registered using an a priori rigid affine transformation and, to take into account animal movement since two animals were imaged during a scan, the registration was further refined by deleting the other mouse from the scan and reregistering the scans using a rigid normalized mutual information algorithm. Standard uptake values were calculated from the PET scans analyzed by manually drawing regions of interest (ROI) on the corresponding CT.  3D image renderings were performed in the Amira software environment. Tumor tissues were harvested and the radiation was quantified on a 1480 Wizard 3 (PerkinElmer) gamma counter.

# 2.Supplemental Figures

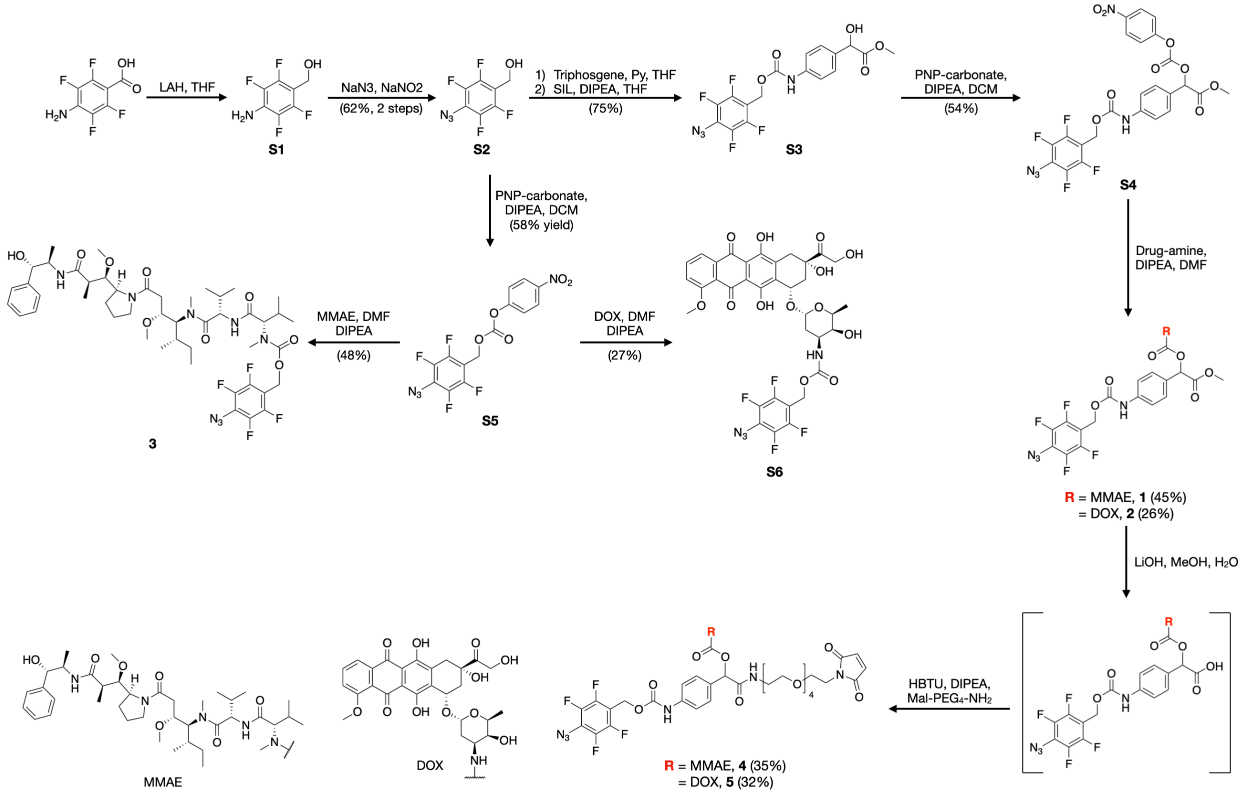


**Scheme S1.** Prodrug synthesis. Intermediates were synthesized according to prior literature.[9, 10]

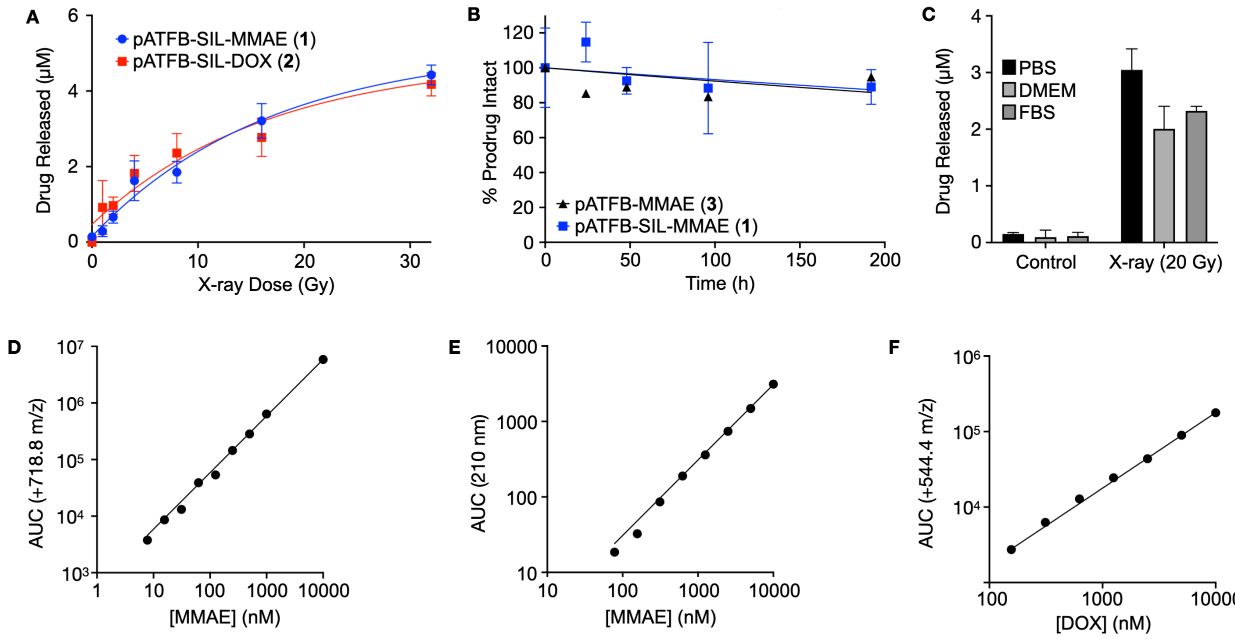


**Figure S1. *In vitro* prodrug release and stability.** A) Percentage of MMAE or DOX released from pATFB prodrugs **1** and **2** after X-ray irradiation. B) Percentage of initial prodrug remaining over time while incubating at 37°C (pH 7) in PBS. C) Active MMAE released from prodrug **1** in growth medium (DMEM), fetal bovine serum (FBS), or PBS, following X-ray irradiation. Data are n = 3, means ± s.e.m. Representative data for LC/MS (D) and HPLC (E) calibration curves for MMAE detection and LC/MS for DOX detection (F).

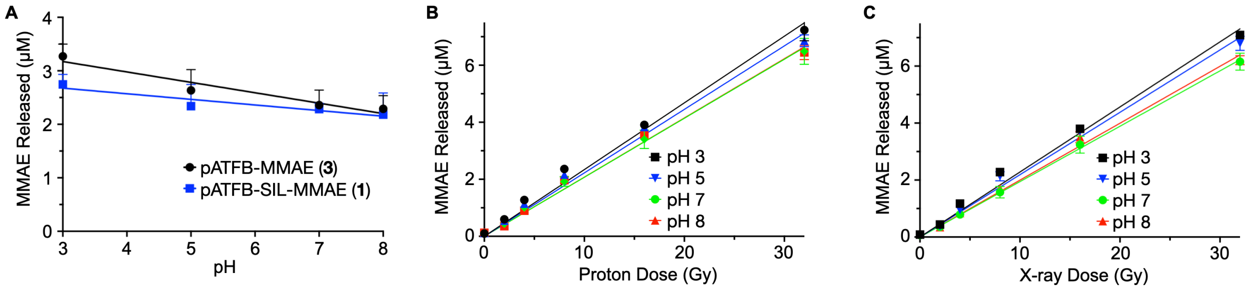


**Figure S2. *In vitro* prodrugs release across pH 3-8.** A) MMAE released from prodrugs **1** and **4** after X-ray irradiation (10 Gy) in solutions of varying pH. B-C) MMAE released from prodrug **1** after proton (B) or X-ray (C) irradiation in solutions of varying pH. All data are means ± s.e.m (n = 3).

A

| | [Protein] | [MMAE] | DOL |
|---|---|---|---|
| RABiT-MMAE | 1.2 ± 0.3 µM | 1.0 ± 0.3 µM | 0.83 |
| Rad-Act-mAb | 2.4 ± 0.4 µM | 9.2 ± 0.2 µM | 3.8 |

| | [Polymer] | [MMAE] | Drug:Polymer Ratio |
|---|---|---|---|
| Rad-Act-NP | ~1 mM | 95.2 ± 3.7µM | 0.095 |

B

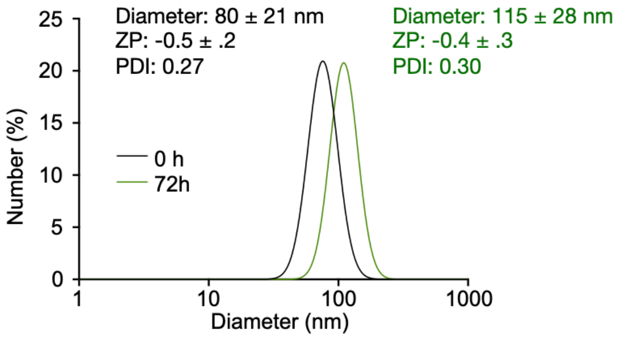


**Figure S3. Characterization of Rad-Act-mAb and Rad-Act-MMAE.** A) Concentrations of Msa/antibody and released MMAE measured by Nanodrop and LC/MS, respectively, to determine degree of labeling (molecules prodrug per protein). For polymer nanoparticle the ratio of MMAE to initial polymer concentration is reported. Data are means ± s.e.m (n = 3). B) Size and zeta potential measurements of Rad-Act-NP over 72 h incubating at 37°C (pH 7.4 in PBS). Data are means ± s.e.m (n = 3).

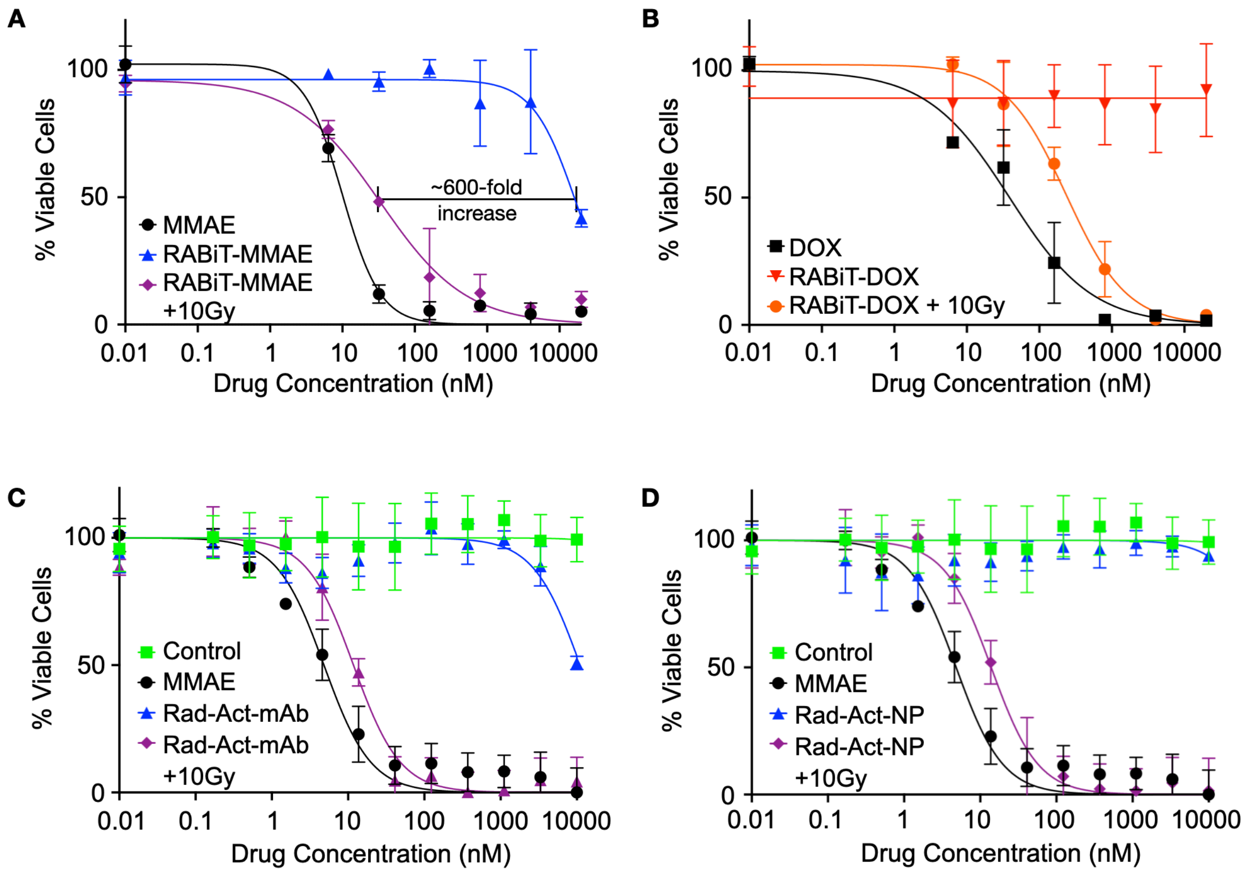


**Figure S4.** Cytotoxicity of non-irradiated or 10 Gy pre-irradiated RABiT-MMAE on MC38 cells, as measured after 72 hr incubation via a resazurin cytotoxicity/proliferation PrestoBlue assay. (A-B) Corresponds with Fig. 3c. (C-D) Cytotoxicity of Rad-Act-mAb (C, for assessing effects independent of antigen targeting) and Rad-Act-NP (D) in TBP3743 cell line with and without indirect irradiation. Data are means +/- s.e.m., n = 3.

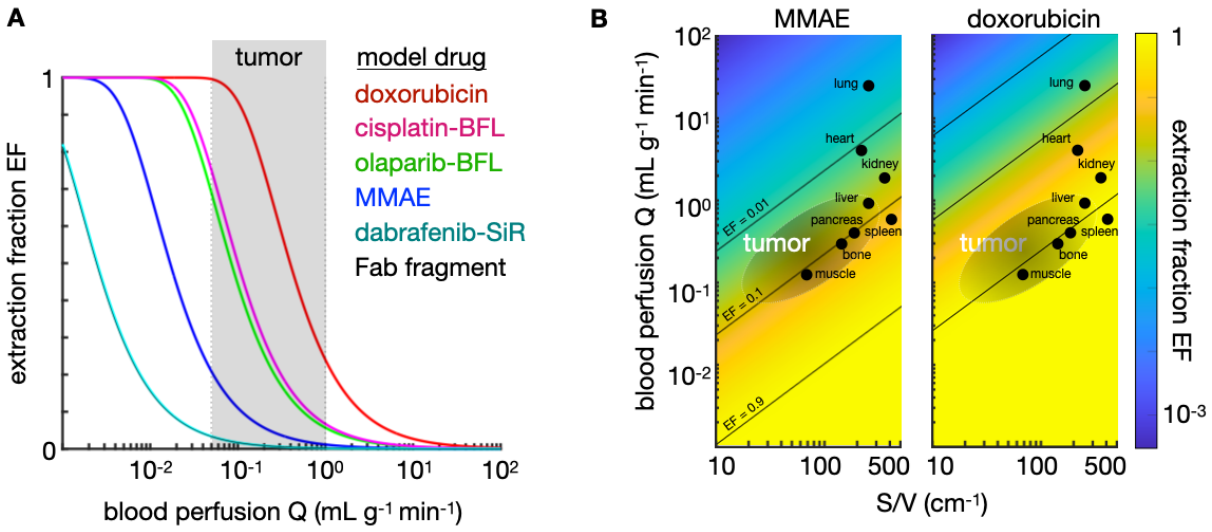


**Figure S5. Quantitative modeling drug extraction fraction as a function of tissue perfusion.** a) Extraction fraction (see *Equation 1*) as a function of tissue perfusion (mL blood per gram tissue per minute), based on vessel permeability values for different drugs. b) Extraction fraction as a function of blood perfusion and tissue vascularization (blood vessel surface area to tissue volume ratio). Tumor tissues show a heterogeneous range of perfusion and vascularization, shown roughly by gray shaded region. Heat map colors correspond to the calculated extraction fraction for MMAE (left) and doxorubicin (right). See Table S2 for values and their corresponding references. See ref.[11] for Q and S/V values.

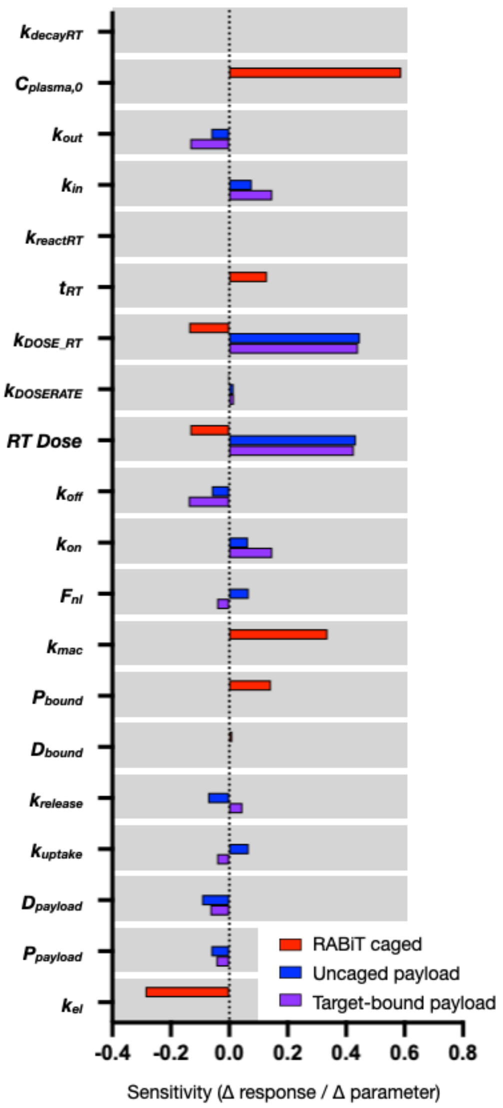


**Figure S6. Sensitivity analysis of computational PK/PD model.** Rate constants and parameters were adjusted +/- 10%, and average tumor values of caged RABiT, uncaged payload, and target-bound payload (microtubule-bound MMAE) were simulated with all other features held equal.

A

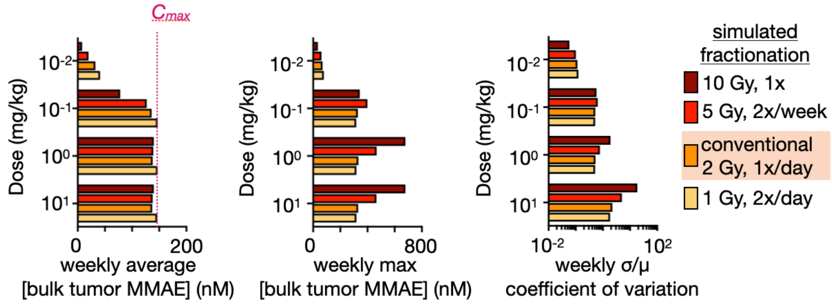


B

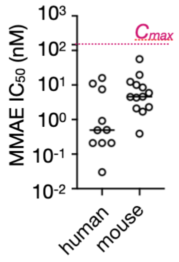


C

| Cell Line | MMAE IC50 (nM) Human | Mouse | Ref. |
|---|---|---|---|
| A549 | 0.5 | | [12] |
| HCT116 | 0.92 | | [12] |
| CAL27 | 0.21 | | [12] |
| D562 | 0.21 | | [12] |
| B16 | | 2.3 | [12] |
| LL2 | | 5.9 | [12] |
| MC38 | | 19.6 | [12] |
| MOC1 | | 8.5 | [12] |
| MOC2 | | 10.1 | [12] |
| 4MOSC1 | | 1.7 | [12] |
| 8505c | 0.21 | | [13] |
| TBP | | 4.6 | [13] |
| MOC2 | | 12.3 | [13] |
| MC38 | | 2.1 | [13] |
| iKras | | 4.3 | [13] |
| HT1080 | 0.03 | | [14] |
| CT26 | | 0.39 | [14] |
| ES2 | 0.49 | | [14] |
| 4T1 | | 4.5 | [14] |
| Panc1 | 11 | | [15] |
| MIAPaca2 | 7.5 | | [15] |
| BxPC3 | 16 | | [15] |
| B16 | | 56 | [15] |

**Figure S7. Comparing simulated tumor concentrations with cellular IC50 values.** A) Simulated average and maximum tumor concentrations of active MMAE in humans, repeated from Fig. 4h for reference and shown with $C_{max}$ to denote the highest average concentration. Coefficient of variation in active drug concentration over time is reported as standard deviation divided by the mean average. B-C) Simulated $C_{max}$ is higher than IC50 values reported in the literature for both human and mouse cell lines. The four published studies were used in this work since they reported concentrations to achieve 50% growth/cytotoxicity inhibition effect in both mouse and human cell lines for free MMAE.

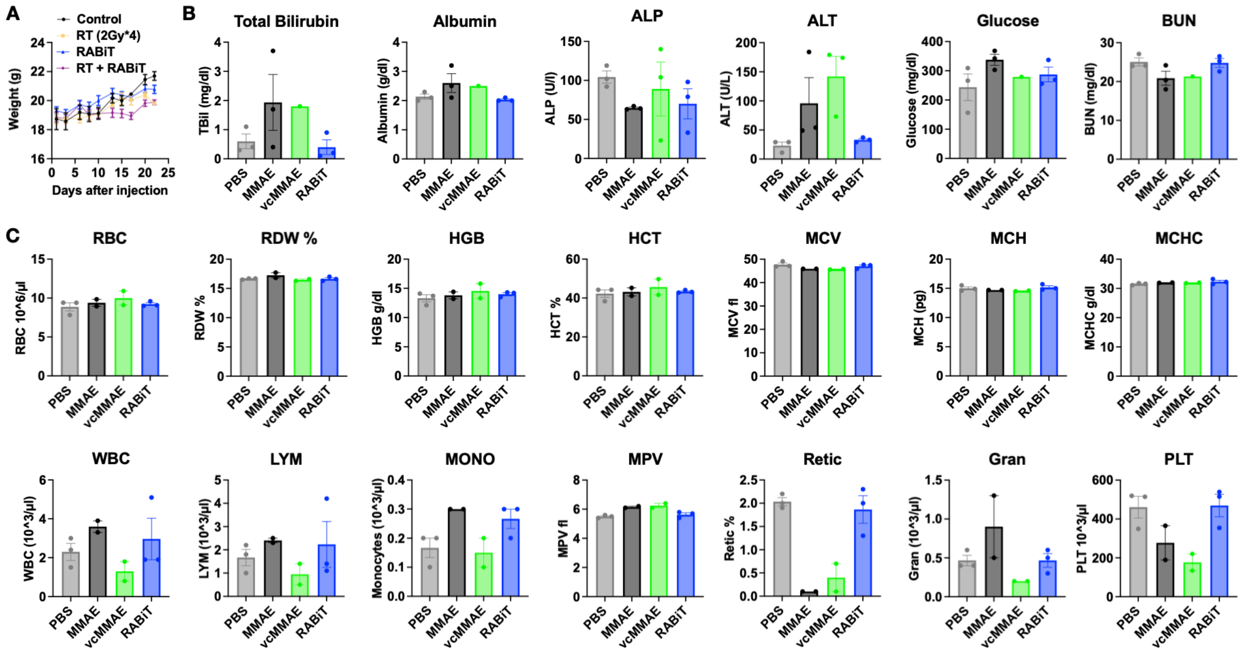


**Figure S8. RABiT toxicity. A)** Body weight measured during longitudinal efficacy assessment in ATC tumor-bearing mice, corresponding to Fig. 6a-b (n = 17). **B-C**) Serum and total blood samples from mice were collected on the second day post-treatment following treatment with either free MMAE, Alb-vc-MMAE, or RABiT-MMAE, and corresponding to Fig. 6c-d. **B**) Mouse serum total bilirubin (TBil), albumin, alkaline phosphatase (ALP), blood urea nitrogen (BUN), glucose, and alanine transaminase (ALT) were measured. **C**) Complete blood count measurement of white blood cells (WBC), lymphocytes (LYM), monocytes (MONO), granulocytes (Gran), platelets (PLT), red blood cells (RBC), hemoglobin (HGB), red blood cell distribution width (RDW), mean corpuscular hemoglobin (MCH), mean corpuscular hemoglobin concentration (MCHC), mean platelet volume (MPV), mean corpuscular volume (MCV), hematocrit (HCT), and reticulocytes (Retic). Data are means ± SEM, n=1-3 mice per group.

| ***Blood*** | | ***Concentration volume*** |
|---|---|---|
| $y_1$ plasma, c | $dy_1/dt = - k_{el}\, y_1$ | Plasma |
| | | |
| ***Effective diffusion transport*** | | |
| $y_2$ tumor interstitium, uncaged | $\partial y_2/\partial t = D_{eff,ub}\, (\partial^2 y_2/\partial r^2) + 2\, D_{eff,ub}\, r^{-1}\, (\partial y_2/\partial r)$ | Interstitium |
| $y_3$ tumor interstitium, caged | $\partial y_3/\partial t = D_{eff,ALB}\, (\partial^2 y_3/\partial r^2) + 2\, D_{eff,ALB}\, r^{-1}\, (\partial y_3/\partial r)$ | Interstitium |
| | | |
| ***Tumor reactions*** | | |
| $y_2$ tumor interstitium, uncaged | $dy_2/dt = \textit{transport} - [\, F_{nl}\, (k_{lu} y_2 - k_{lr} y_4) + \varepsilon_c\, (k_{out} y_7 - k_{in} y_2)\, ]\, \varepsilon_x^{-1} + k_{RT} y_3 y_8$ | Interstitium |
| $y_4$ tumor lipid uncaged | $dy_4/dt = k_{lu} y_2 - k_{lr} y_4$ | Tumor Lipid |
| $y_3$ tumor interstitium, caged | $dy_3/dt = \textit{transport} - k_{mac} y_3 - k_{RT} y_3 y_8$ | Interstitium |
| $y_4$ tumor intracellular caged | $dy_4/dt = \varepsilon_{x,c}\, \varepsilon_c^{-1}\, k_{mac} y_3 - k_{RT} y_4 y_9$ | Intracellular |
| $y_5$ tumor target binding sites | $dy_5/dt = - k_{on} y_5 y_7 + k_{off} y_6$ | Intracellular |
| $y_6$ tumor bound target | $dy_6/dt = k_{on} y_5 y_7 - k_{off} y_6$ | Intracellular |
| $y_7$ tumor intracellular uncaged | $dy_7/dt = k_{RT} y_4 y_9 - k_{on} y_5 y_7 + k_{off} y_6 - k_{out} y_7 + k_{in} y_2$ | Intracellular |
| $y_8$ interstitial RT reductants | $dy_8/dt = k_{DOSE\_RT}\, k_{DOSERATE}\, f_{RT}(\, k_{RT\_t\_on}\, ,\, k_{RT\_t\_off})\, \varepsilon_x - k_{RT} y_3 y_8 - k_{decay} y_8$ | Interstitium |
| $y_9$ intracellular RT reductants | $dy_9/dt = k_{DOSE\_RT}\, k_{DOSERATE}\, f_{RT}(\, k_{RT\_t\_on}\, ,\, k_{RT\_t\_off})\, \varepsilon_c - k_{RT} y_4 y_9 - k_{decay} y_9$ | Intracellular |
| $f_{RT}$ RT reductant generator | 1, if $t >= k_{RT\_t\_on}$ and $t <= k_{RT\_t\_off}$<br>$\min\{\, 1\, ,\, (1/\sqrt{2\pi}) e^{-(x-\mu_i)^2/2}\, \}$, $\mu_i = \{\, k_{RT\_t\_on}\, ,\, k_{RT\_t\_off}\, \}$, if $t < k_{RT\_t\_on}$ or $t > k_{RT\_t\_off}$ | |

**Table S1. Equations used in the computational PK/PD model.**

| Model parameter | Description | Value | Reference |
|---|---|---|---|
| $k_{el}$ | Circulating RABiT half-life | 0.0004 $min^{-1}$ | This study |
| $y_5$( t=0 ) | Target binding sites | 20 μM | Khera et al.[12] |
| $P_{payload}$ | Payload vessel permeability | 100 x $10^{-7}$ cm $s^{-1}$ | This study, Zhang et al.[13] |
| $P_{caged}$ | Caged RABiT vessel permeability | 2 x $10^{-7}$ cm $s^{-1}$ | Thurber & Weissleder[14] |
| $D_{eff, payload}$ | Effective interstitial diffusion, caged | 15 x $10^{-8}$ $cm^2$ $s^{-1}$ | Thurber & Weissleder[14] |
| $D_{eff, caged}$ | Effective interstitial diffusion, payload | 10 x $10^{-8}$ $cm^2$ $s^{-1}$ | Thurber & Weissleder[14] |
| $R_0$ | Tumor radius | 300 μm | |
| $F_{nl}$ | Lipid fraction | 0.07 | Portoukalian et al.[15], Holt et al.[16] |
| $k_{on}$ | Payload / target binding on-rate | 8333 $M^{-1}$ $s^{-1}$ | Khera et al.[12] |
| $k_{off}$ | Payload / target binding off-rate | 0.003 $s^{-1}$ | Khera et al.[12] |
| $k_{DOSERATE}$ | Rate of RT dose delivery | 0.15 Gy $min^{-1}$ | |
| $k_{DOSE\_RT}$ | RT dose delivery | 150 nM $Gy^{-1}$ | This study; likely underestimated for simulation tolerance |
| $k_{reactRT}$ | Reaction rate prodrug cleavage | $10^8$ $M^{-1}$ $s^{-1}$ | Likely underestimated for simulation tolerance |
| $k_{decayRT}$ | RT reactive species decay | 40 $min^{-1}$ | Underestimated for simulation tolerance |
| $k_{mac}$ | Tumor macropinocytosis rate | 0.001 $min^{-1}$ | Fit to %ID/g, this study |
| $k_{in}$ | Payload cellular influx rate | 1.4 x $10^{-3}$ $s^{-1}$ | Khera et al.[12] |
| $k_{out}$ | Payload cellular efflux rate | 6.9 x $10^{-4}$ $s^{-1}$ | Khera et al.[12] |
| $\varepsilon_x$ | Extracellular void fraction | 0.25 | Thurber & Weissleder[14], Vasalou et al.[17] |
| $\varepsilon_c$ | Fraction cell cytoplasm | 0.2 | 0.35 cytoplasm fraction x (1-extracellular volume) Moore et al.[18], Chang et al.[19] |
| $k_{on}$ | MT association rate | 8333 $M^{-1}$ $s^{-1}$ | Khera et al.[12] |
| $k_{off}$ | MT dissociation rate | 0.003 $s^{-1}$ | Khera et al.[12] |
| | | | |
| $F_{free, MMAE, hu}$ | Fraction free, MMAE, humans | 0.32 | Chang et al.[20] |
| $F_{free, MMAE, mu}$ | Fraction free, MMAE, mice | 0.75 | Chang et al.[20] |
| $P_{olaparib\text{-}BDP} * F_{free}$ | Eff. permeability, olaparib-BODIPY | 160 x $10^{-7}$ cm $s^{-1}$ | This study, Thurber et al.[21] |
| $P_{cisPT\text{-}BDP} * F_{free}$ | Eff. permeability, cispt-BODIPY | 190 x $10^{-7}$ cm $s^{-1}$ | This study, Miller et al.[22] |
| $P_{dab\text{-}SiR} * F_{free}$ | Eff. permeability, dab-SiR | 4.7 x $10^{-7}$ cm $s^{-1}$ | This study, Ng et al.[23] |
| $P_{fab\ frag}$ | Permeability, fab fragment | 4.6 x $10^{-7}$ cm $s^{-1}$ | Zhang et al.[13] |
| $P_{doxorubicin}$ | Permeability, doxorubicin | 3000 $cm^{-1}$ $s^{-1}$ | Thurber & Weissleder[14] |
| $HCT_{tumor\ cap}$ | Tumor capillary hematocrit | 0.18 | Thurber & Weissleder[14] |

## Table S2. Rate constants and parameters used in modeling.

# 3.Organic Synthesis

## 3.1.General Information

***Health and safety concern:***

Sodium azide is an acutely toxic agent and should be handled with care to avoid inhalation and/or contact with the skin. Organic azides are potentially explosive and should be handled with care when in solid form. The authors recommend keeping the azides in this synthesis below 30°C at all times, particularly when drying by rotary evaporation.

*Unless stated otherwise, all materials were used as received from commercial sources.* Triphosgene, N,N-diisopropylethylamine, tetrahydrofuran, N,N-dimethylformamide, bis(4-nitrophenyl) carbonate, ethanolamine, lithium hydroxide, and HBTU were purchased from Sigma Aldrich (St. Louis, MO, USA). Hydrochloric acid (HCl), methanol, dichloromethane, and acetonitrile were purchased from VWR International (Radnor, PA, USA), while monomethyl auristatin E and doxorubicin hydrochloride were purchased from MedChem Express (Monmouth Junction, NJ, USA). Maleimide-PEG4-amine trifluoroacetic acid salt was purchased from BroadPharm (San Diego, CA, USA) and 4-amino-2,3,5,6-benzyl alcohol was purchased from Fisher Scientific (Hampton, NH, USA). MeOD-$d_4$, and $CDCl_3$ were purchased from Cambridge Isotope Laboratories (Tewksbury, MA, USA). Reaction mixtures were purified using a Biotage SNAP Bio C18 300 Å 10 g on a Biotage Isolera with a gradient composed of water (0.1% formic acid) and acetonitrile (0.1% formic acid) for reversed-phase chromatography. $^{1}H$, and $^{13}C$ NMR spectra were recorded on a Bruker AC-400 MHz spectrometer. High performance liquid chromatography-mass spectrometry analysis (HPLC-MS, LC/MS) was performed on a Waters instrument equipped with a Waters 2424 ELS Detector, Waters 2998 UV-Vis Diode array Detector, Waters 2475 Multi-wavelength Fluorescence Detector, and a Waters 3100 Mass Detector. Separations employed an HPLC-grade water/acetonitrile solvent gradient. Columns: XTerra MS C18 Column, 125., 5 µm, 4.6 mm X 50 mm column. HRMS analysis was carried out on a Thermo Scientific Dionex UltiMate 3000 UHPLC coupled to a Thermo Q Exactive Plus mass spectrometer system (Thermo Fisher Scientific Inc, Waltham, MA) equipped with an HESI-II electrospray ionization (ESI) source. Data were acquired with Chromeleon Xpress software for UHPLC and Thermo Xcalibur software version 3.0.63 for mass spectrometry, and processed with Thermo Xcalibur Qual Browser software version 4.0.27.19.

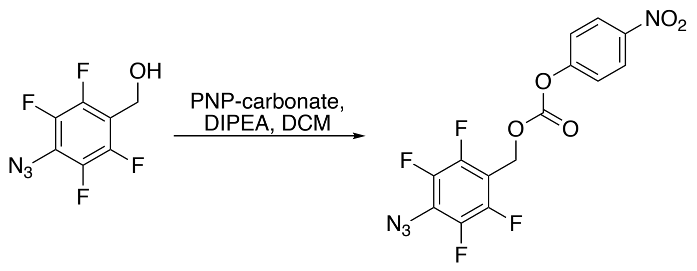


**Synthesis of pATFB-PNP (S5).** 4-azido-2,3,5,6-fluorobenzyl alcohol (100.2 mg, 0.452 mmol) and bis-4-nitrophenyl carbonate (138 mg, 0.452 mmol) were dissolved in DCM (10 mL). DIPEA (80 µL, 0.454 mmol) was added and the mixture was shaken at room temperature for 18 hrs. The crude mixture was evaporated and redissolved in toluene, then purified using a gradient of 0-25% ethyl acetate in hexanes, followed by a flush using 10% MeOH in DCM. The desired compound was isolated as a pale-yellow solid (101.7 mg, 58% yield).

**$^1$H NMR** (400 MHz, $CDCl_3$): δ 8.28 (d, *J* = 7.1 Hz, 2H), 7.40 (d, *J* = 9.2 Hz, 2H), 5.39 (s, 2H).

**$^{13}$C NMR** (101 MHz, $CDCl_3$): δ 155.3, 152.1, 147.1, 145.7, 144.6, 139.3, 125.5, 121.8, 108.2, 57.8.

**$^{19}$F NMR** (376 MHz, $CDCl_3$): δ -142.2, -151.4.

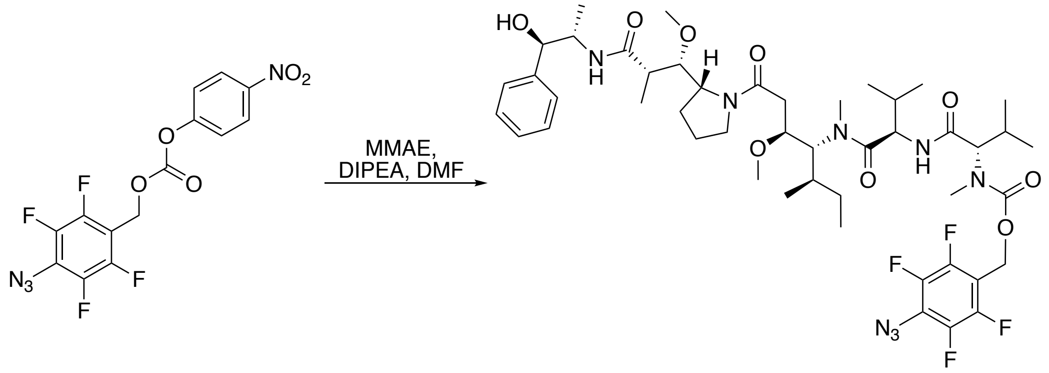


**Synthesis of pATFB-MMAE (3)**. MMAE (50 mg, 69.6 µmol) and pATFB-PNP (40.3 mg, 104 µmol) were dissolved in dry DMSO (2.5 mL). DIPEA (18 µL, 104 µmol) was added and the mixture was shaken at 30 °C for 19 hrs. A further 1.5 eq (18 µL) of DIPEA was added and the reaction was left stirring for a further 20 hrs. The reaction mixture was then treated with ethanolamine (20 µL, 331 µmol) to quench unreacted pATFB-PNP. This mixture was stirred for 1 hr, then found to be free of PNP carbonate (by LC/MS) and loaded directly onto a reverse-phase column and purified using a gradient of 5-100% acetonitrile in water (0.1% formic acid). The product was purified as a white solid (32.5 mg, 48% yield).

**$^1$H NMR** (400 MHz, $CDCl_3$): δ 7.66 (brs, 2H), 7.32 (s, 3H), 6.65 (s, 1H), 5.29 (q, *J* = 12.1 Hz, 1H), 5.17 (t, *J* = 9.4 Hz, 1H), 5.08-4.85 (m, 1H) 4.80-4.60 (m, 1H), 4.27 (s, 1H), 4.17-4.05 (m, 2H), 3.79 (s, 1H), 3.61-3.31 (m, 5H), 3.19-3.01 (m, 2H), 2.91 (s, 1H), 2.82 (s, 1H), 2.71 (d, *J* = 7.3 Hz, 2H), 2.63-2.53 (m, 1H), 2.21-1.90 (m, 4H), 1.24 (s, 4H), 1.10-0.91 (m, 7H), 0.90-0.75 (m, 10H).

**$^{13}$C NMR** (101 MHz, $CDCl_3$): δ175.6, 171.7, 170.0, 159.4, 159.0, 158.6, 156.6, 155.4, 147.0, 144.5, 143.7, 141.7, 140.9, 139.4, 128.3, 127.6, 126.5, 124.9, 120.8, 119.3, 116.4, 114.8, 113.6, 110.4, 81.7, 78.9, 75.6, 65.3, 61.2, 58.3, 57.7, 57.1, 55.1, 54.4, 52.5, 44.4, 40.1, 36.5, 33.2, 31.1, 30.0, 29.8, 26.8, 26.2, 25.8, 25.0, 19.4, 18.6, 17.7, 16.1, 14.7, 13.8, 10.8.

**$^{19}$F NMR** (376 MHz, $CDCl_3$): δ -142.6, -151.8.

**MS**: m/z calculated for $C_{47}H_{69}F_4N_8O_9^+$ [M+H$^+$] 964.5045, found 965.5118.

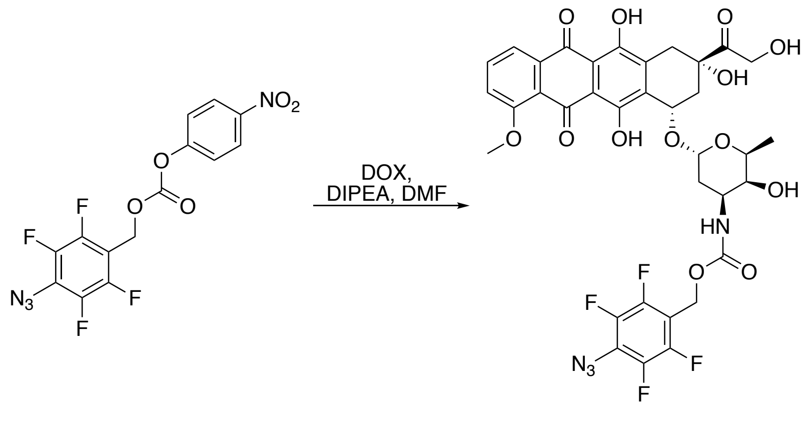


**Synthesis of pATFB-DOX (S6)**. pATFB-PNP (98.2 mg, 165 μmol) and doxorubicin monohydrate (50 mg, 89.0 μmol) were dissolved in dry DMF (1 mL) and DIPEA (25 μL, 143.5 μmol) added dropwise. After 1 hr shaking at room temperature, LC/MS showed no remaining doxorubicin, so ethanolamine (25 μL, 409.3 μmol) was added and shaking continued for 1 hr to react with any remaining pATFB-PNP. The mixture was then loaded directly onto a reverse-phase column and purified with a gradient of 5-100% acetonitrile in water (0.1% formic acid). The product was isolated as a red solid (17.8 mg, 27% yield).

**$^1$H NMR** (400 MHz, $CDCl_3$): δ 13.99 (s, 1H), 13.26 (s, 1H), 8.26 (t, *J* = 9.4 Hz, 1H), 8.04 (s, 2H), 7.81 (t, *J* = 8.4 Hz, 1H), 7.42 (d, *J* = 8.4 Hz, 1H), 5.22 (s, 1H), 5.35-5.17 (m, 4H), 5.13 (s, 2H), 4.77 (s, 2H), 4.16 (d, *J* = ,6.9 Hz, 1H), 4.10 (s, 3H), 3.88 (brs, 1H), 3.74 (s, 1H), 3.68 (s, 2H), 3.39 (t, *J* = 6.48 Hz, 1H), 3.29 (d, *J* = 18.0 Hz, 1H), 2.99 (s, 2H), 2.91 (s, 2H), 2.34 (d, *J* = 14.6 Hz, 2H), 2.18 (dd, *J* = 14.66, 4.3 Hz, 2H), 2.03 (s, 1H), 1.88 (dd, *J* = 13.1, 3.9 Hz, 1H), 1.80 (td, *J* = 13.4, 4.3 Hz, 1H), 1.54 (d, *J* = 6.6 Hz, 1H), 1.46 (d, *J* = 6.6 Hz, 1H), 1.30 (d, *J* = 7.6 Hz, 4H).

**$^{13}$C NMR** (101 MHz, $CDCl_3$): δ 213.9, 187.3, 186.8, 171.9, 156.3, 155.8, 154.9, 144.4, 136.0, 125.6, 120.9, 120.0, 118.6, 111.7, 100.8, 69.9, 69.6, 67.3, 65.7, 56.8, 54.0, 47.3, 35.8, 34.1, 30.3, 17.5, 15.9.

**$^{19}$F NMR** (376 MHz, $CDCl_3$): δ -142.33, -151.76.

**MS**: m/z calculated for $C_{35}H_{29}F_4N_4O_{13}^-$ [M-H$^-$] 789.17, found 789.72.

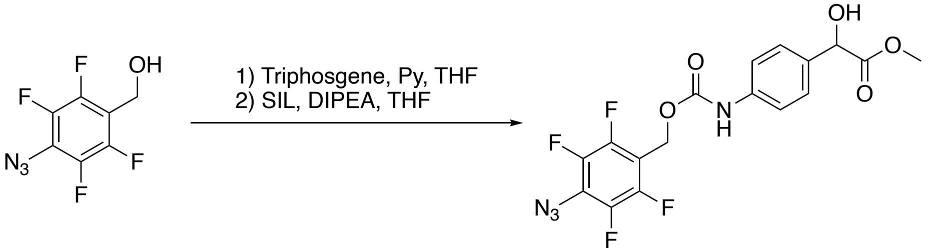


**Synthesis of pATFB-SIL (S3)**. 4-azido-2,3,5,6-tetrafluorobenzyl alcohol (600 mg, 2.71 mmol) was dissolved in THF (10 mL) and cooled to 0 °C before triphosgene (251 mg, 0.846 mmol) and pyridine (300 µL, 3.72 mmol) were added. This mixture was allowed to stirred for 2 hours, then SIL (28.3 mg, 156.4 µmol) and DIPEA (600 µL, 3.44 mmol) were added. The reaction mixture was stirred for a further hour at 0 °C , then diluted with ethyl acetate and washed successively with 1M HCl and brine before the organic phase was evaporated by rotary evaporation and redissolved in toluene (5 mL). The product was purified by loading the crude mixture onto at normal phase column and running a gradient of 0-20% EtOAc in Hexanes. The product was isolated as a white solid (874.3 mg, 75% yield).

**$^{1}$H NMR** (400 MHz, $CDCl_3$): δ 7.36 (s, 4H), 6.70 (s, 1H), 5.28 (s, 2H), 5.14 (s, 1H), 3.75 (s, 1H).

**$^{13}$C NMR** (101 MHz, $CDCl_3$): δ 174.1, 152.4, 137.5, 133.7, 127.5, 118.9, 109.8, 72.4, 54.2, 53.1.

**MS**: m/z calculated for $C_{17}H_{11}F_4N_4O_9^-$ [M - H$^-$] 427.07 found 427.37.

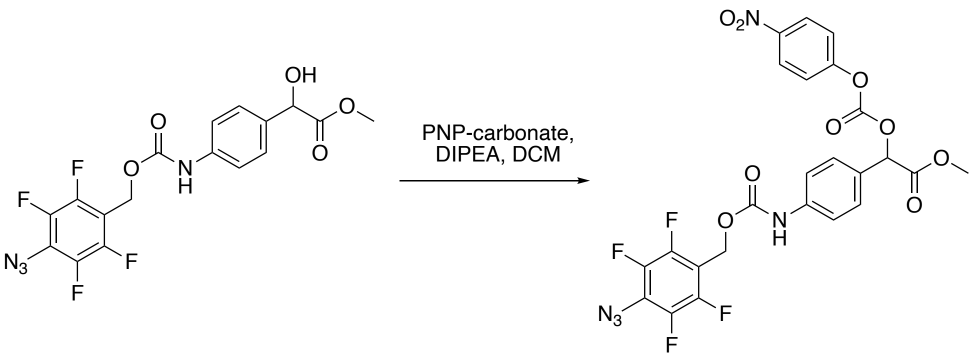


**Synthesis of pATFB-SIL-PNP (S4)**. pATFB-SIL (17.8 mg, 41.6 µmol) and bis(4-nitrophenyl) carbonate (13.0 mg, 42.7 µmol) were dissolved in DCM (1 mL). DIPEA (10 µL, 57.4 µmol) was added and the reaction was stirred at 45 °C for 20 hrs. The reaction mixture was then diluted with DCM (50 mL) and washed sequentially with 1M HCl, water, and brine (50 mL each). The organic phase was dried with magnesium sulfate, evaporated, and purified by normal-phase chromatography using a gradient of 0-50% EtOAc in hexanes. The product was obtained as a white solid (13.3 mg, 54% yield).

**$^1$H NMR** (400 MHz, $CDCl_3$): δ 8.27 (d, $J$ = 7.0 Hz, 2H), 7.48-7.40 (m, 6H), 7.17 (s, 1H), 5.90 (s, 1H), 5.28 (s, 2H), 3.76 (s, 3H).

**$^{13}$C NMR** (101 MHz, $CDCl_3$): δ168.5, 155.4, 151.9, 145.7, 139.2, 128.8, 127.6, 125.5, 121.9, 119.1, 77.8, 53.2, 41.0.

**$^{19}$F NMR** (376 MHz, $CDCl_3$): δ -142.1, -151.5.

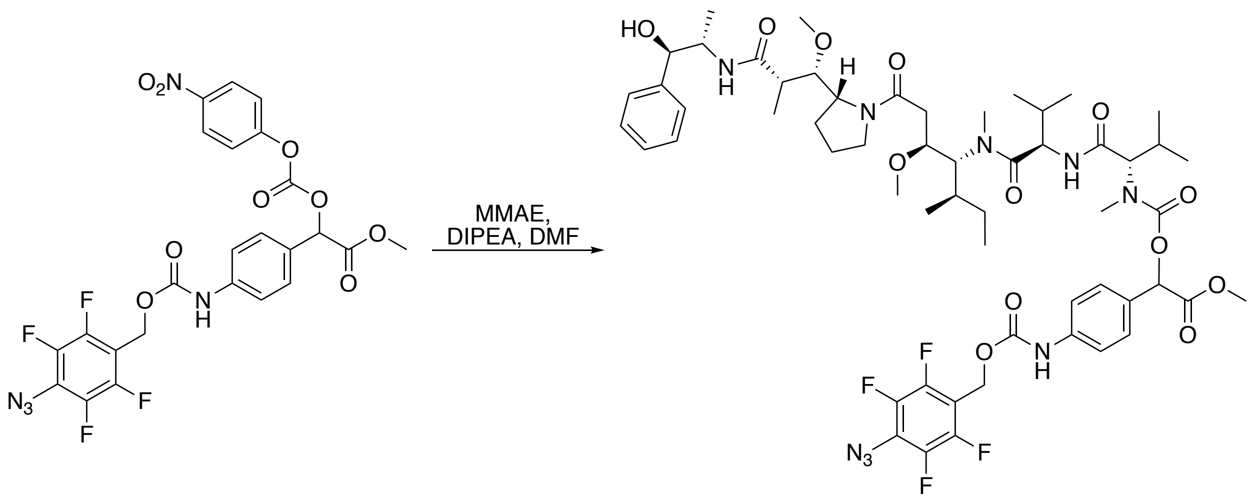


**Synthesis of pATFB-SIL-MMAE (1)**. MMAE (20.6 mg, 28.7 µmol) was dissolved in dry DMF (1 mL) and pATFB-SIL-PNP (15.4 mg, 26.0 µmol) was added. Once everything had dissolved, DIPEA (10 µL, 57.4 µmol) was added and the reaction was stirred at 30 °C for 16 hrs. The reaction mixture was then loaded directly onto a reverse phase column and purified using a gradient of 5-100% acetonitrile (0.1% formic acid) in water (0.1% formic acid) to provide the product as a white solid (13.7 mg, 45% yield).

(Mixture of diastereomers and rotamers; major peaks reported)

**$^1$H NMR** (400 MHz, $CDCl_3$): δ 8.18 (d, *J* = 8.9 Hz, 2H), 8.12 (d, *J* = 8.8 Hz, 1H), 7.62-7.33 (m, 5H), 6.97 (s, 3H), 5.28 (s, 1H), 5.02 (s, 1H), 4.70-4.63 (m, 1H), 4.51 (s, 1H), 4.26 (s, 1H), 3.76 (s, 6H), 3.40 (s, 1H), 3.31 (s, 2H), 3.02 (s, 1H), 2.70 (s, 1H), 2.44 (s, 1H), 2.10-1.75 (m, 3H), 1.24 (s, 1H), 1.05-0.78 (m, 9H).

**$^{13}$C NMR** (101 MHz, $CDCl_3$): δ 169.4, 168.9, 162.7, 162.0, 161.7, 149.2, 146.7, 144.2, 143.5, 142.6, 142.4, 141.7, 141.2, 139.2, 129.7, 129.4, 129.3, 128.9,128.5 128.4, 128.1, 128.0, 127.3, 126.3, 126.3, 126.1, 123.8, 120.1, 116.5, 115.9, 115.5, 115.4, 112.0, 78.4, 78.0, 72.2, 58.4, 58.0, 53.5, 53.4, 53.1, 43.7, 35.5.

**$^{19}$F NMR** (376 MHz, $CDCl_3$): δ -142.2, -151.6.

**MS**: m/z calculated for $C_{57}H_{77}F_4N_9O_{13}^+$ [M+H+] 1171.56, found 1172.97

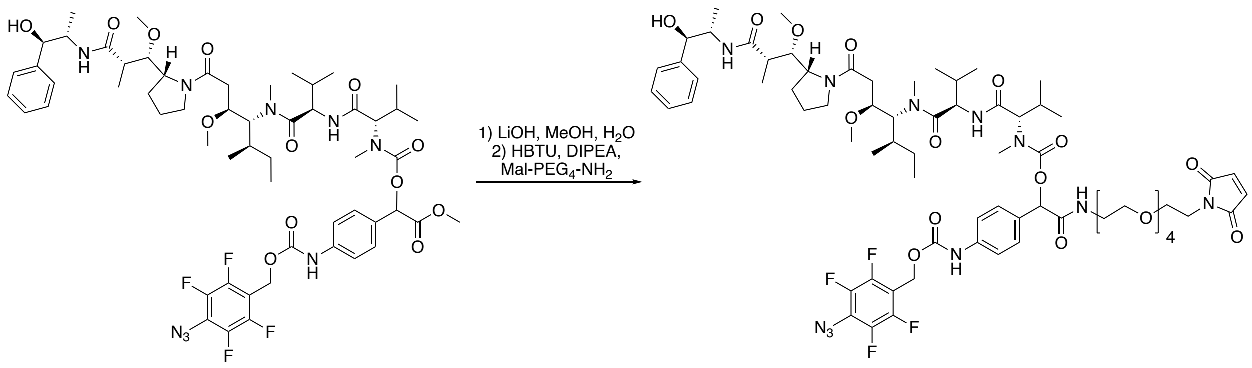


**Synthesis of pATFB-SIL-Mal-MMAE (4)**. pATFB-SIL-MMAE (28 mg, 23.9 µmol) was dissolved in MeOH (5 mL) and 0.5M LiOH (0.2 mL) was added. The mixture was stirred for 25 minutes, then analyzed by LC-MS. After it was determined that most of the material had formed the hydrolysis intermediate, the mixture was quenched by addition of acidic Amberlyst resin (~1 g). The suspension containing the resin was filtered and washed with methanol, and the solvent was removed via rotary evaporation. The resulting residue was dissolved in dry DMF (2 mL) and Mal-$PEG_4$-amine TFA salt (10 mg, 31.6 µmol), HBTU (20.2 mg, 53.2 µmol) and DIPEA (15 µL, 86.1 µmol) were added. This mixture was then stirred at room temperature for 16 hrs, then added directly to a reverse phase column and purified using a gradient of 5-100% acetonitrile in water (0.1% formic acid). The desired product was obtained as a white solid (12.2 mg, 35% yield).

(Mixture of diastereomers and rotamers; major peaks reported)

**$^{1}$H NMR** (400 MHz, $CDCl_3$): δ 7.52 (s, 1H), 7.37-7.30 (m, 8H), 7.04 (s, 2H), 6.69 (s, 1H), 5.32-5.15 (m, 3H), 4.95 (s, 1H), 4.26 (s, 1H), 4.16-4.08 (m, 4H), 3.71 (s, 2H), 3.68-3.50 (m, 14H), 3.41 (s, 4H), 3.31 (s, 3H), 3.10-2.85 (m, 5H), 2.80 (d, $J$ = 7.6 Hz, 1H), 2.5-2.3 (m, 2H), 2.24 (s, 1H), 2.02 (s, 2H), 1.85 (s, 2H), 1.11 (s, 1H), 1.03-0.78 (m, 24H).

**$^{19}$F NMR** (376 MHz, $CDCl_3$): δ -144.6, -154.0.

**MS**: m/z calculated for $C_{70}H_{98}F_4N_{11}O_{18}^+$ [M+H$^+$] 1456.7022, found 1457.16.

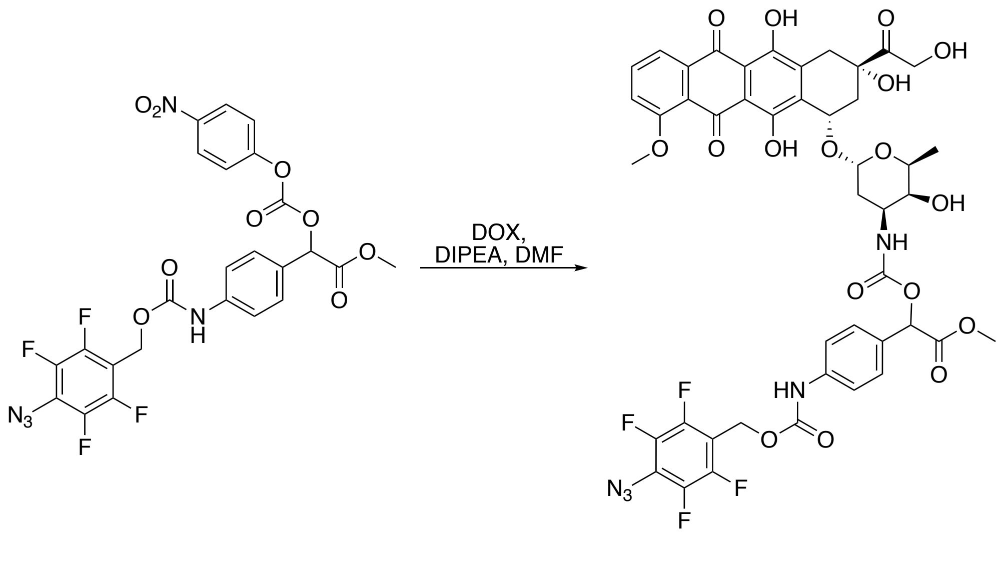


**Synthesis of pATFB-SIL-DOX (2)**. Doxorubicin (45 mg, 82.6 μmol) and pATFB-SIL-PNP (35 mg, 59.0 μmol) were dissolved in dry DMF (4 mL) and DIPEA (20 μL, 115.0 μmol) was added. The mixture was then shaken for 18 hrs on a rotary shaker at room temperature. Ethanolamine (20 μL, 327 μmol) was added to react with any remaining pATFB-SIL-PNP and the mixtures were incubated for an hour at room temperature before they were diluted with DCM (50 mL each) and washed with 1M HCl (50 mL each). The organic phase was then dried over magnesium sulfate and evaporated. Both crude residues were then dissolved in DMSO (1 mL), loaded onto a reverse phase column, and purified using a gradient of 5-100% acetonitrile (0.1% formic acid). Fractions containing the desired product were evaporated to provide the product as a red solid (21.4 mg, 26% yield).

(Mixture of diastereomers and rotamers; major peaks reported)

**$^{1}$H NMR** (400 MHz, $CDCl_3$): δ 13.90 (d, *J* = 8.1 Hz, 1H), 13.16 (dd, *J* = 9.3, 3.8 Hz, 1H), 8.03 (s, 1H), 7.97 (dd, *J* = 7.7, 4.7 Hz, 1H), 7.75 (t, *J* = 7.8 Hz, 1H), 7.39-7.30 (m, 4H), 7.24-20 (m, 1H), 5.74 (t, *J* = 8.7 Hz, 1H), 5.65-5.55 (m, 1H), 5.47 (s, 1H), 5.23 (t, *J* = 7.2 Hz, 3H), 4.76 (s, 1H), 4.74 (s, 1H), 4.32 (d, *J* = 13.8 Hz, 1H), 4.03 (s, 4H), 3.86-3.78 (m, 1H), 3.69-3.61 (m, 2H), 3.21 (dd, *J* = 18.7, 5.8 Hz, 1H), 2.95 (d, *J* = 18.3 Hz, 1H), 2.45-2.29 (m, 1H), 2.14 (t, *J* = 14.7 Hz, 1H), 2.00 (s, 1H), 1.90-1.80 (m, 2H), 1.28 (dd, *J* = 13.0, 6.5 Hz, 3H), 1.20-1.09 (m, 1H).

**$^{13}$C NMR** (101 MHz, $CDCl_3$): δ 213.7, 186.6, 162.9, 161.0, 156.2, 155.6, 135.8, 133.5, 128.5, 127.4, 127.2, 119.8, 118.5, 111.5, 100.6, 65.5, 56.6, 54.11, 47.3, 35.7, 33.8, 29.9, 16.8.

**$^{19}$F NMR** (376 MHz, $CDCl_3$): δ -142.2, -151.6.

**MS**: m/z calculated for $C_{45}H_{39}F_4N_5O_{17}^-$ [M-H$^-$] 996.2204, found 996.41.

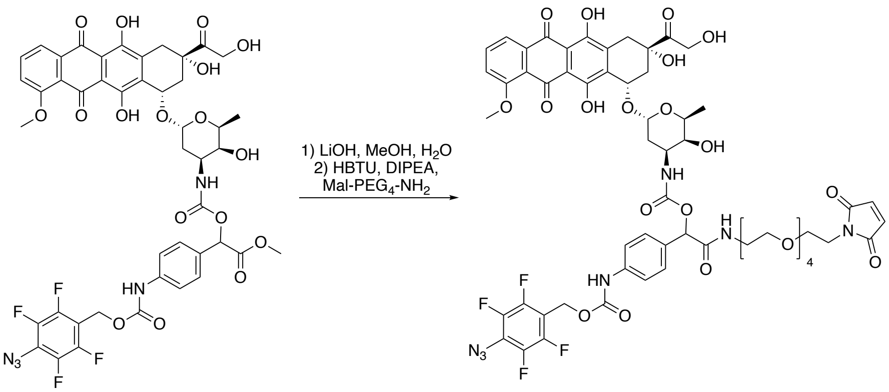

**Synthesis of pATFB-SIL-Mal-DOX (5)**. pATFB-SIL-DOX (24 mg, 24.0 μmol) was dissolved in MeOH (5 mL) and 0.5M LiOH (0.2 mL) was added. The mixture was stirred for 25 minutes, then analyzed by LC-MS. After it was determined that most of the material had formed the hydrolysis intermediate, the mixture was quenched by addition of acidic Amberlyst resin (~1 g). The suspension containing the resin was filtered and washed with methanol, and the solvent was removed via rotary evaporation. The resulting residue was dissolved in dry DMF (2 mL) and Mal-$PEG_4$-amine TFA salt (10 mg, 31.6 μmol), HBTU (20.3 mg, 53.5 μmol) and DIPEA (15 μL, 86.1 μmol) were added. This mixture was then stirred at room temperature for 16 hrs, then added directly to a reverse phase column and purified using a gradient of 5-100% acetonitrile in water (0.1% formic acid). The desired product was obtained as a red solid (10.0 mg, 32% yield).

(Mixture of diastereomers and rotamers; major peaks reported)

**$^{1}$H NMR** (400 MHz, DMSO-*d*$_6$): δ 14.03 (s, 1H), 13.26 (s, 1H), 9.92 (d, *J* = 15.3 Hz, 1H), 8.13 (s, 1H), 7.96-7.85 (m, 4H), 7.63 (s, 2H), 7.43-7.28 (m, 5H), 7.01 (s, 2H), 5.54 (s, 1H), 5.24 (s, 2H), 4.94 (s, 1H), 4.78-4.70 (m, 1H), 4.15-4.10 (m, 1H), 3.97 (s, 4H), 3.55-3.40 (m, 30H), 3.17-3.10 (m, 4H), 2.24 (d, *J* = 11.3 Hz, 1H), 2.11 (d, *J* = 13.9 Hz, 1.83 (s, 1H), 1.47 (s, 1H), 1.23 (d, *J* = 6.7 Hz, 12H), 1.12 (d, *J* = 7.1 Hz, 3H).

**$^{19}$F NMR** (376 MHz, DMSO-*d*$_6$): δ -69.2, -71.1, -73.4.

**MS**: m/z calculated for $C_{45}H_{39}F_4N_5O_{17}^-$ [M-H$^-$] 1281.36, found 1281.87.

## 4. Characterization

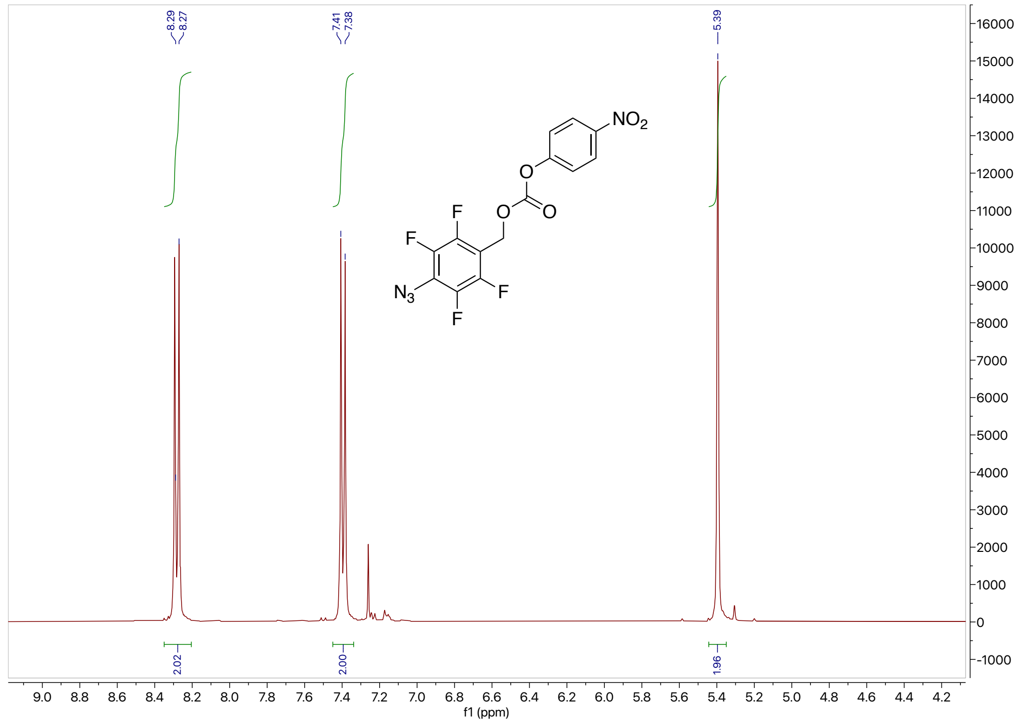


¹H NMR of pATFB-PNP in $CDCl_3$.

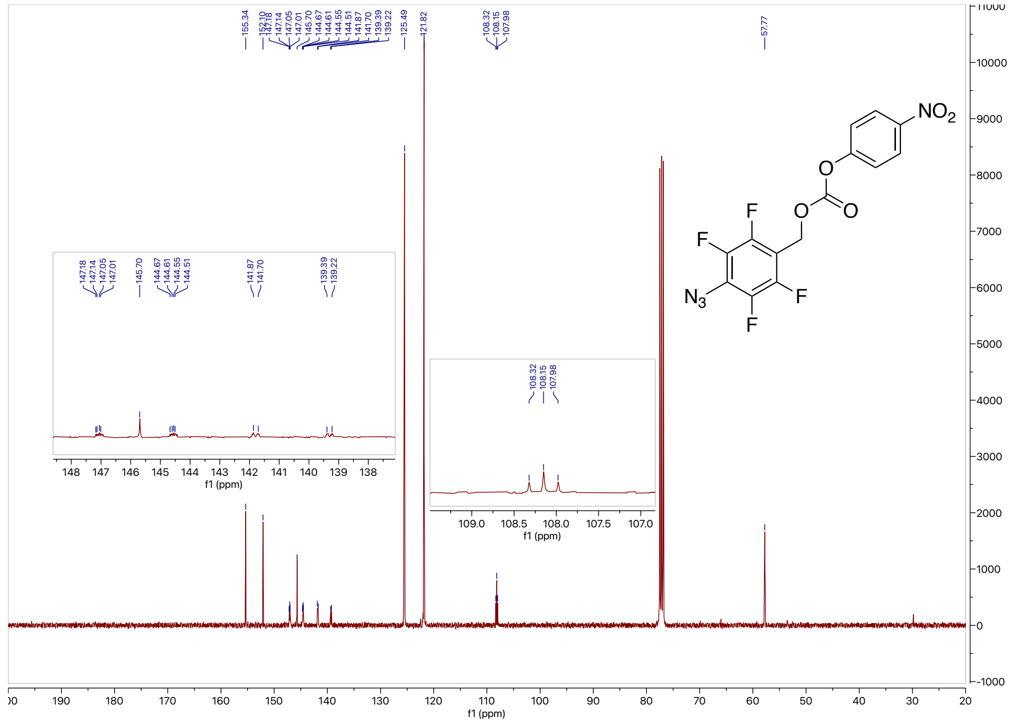


¹³C NMR of pATFB-PNP in $CDCl_3$.

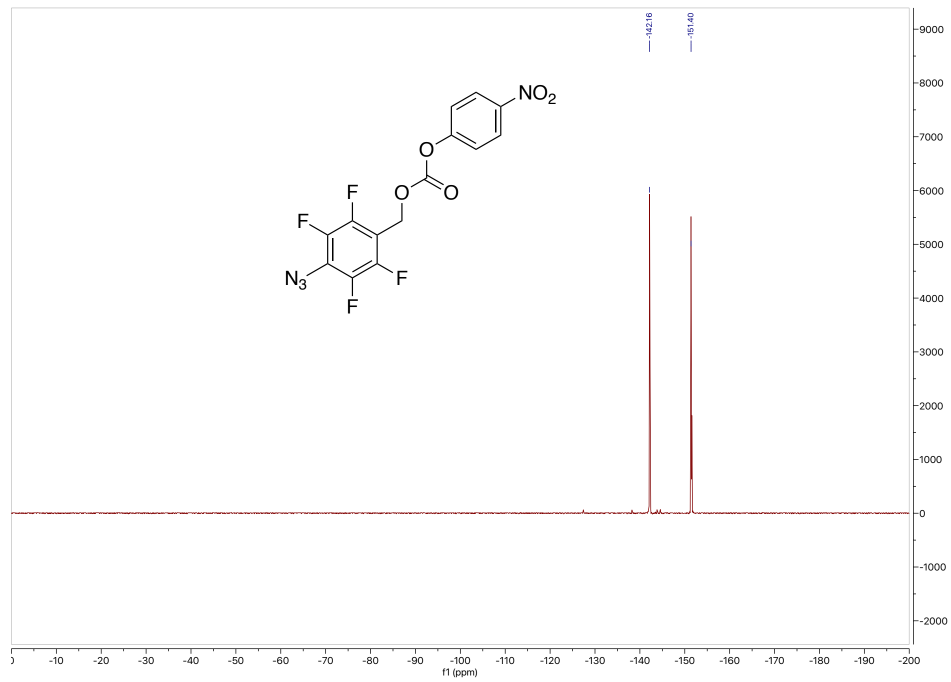


$^{19}$F NMR of pATFB-PNP in $CDCl_3$.

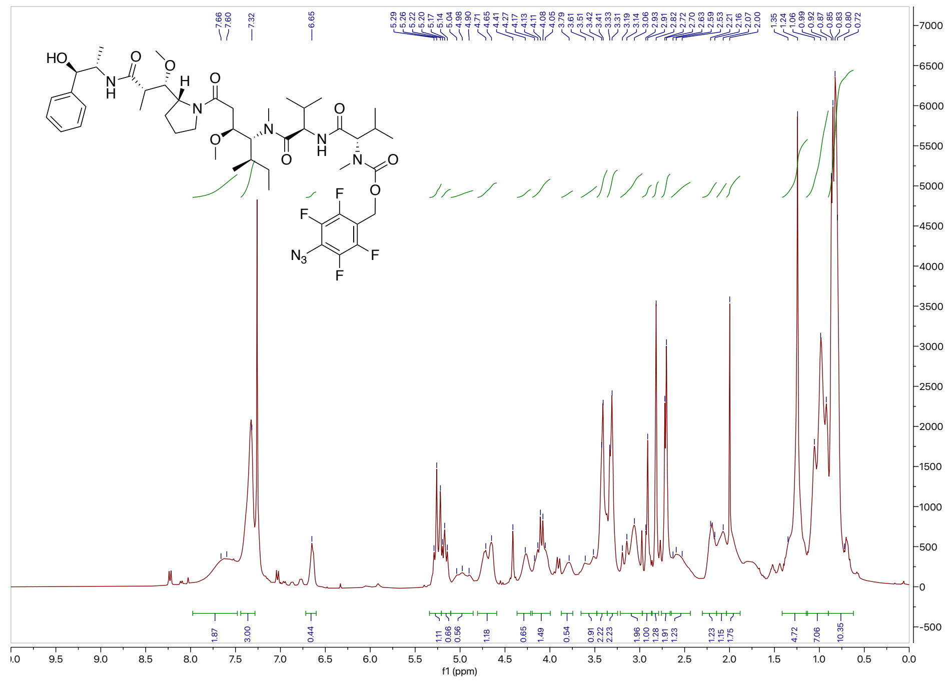


$^{1}$H NMR of pATFB-MMAE in $CDCl_3$.

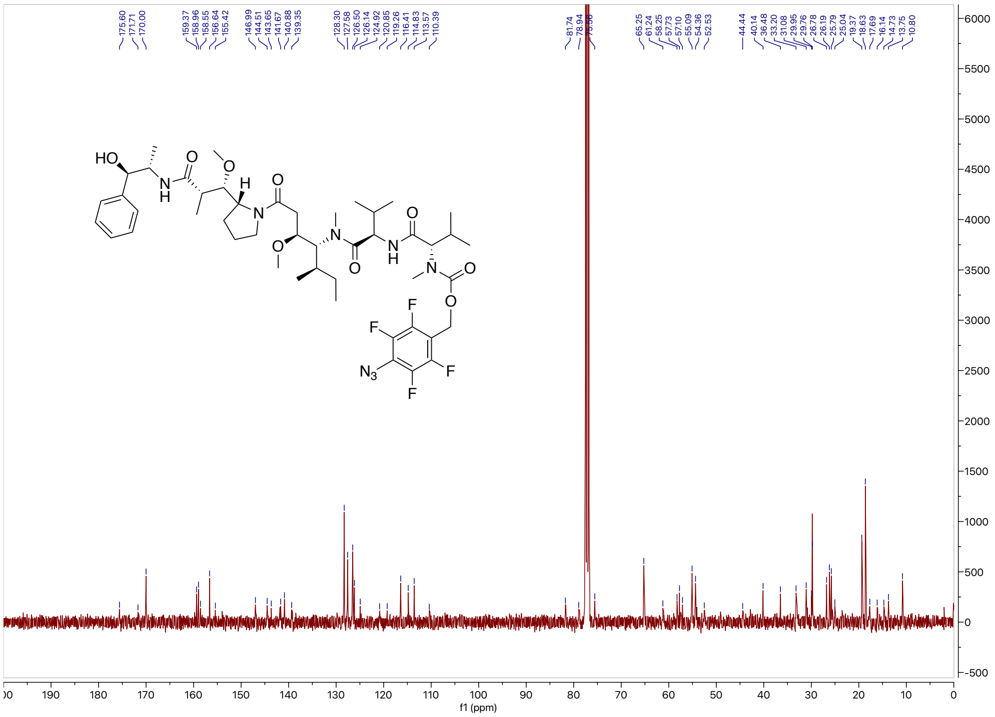


$^{13}C$ NMR of pATFB-MMAE in $CDCl_3$.

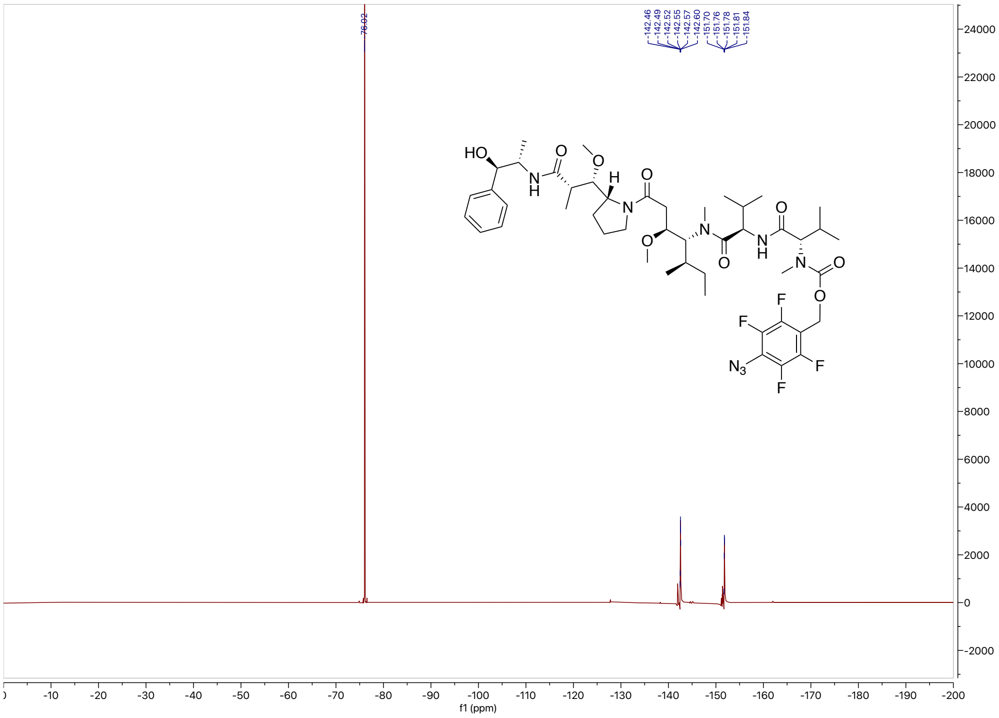


$^{19}F$ NMR of pATFB-MMAE in $CDCl_3$.

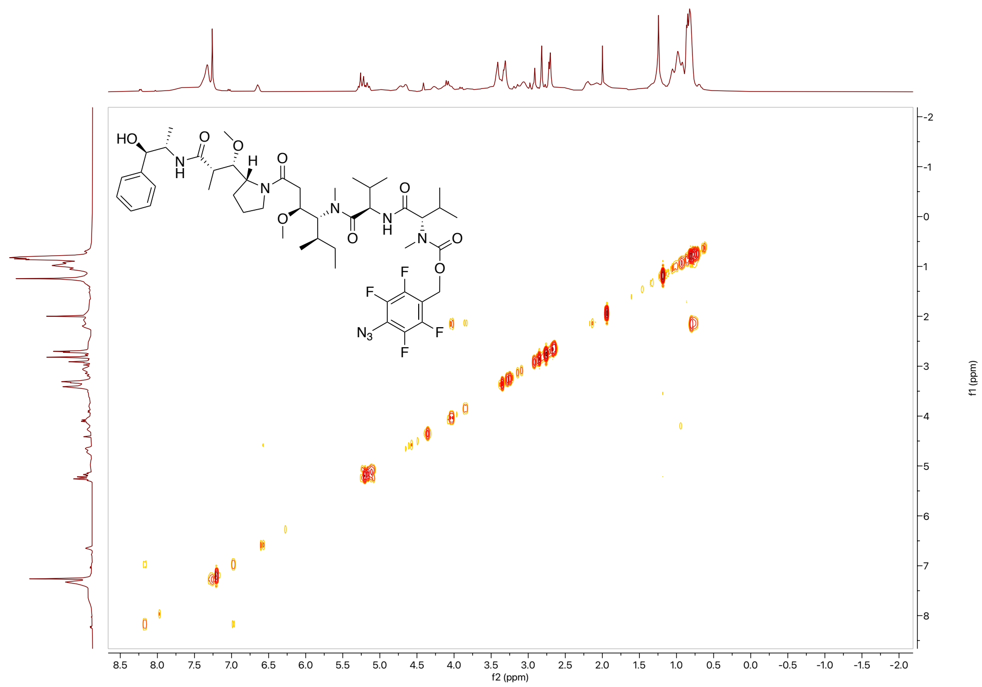


COSY NMR of pATFB-MMAE in $CDCl_3$.

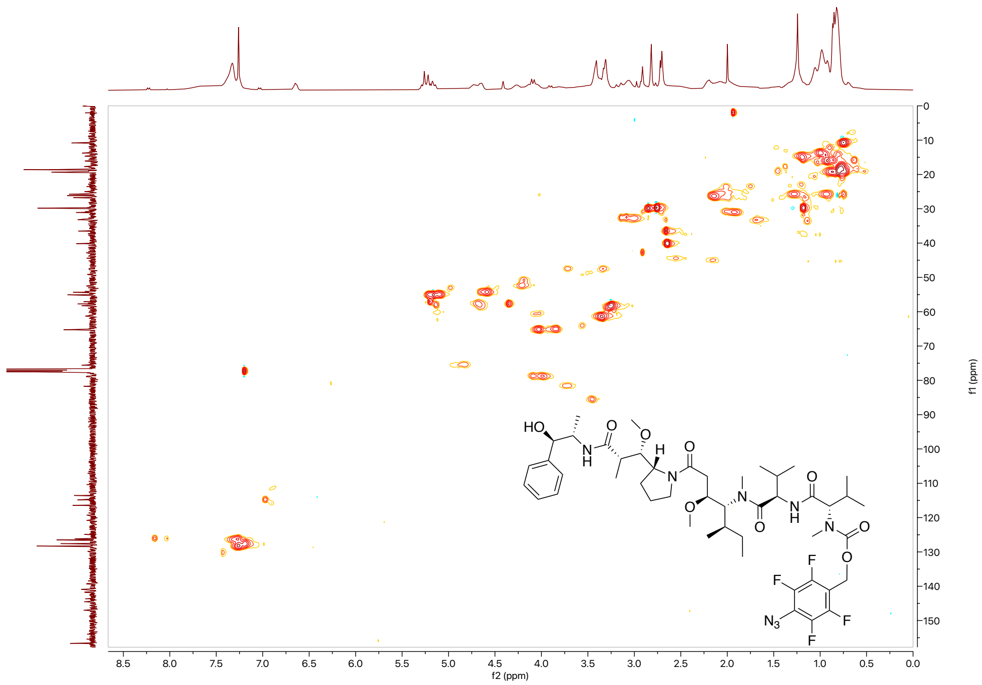


HSQC NMR of pATFB-MMAE in $CDCl_3$.

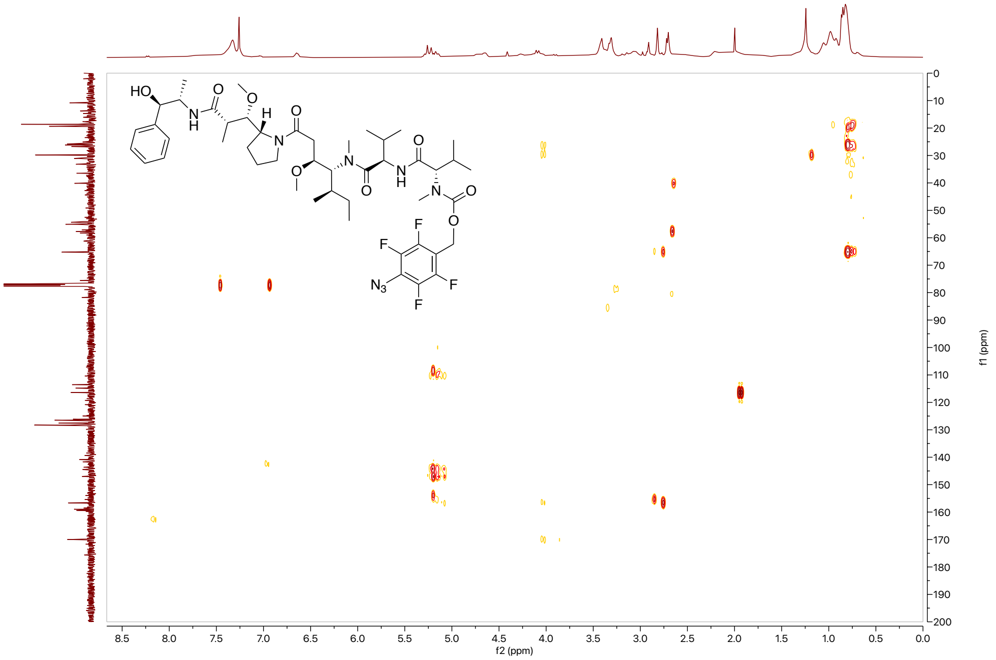


HMBC NMR of pATFB-MMAE in $CDCl_3$.

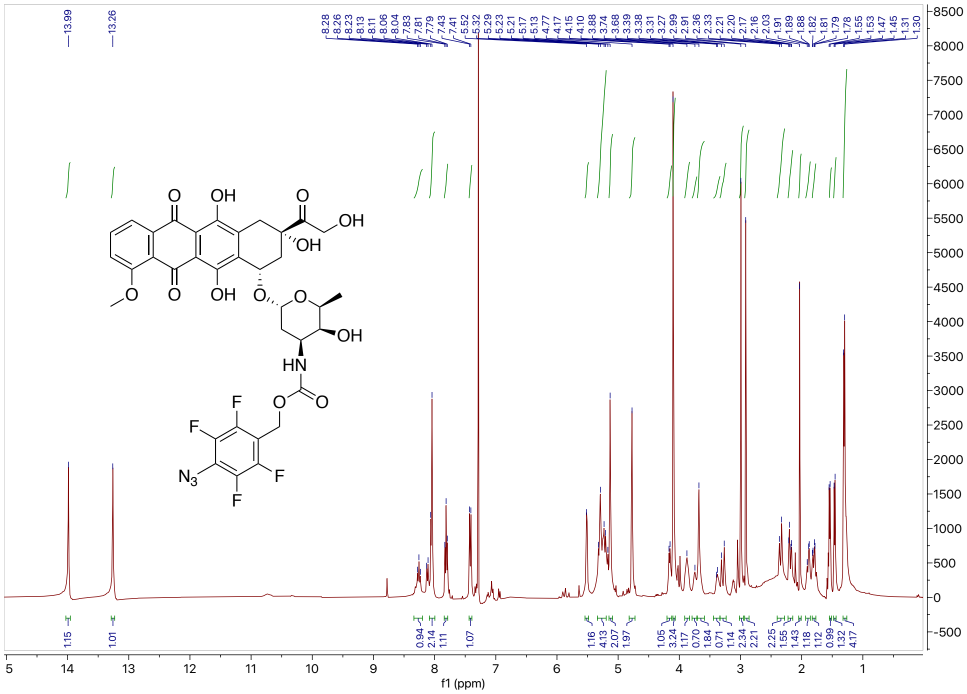


$^{1}$H NMR of pATFB-DOX in $CDCl_3$.

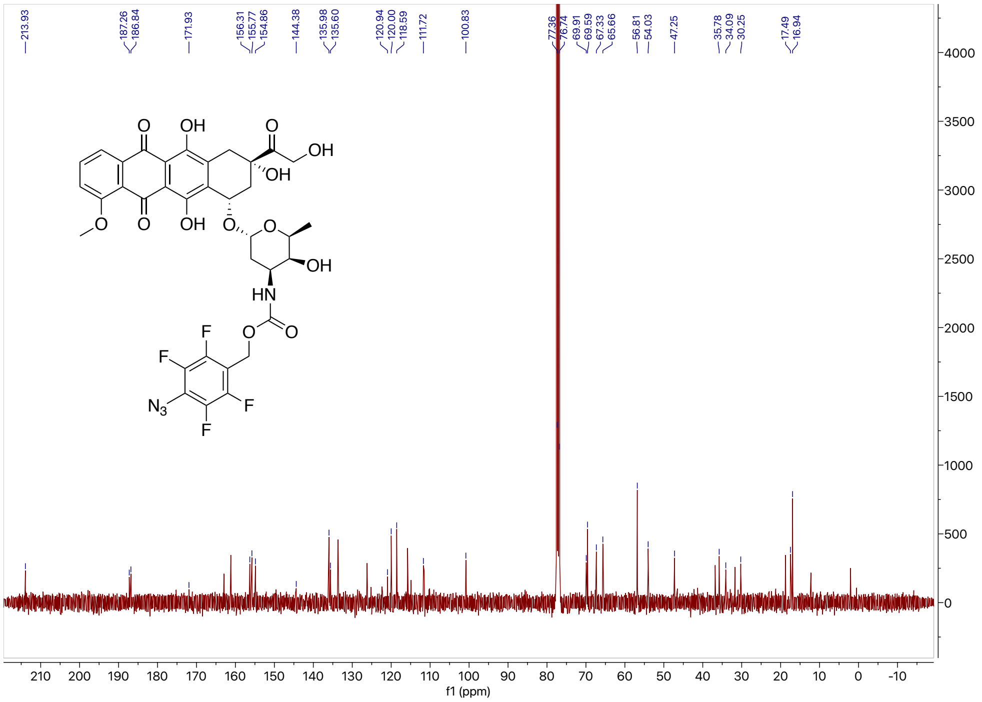


$^{13}$C NMR of pATFB-DOX in $CDCl_3$.

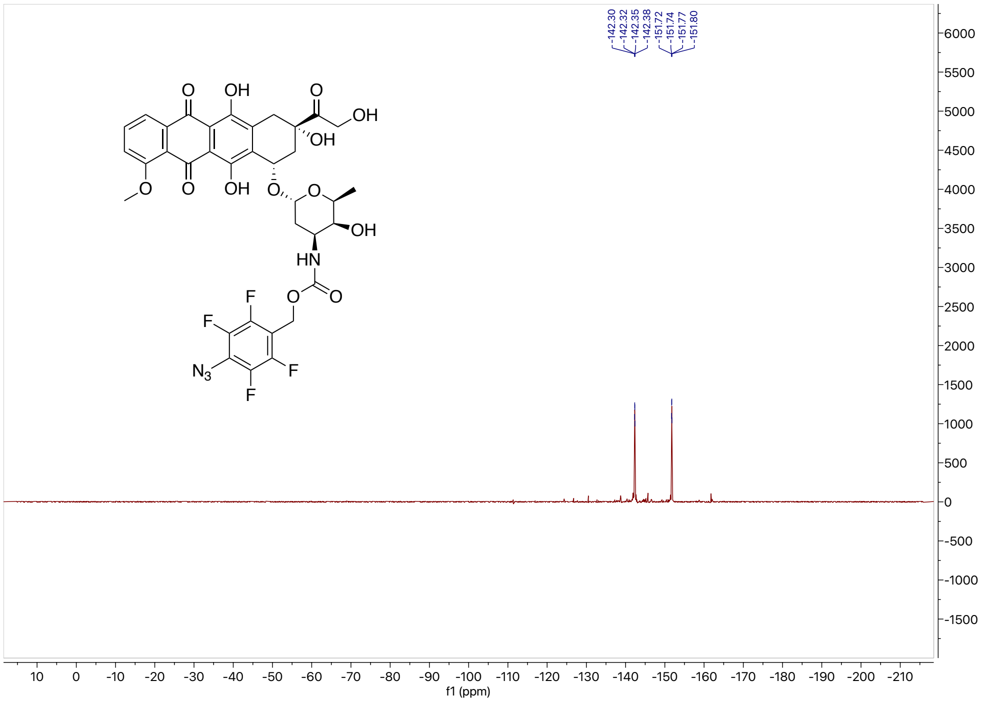


$^{19}$F NMR of pATFB-DOX in $CDCl_3$.

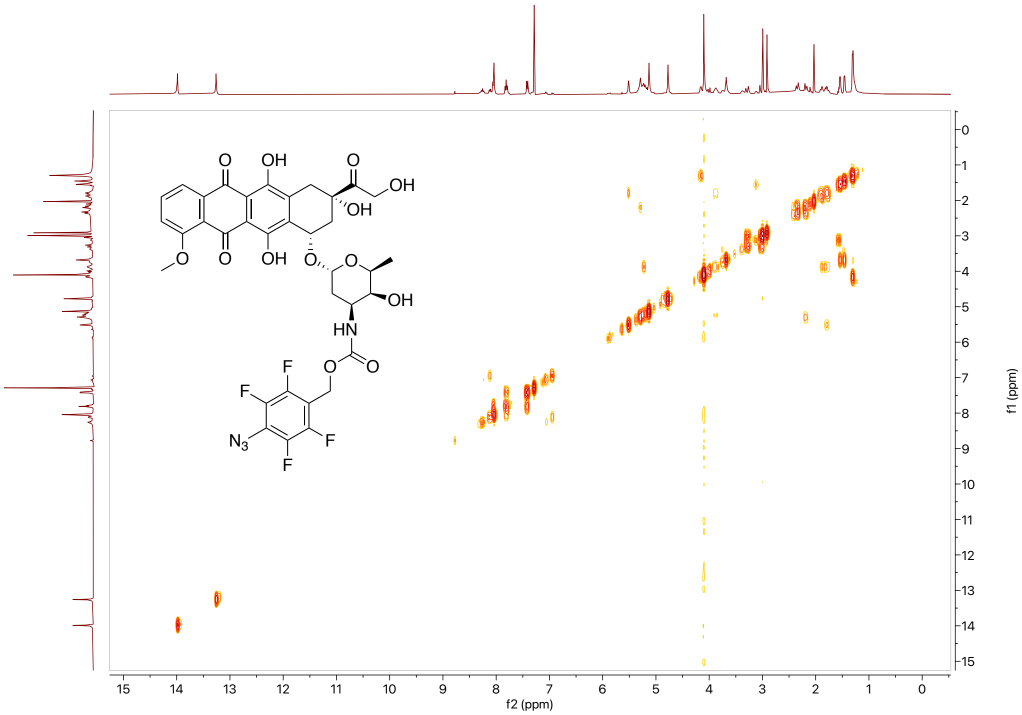


COSY NMR of pATFB-DOX in $CDCl_3$.

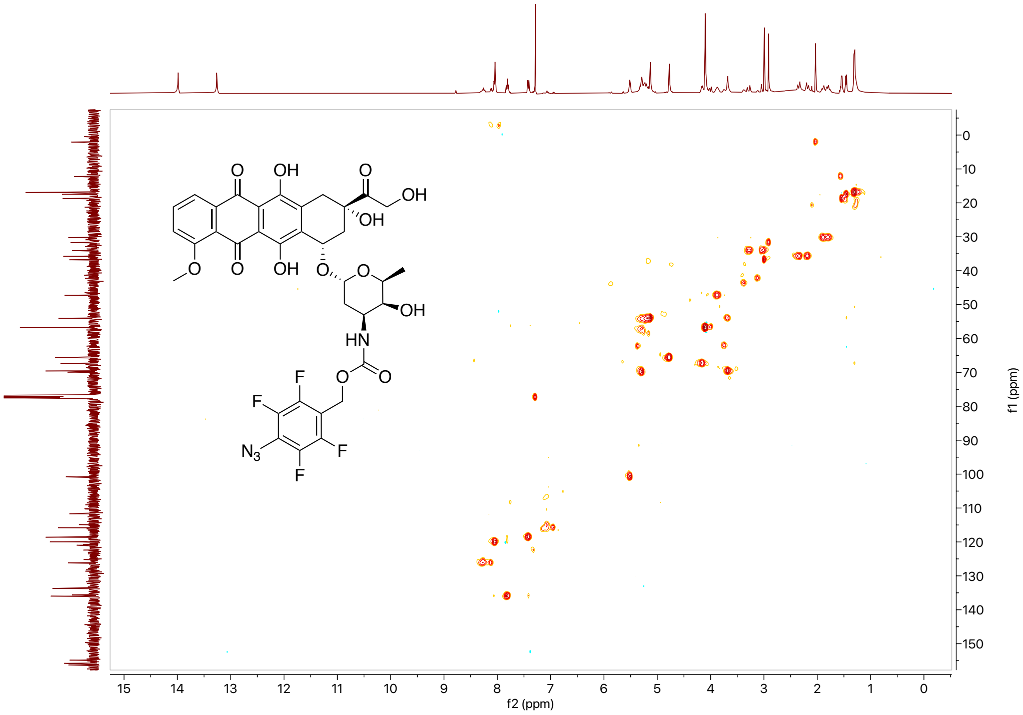


HSQC NMR of pATFB-DOX in $CDCl_3$.

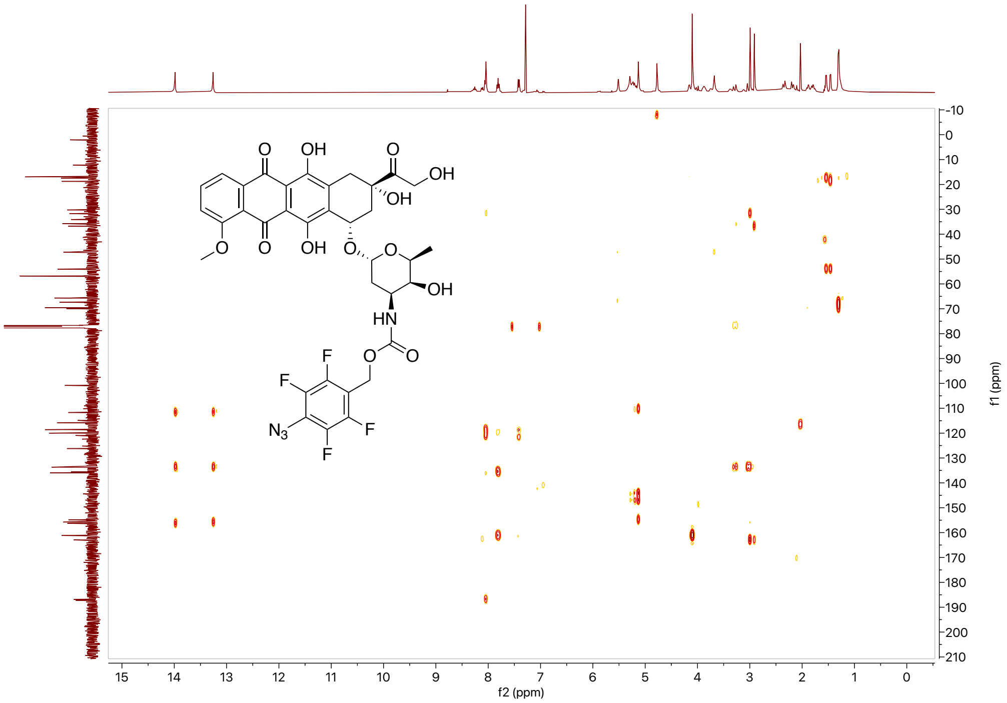

HMBC NMR of pATFB-DOX in $CDCl_3$.

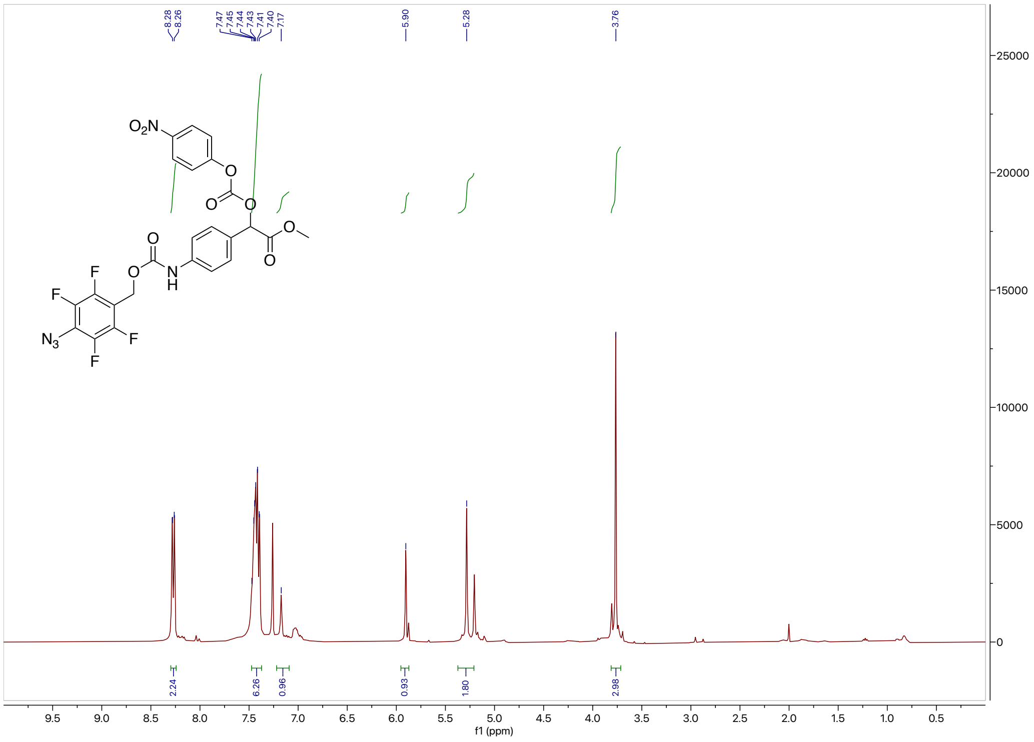

$^1H$ NMR of pATFB-SIL-PNP in $CDCl_3$.

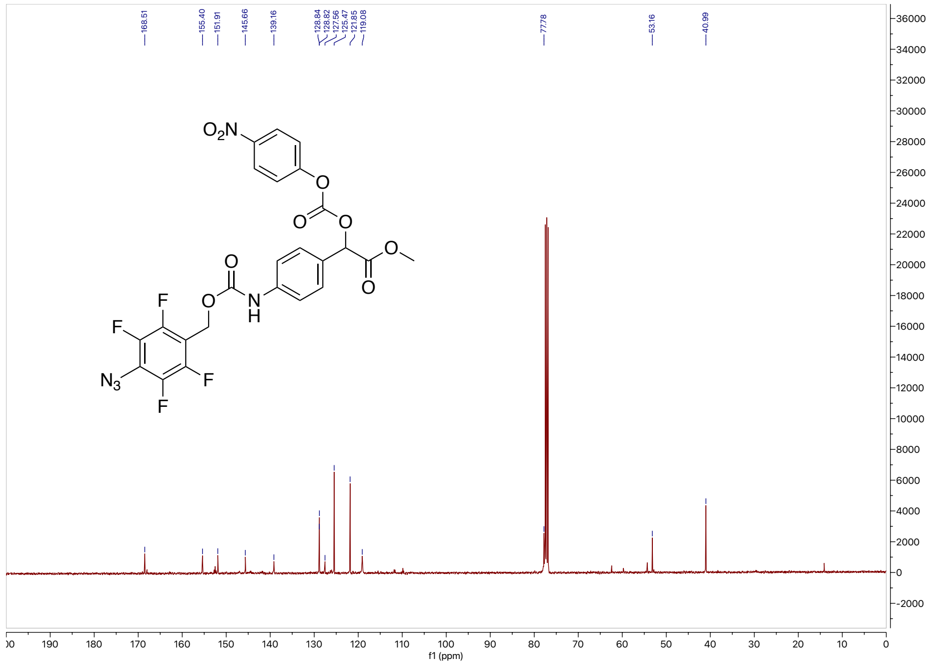


$^{13}C$ NMR of pATFB-SIL-PNP in $CDCl_3$.

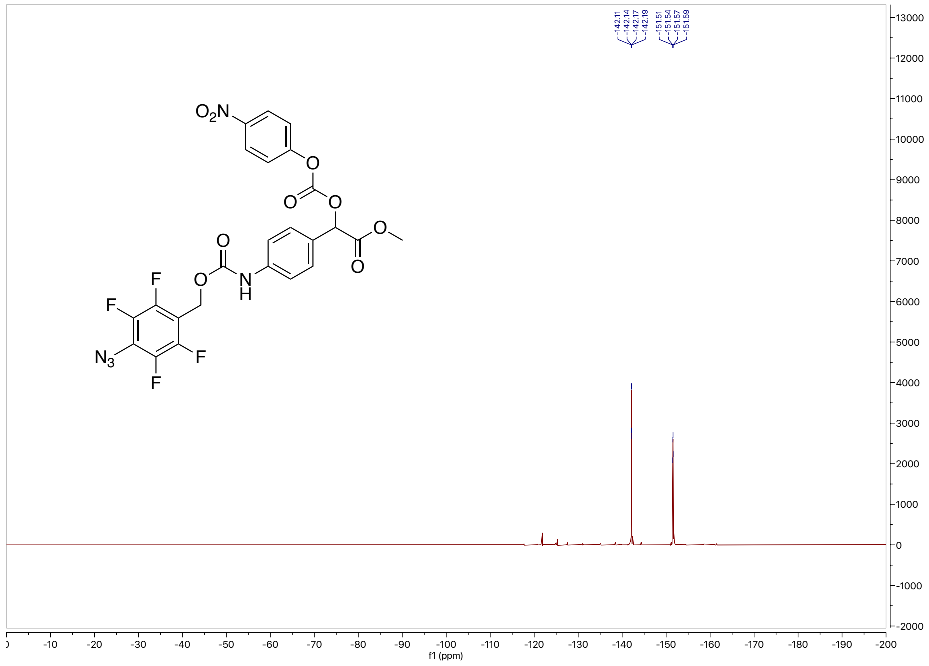


$^{19}F$ NMR of pATFB-SIL-PNP in $CDCl_3$.

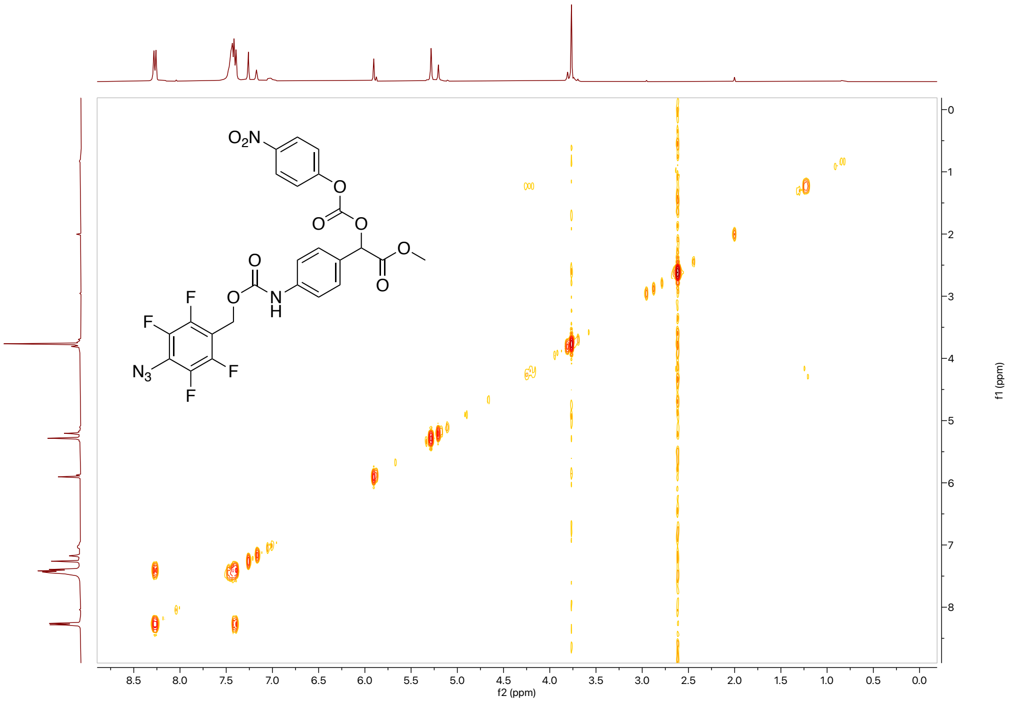


COSY NMR of pATFB-SIL-PNP in $CDCl_3$.

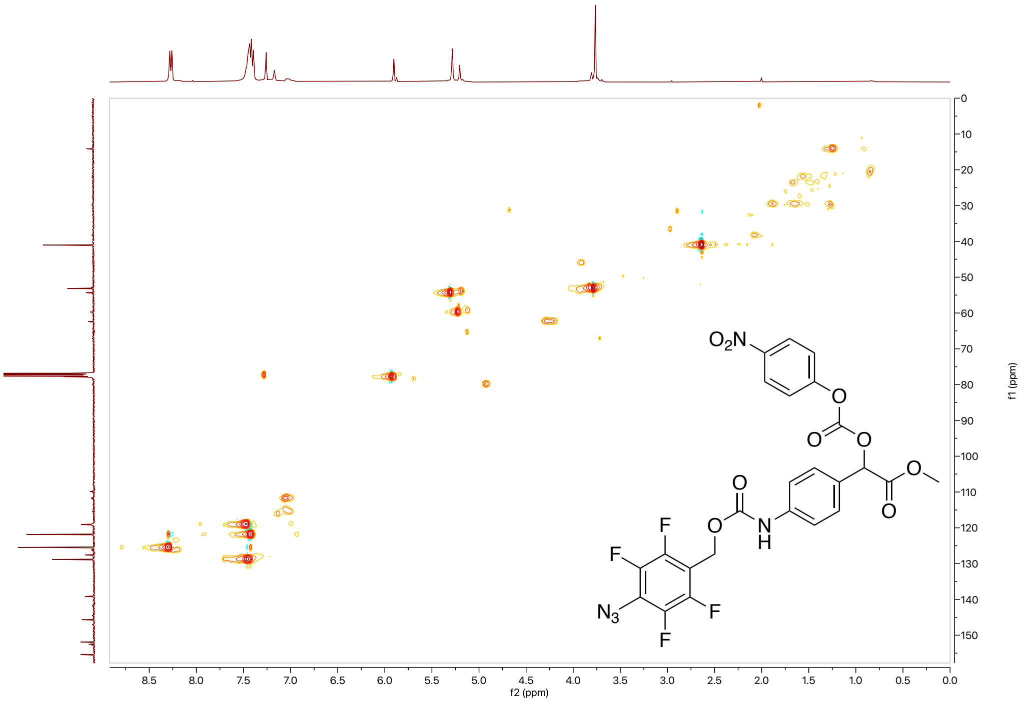


HSQC NMR of pATFB-SIL-PNP in $CDCl_3$.

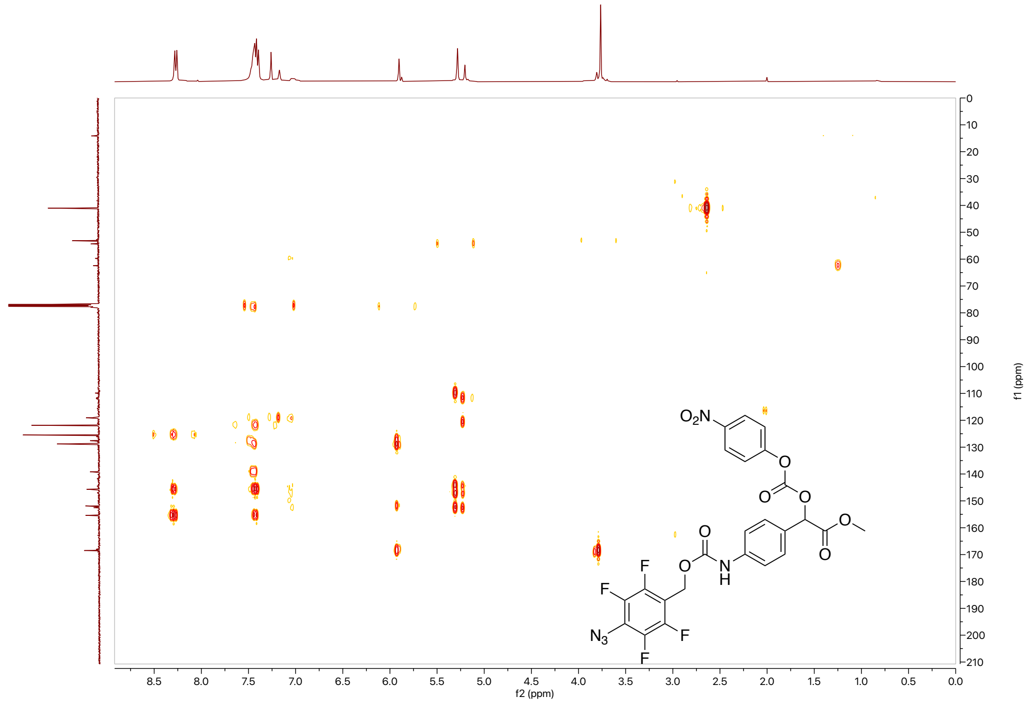

HMBC NMR of pATFB-SIL-PNP in $CDCl_3$.

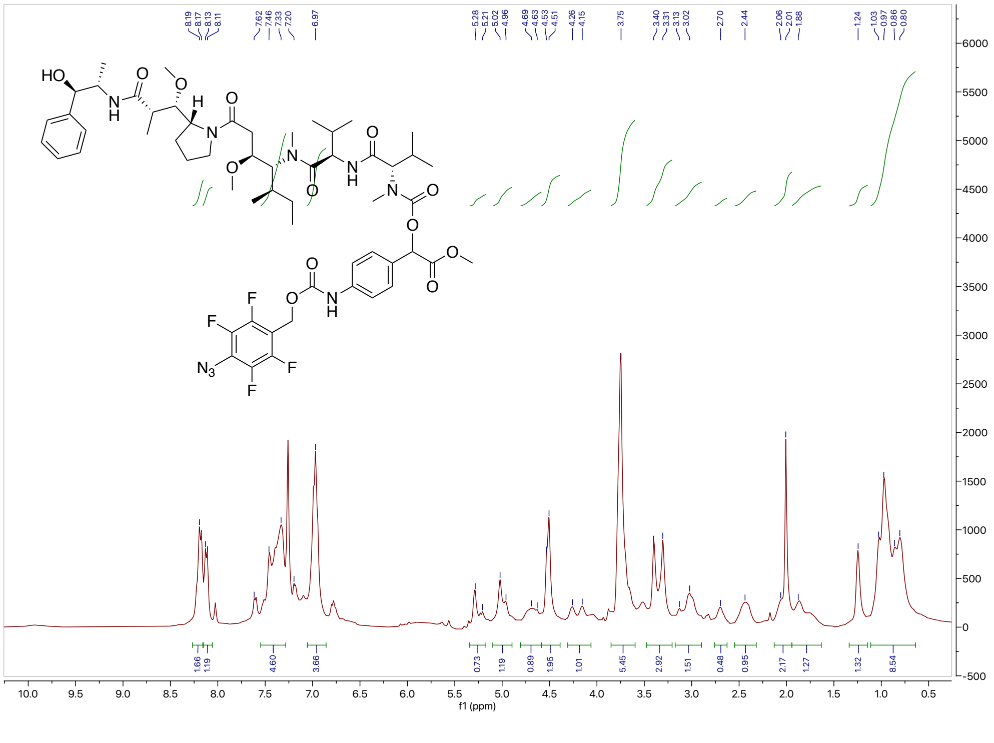

$^1H$ NMR of pATFB-SIL-MMAE in $CDCl_3$.

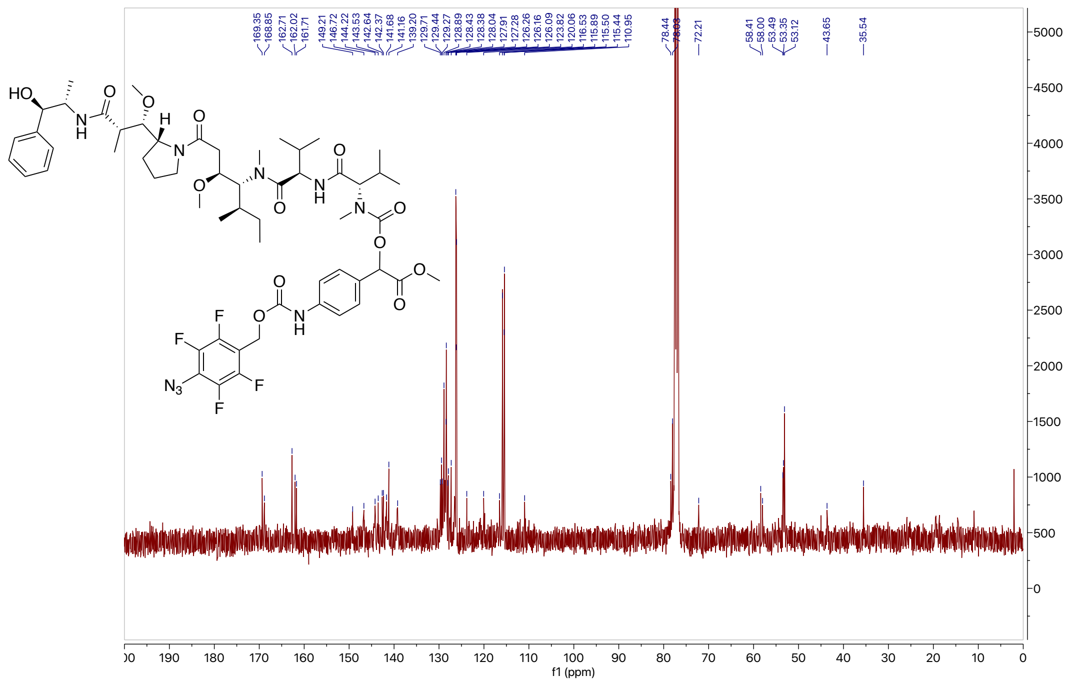

$^{13}C$ NMR of pATFB-SIL-MMAE in $CDCl_3$.

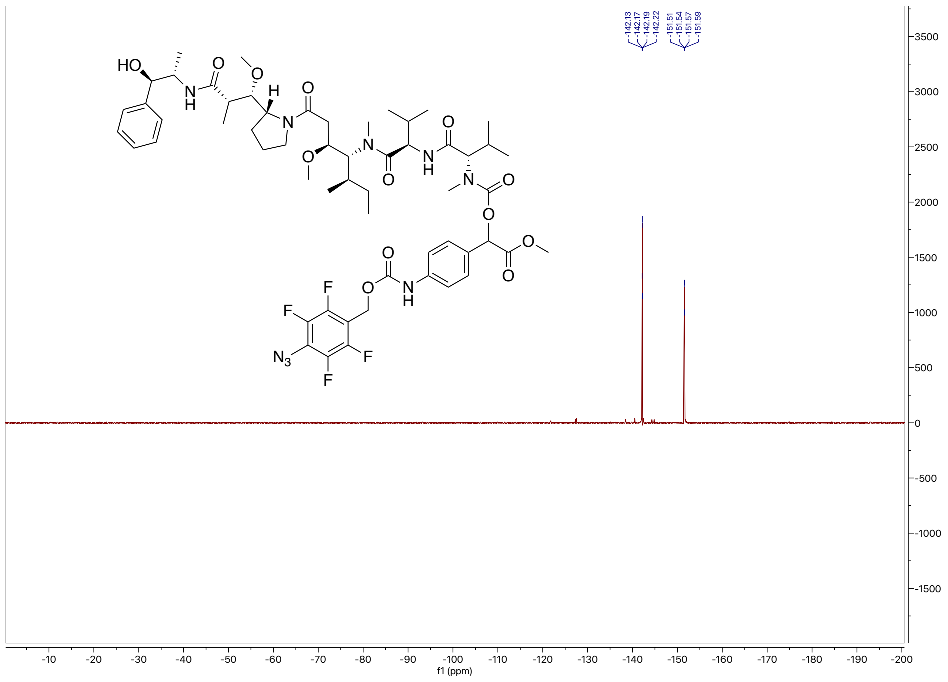

$^{19}F$ NMR of pATFB-SIL-MMAE in $CDCl_3$.

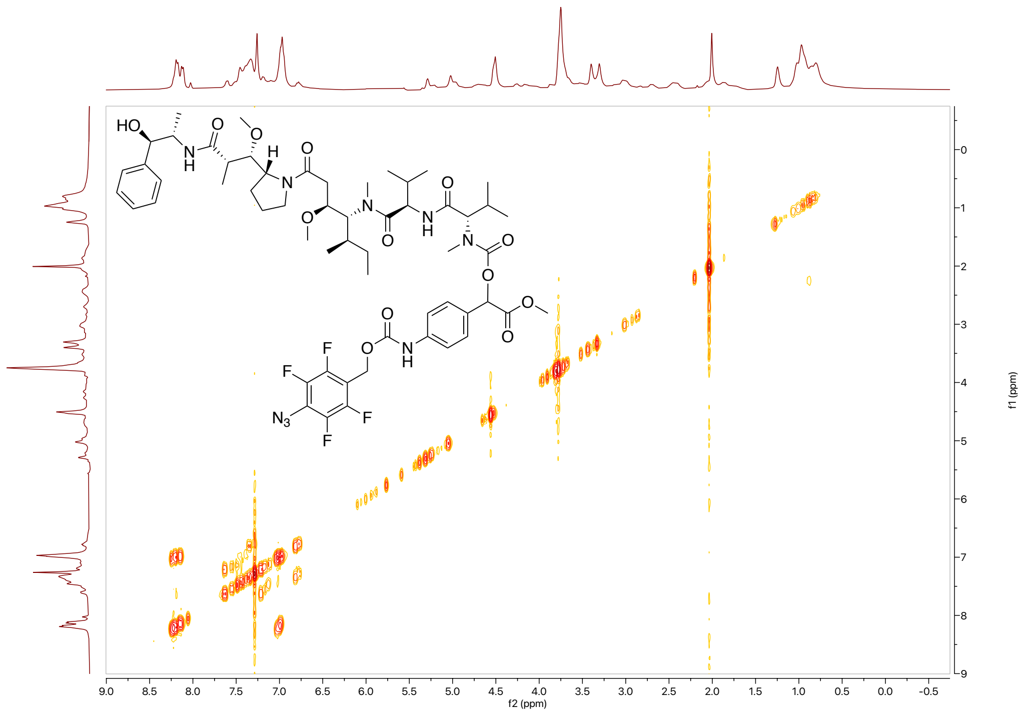


COSY NMR of pATFB-SIL-MMAE in $CDCl_3$.

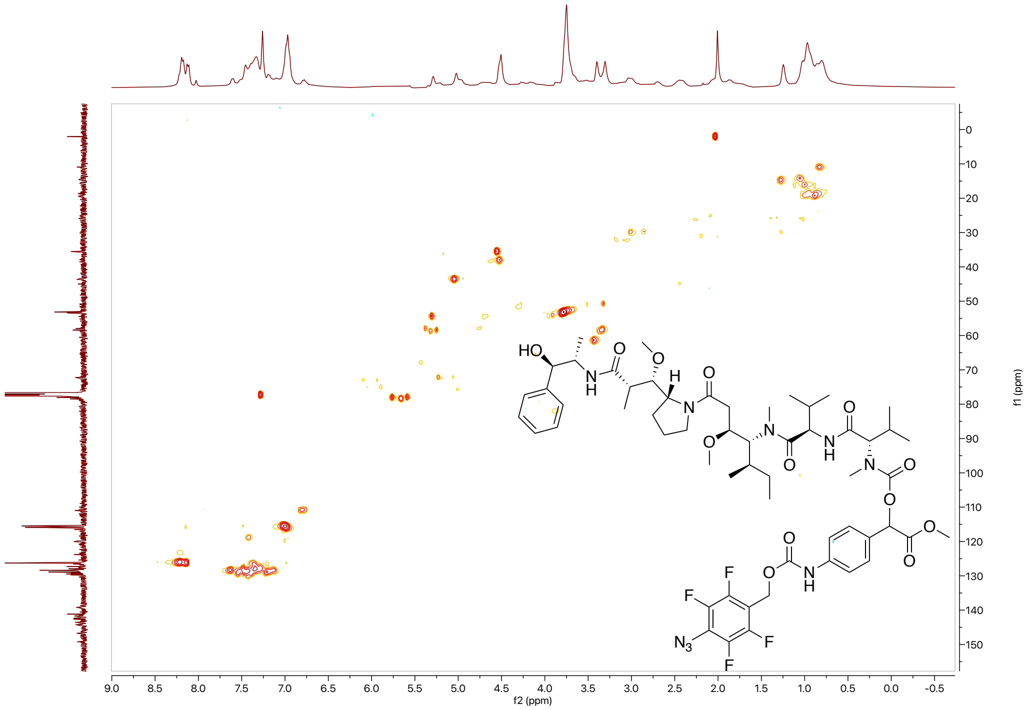


HSQC NMR of pATFB-SIL-MMAE in $CDCl_3$.

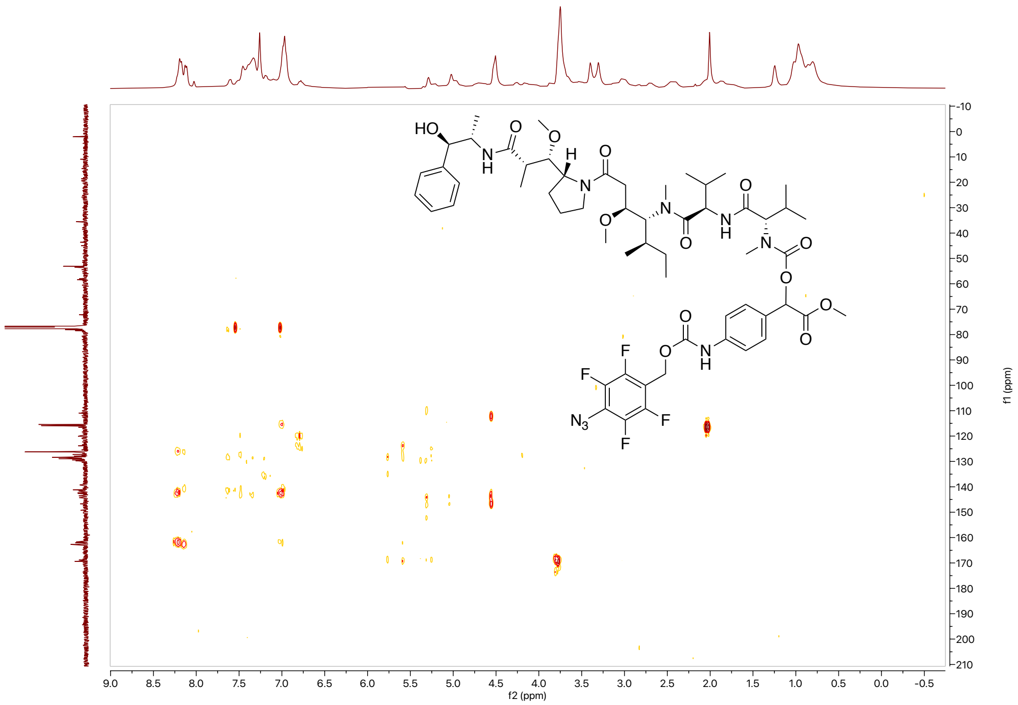

HMBC NMR of pATFB-SIL-MMAE in $CDCl_3$.

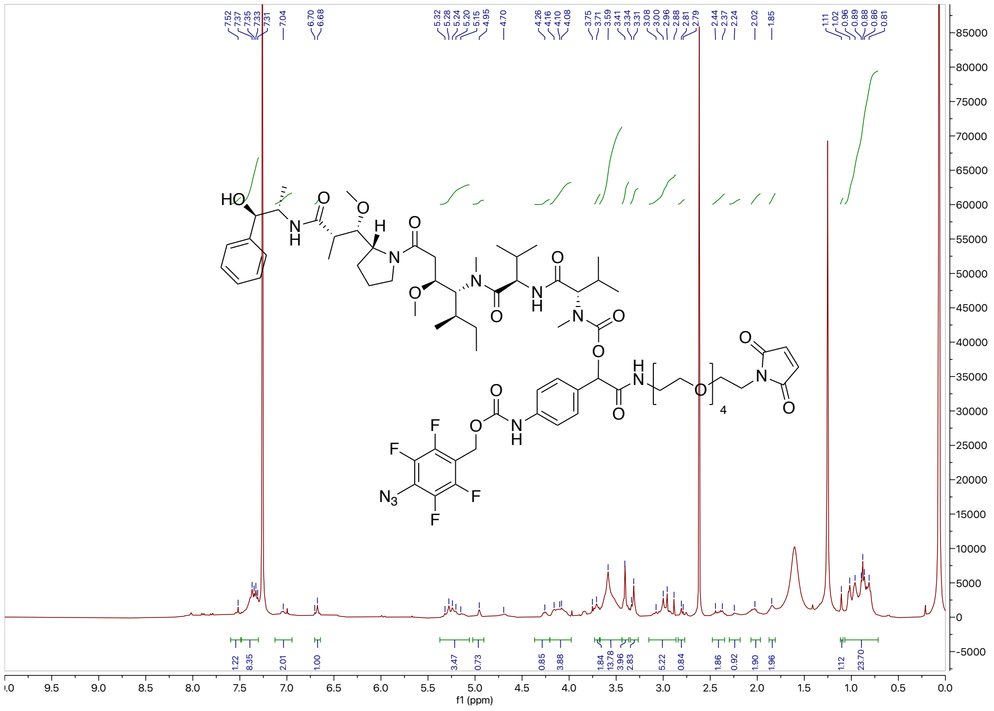

$^{1}$H NMR of pATFB-SIL-Mal-MMAE in $CDCl_3$.

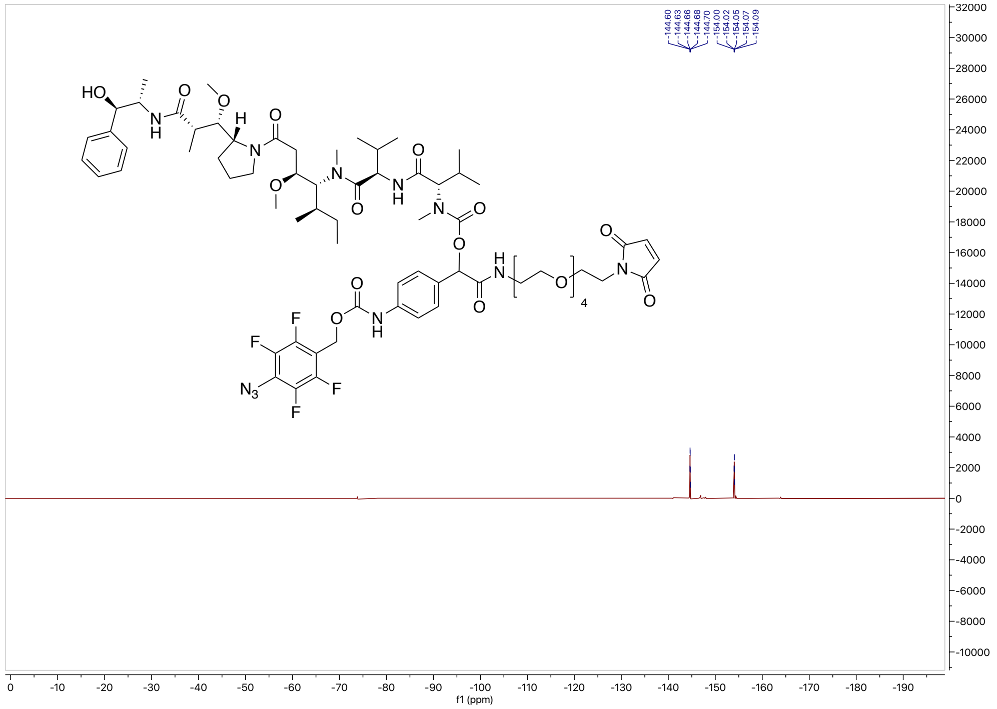


$^{19}$F NMR of pATFB-SIL-Mal-MMAE in $CDCl_3$.

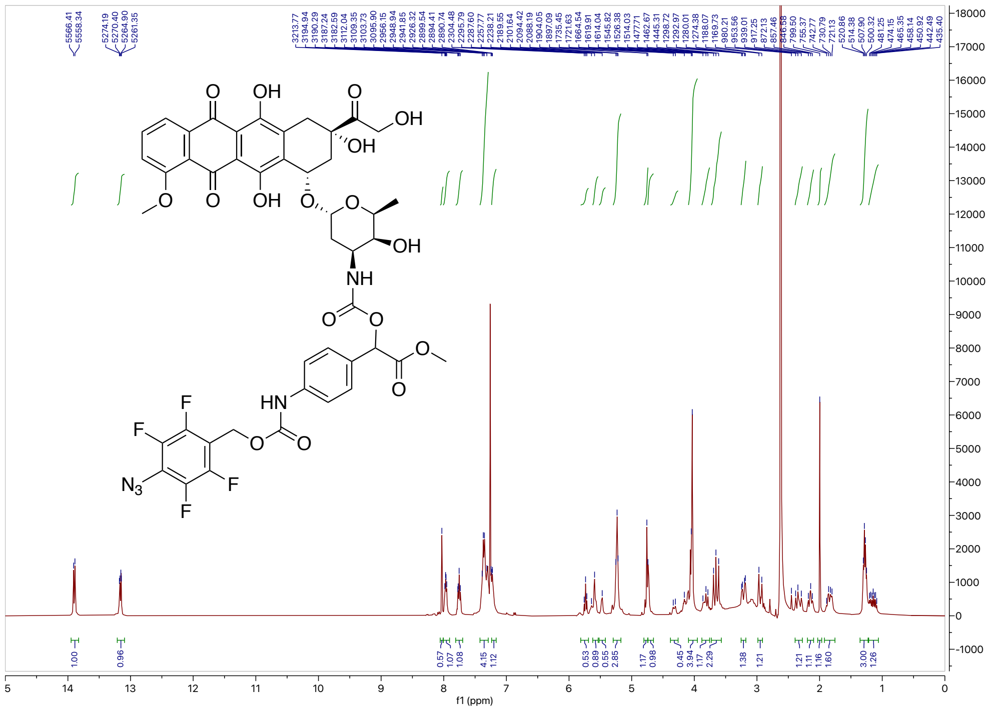


$^{1}$H NMR of pATFB-SIL-DOX in $CDCl_3$.

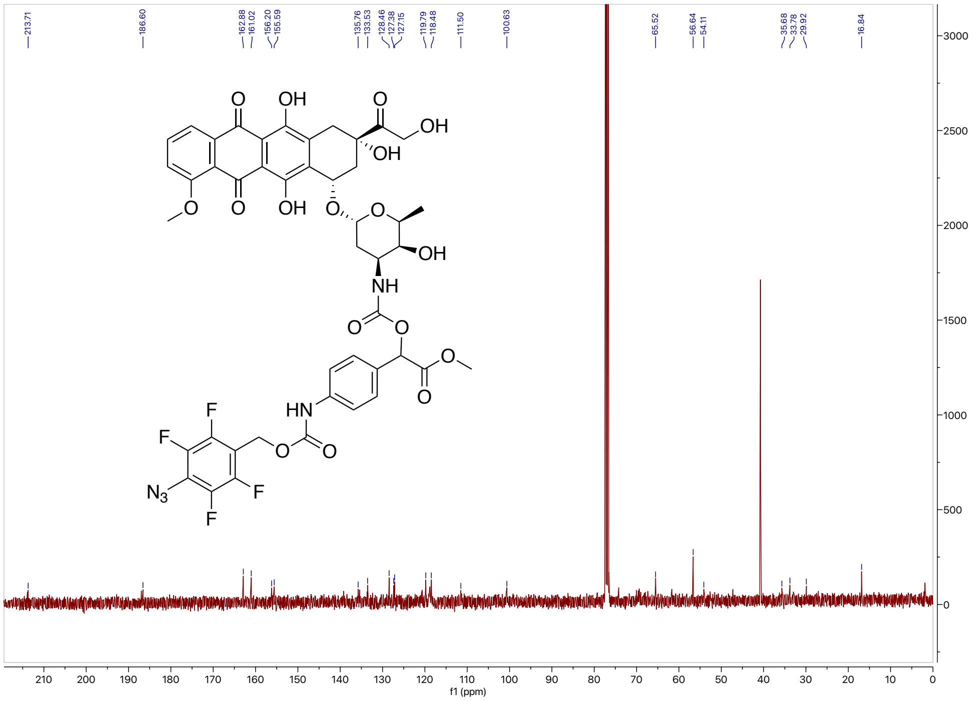


$^{13}C$ NMR of pATFB-SIL-DOX in $CDCl_3$.

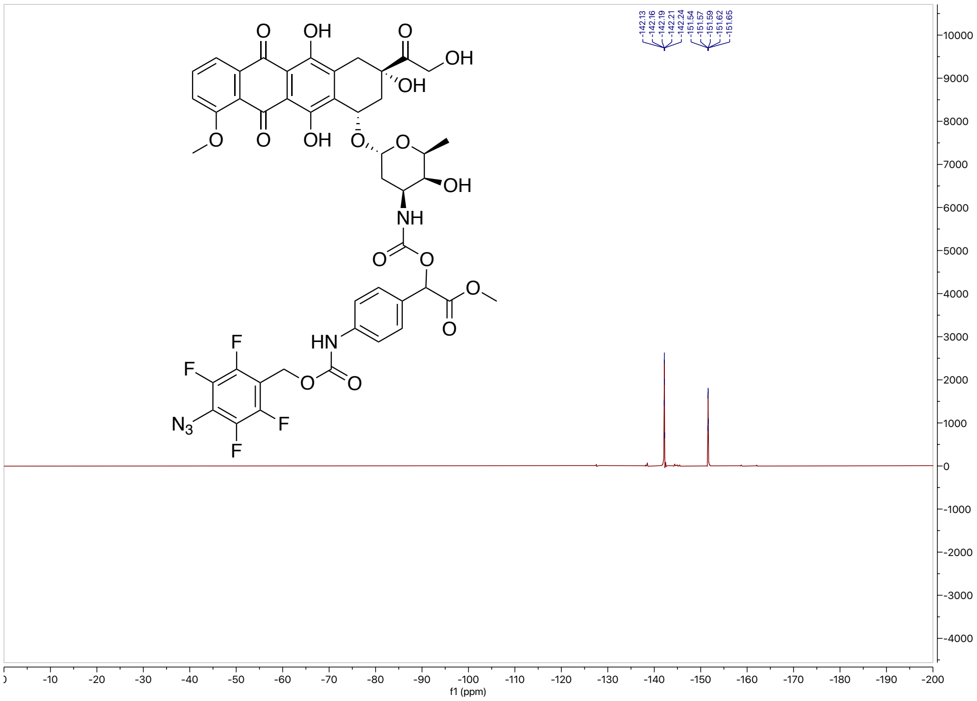


$^{19}F$ NMR of pATFB-SIL-DOX in $CDCl_3$.

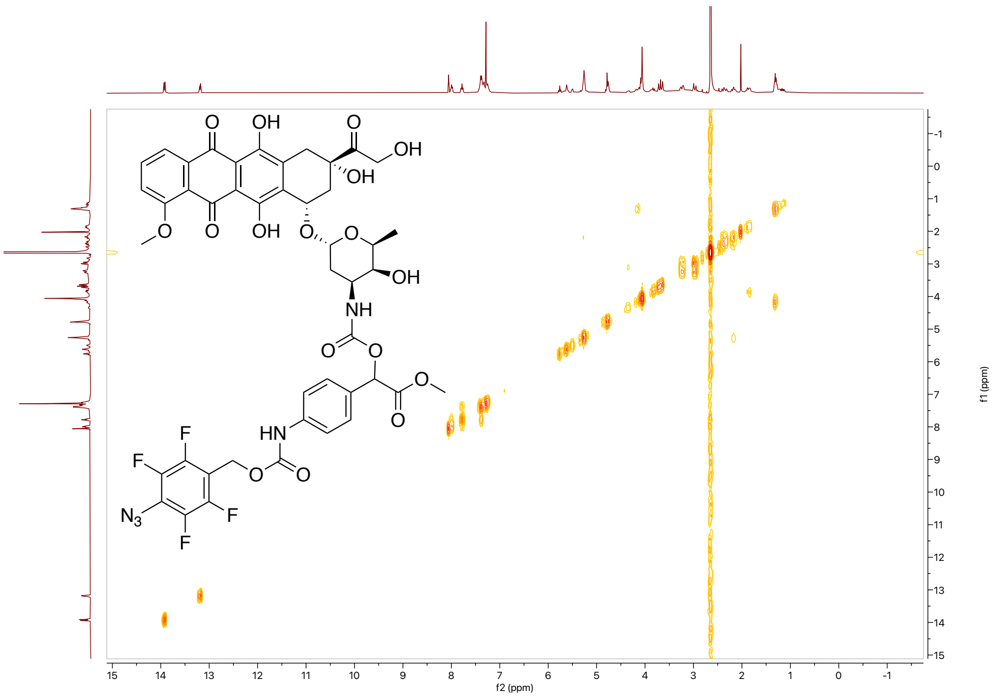


COSY NMR of pATFB-SIL-DOX in $CDCl_3$.

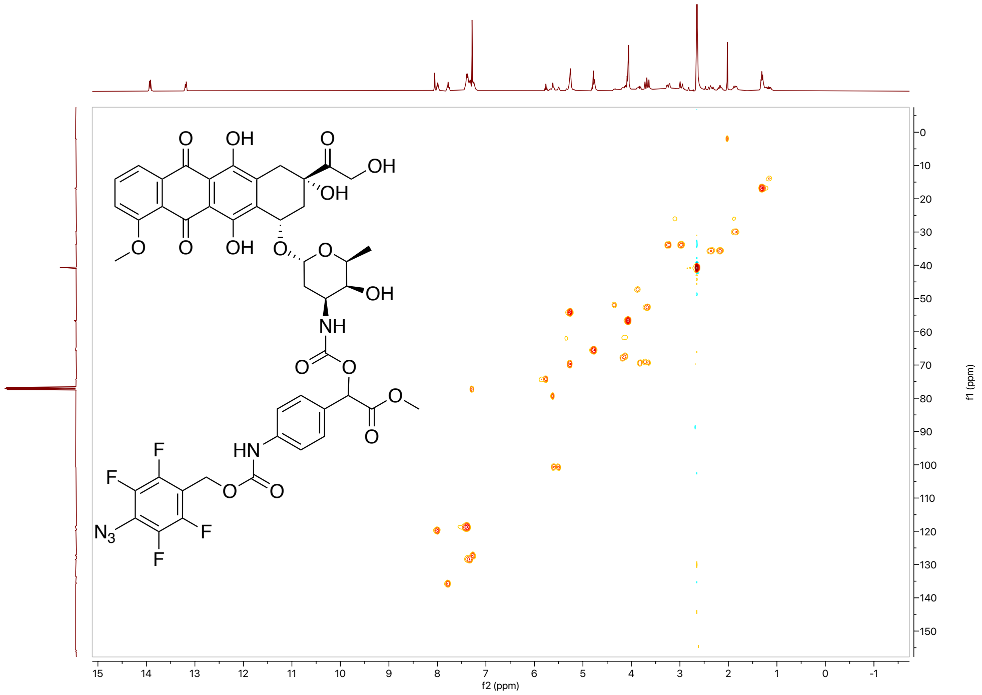


HSQC NMR of pATFB-SIL-DOX in $CDCl_3$.

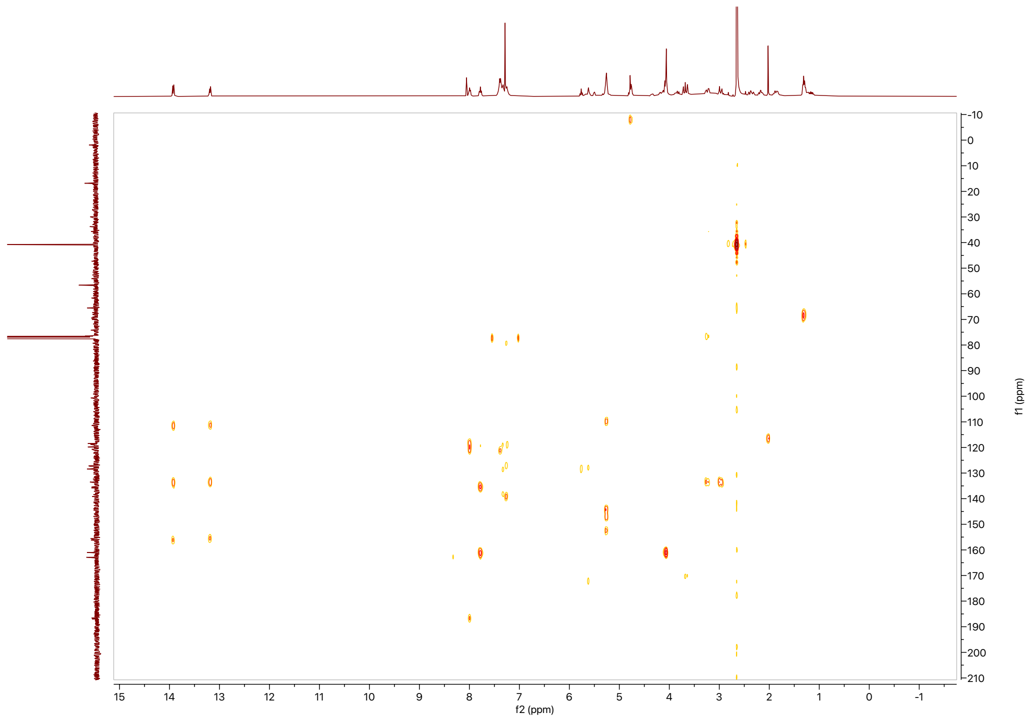


HMBC NMR of pATFB-SIL-DOX in $CDCl_3$.

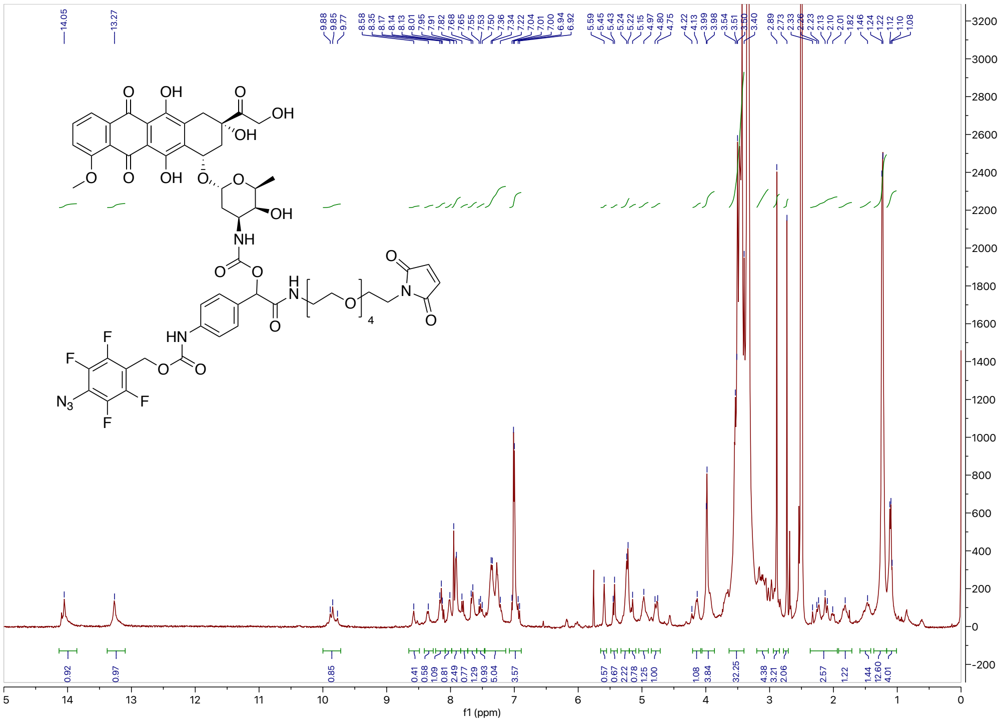


$^1H$ NMR of pATFB-SIL-Mal-DOX in DMSO-$d_6$.

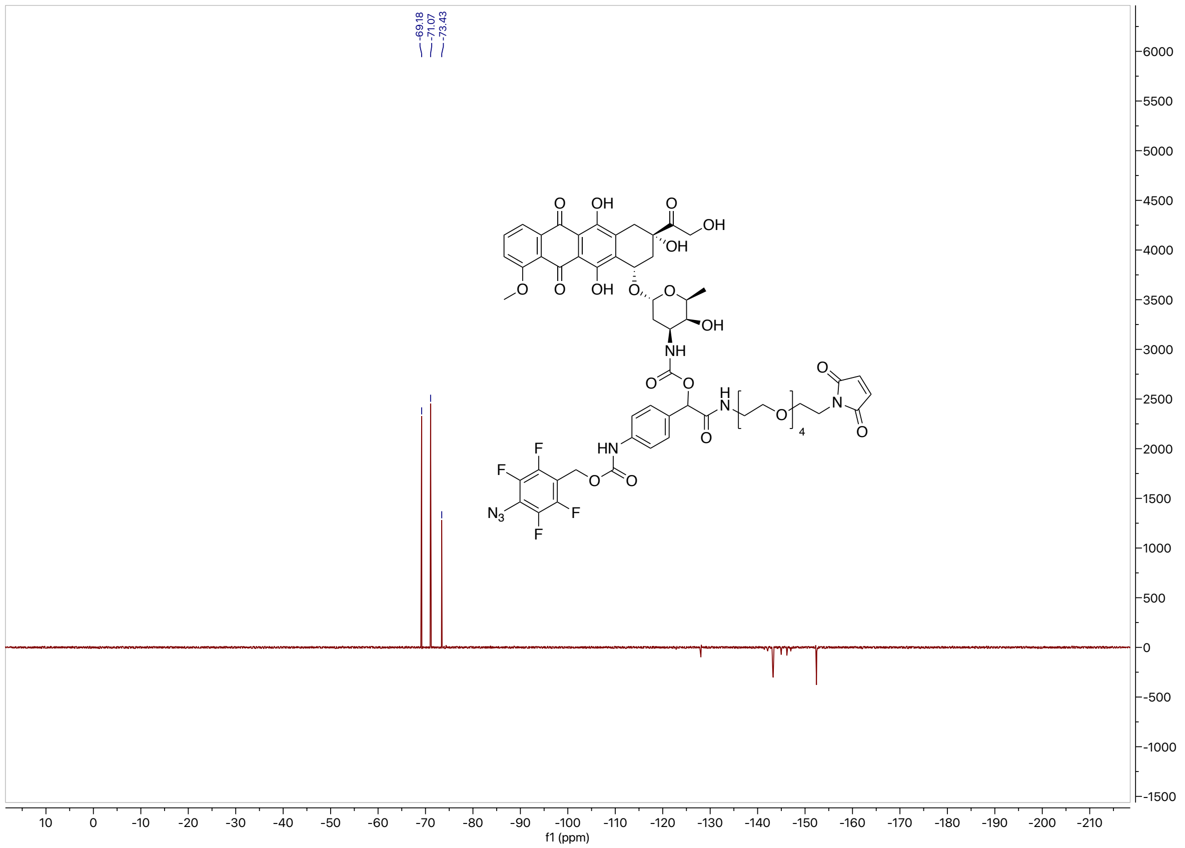


$^{19}F$ NMR of pATFB-SIL-Mal-DOX in DMSO-$d_6$.

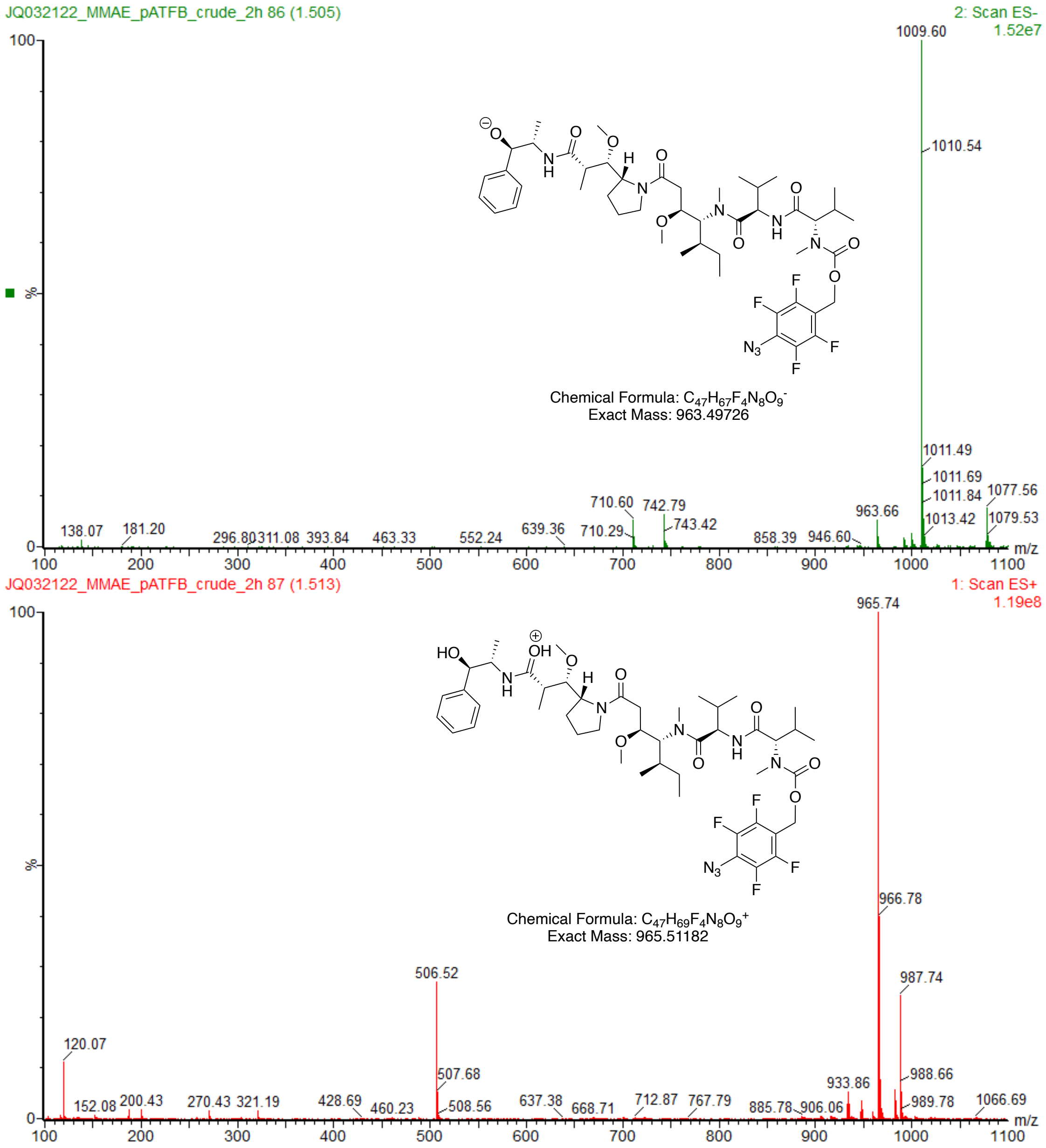


ESI spectra of pATFB-MMAE.

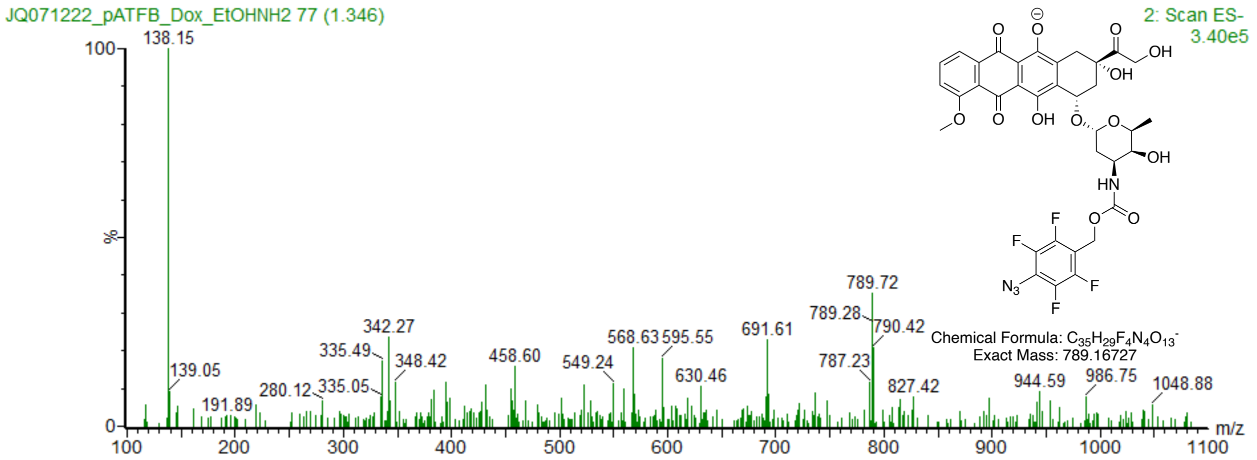


-ESI spectra of pATFB-DOX.

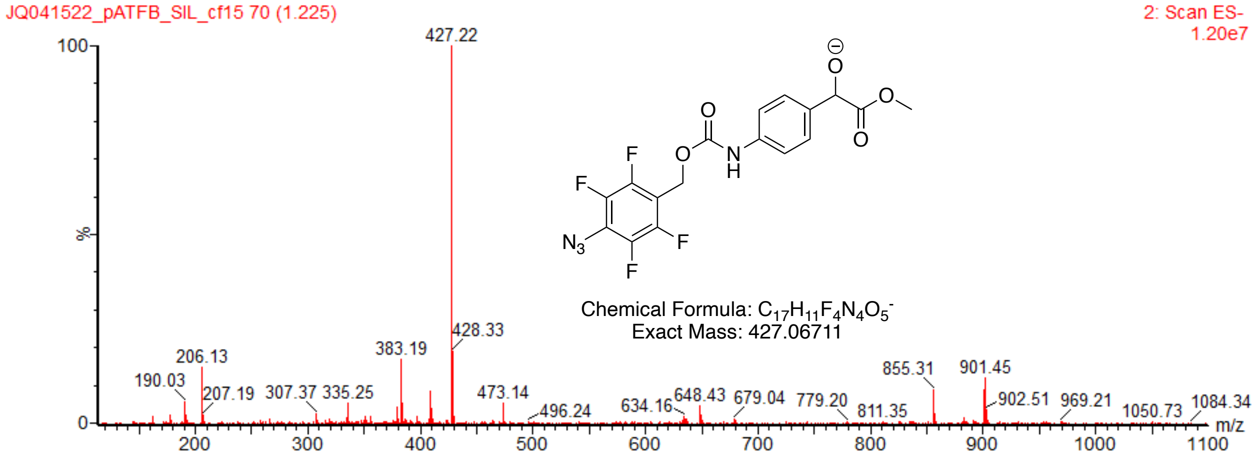


-ESI spectra of pATFB-SIL.

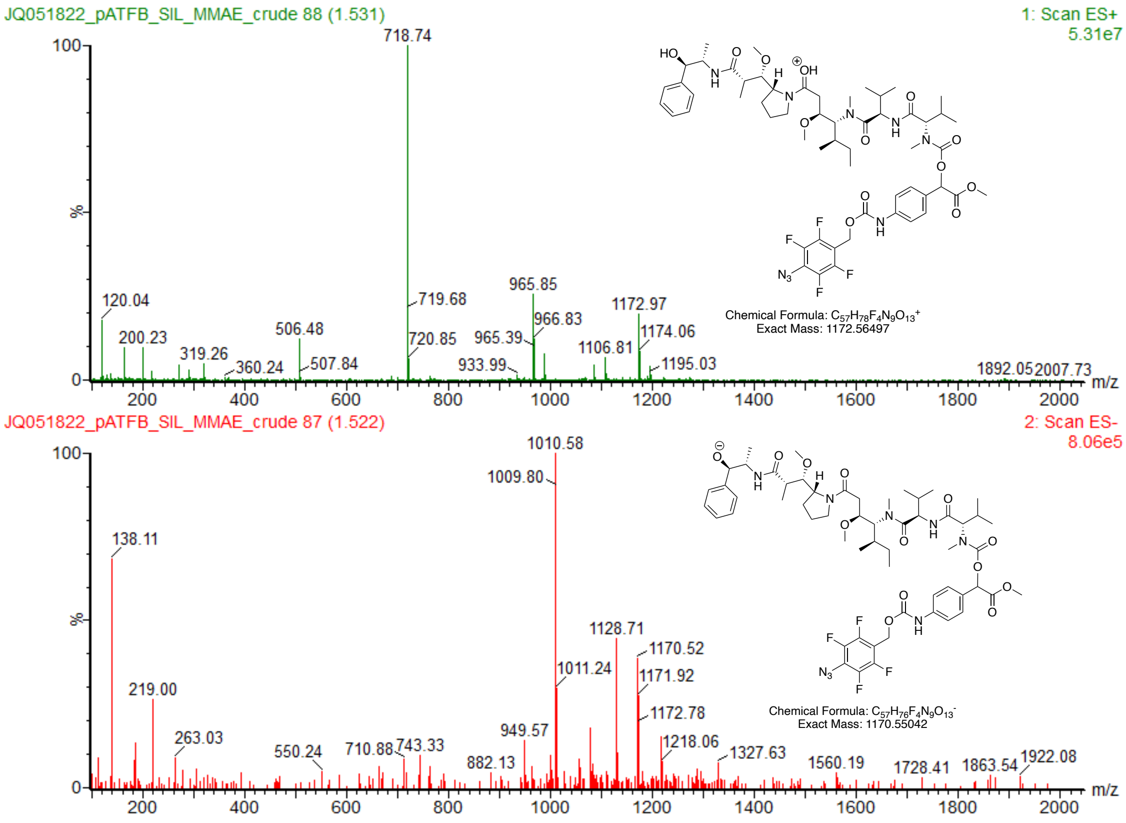


ESI spectra of pATFB-SIL-MMAE.

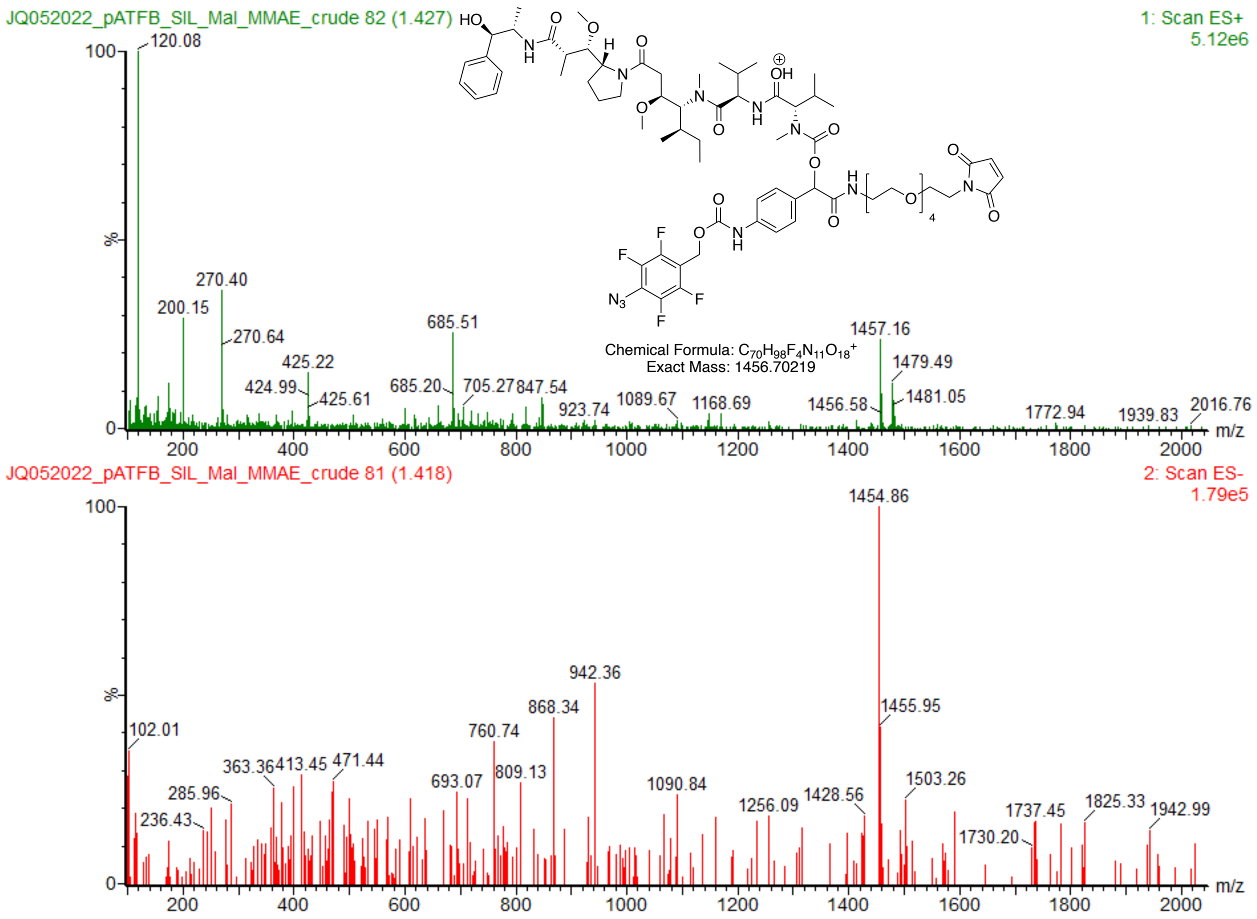


ESI spectra of pATFB-SIL-Mal-MMAE.

# 5. References


1. Perl, J.; Shin; J.; Schumann, J.; Faddegon, B.; Paganetti, H. TOPAS: an innovative proton Monte Carlo platform for research and clinical applications. *Med Phys*. **2012**, 39(11), 6818-6837.
2. Testa, M.; Schümann, J.; Lu, H.M. et al. Experimental validation of the TOPAS Monte Carlo system for passive scattering proton therapy. *Med Phys*. **2013**, 40(12) 121719.
3. Franken, N.A.; Rodermond, H.M.; Stap, J.; Haveman, J.; van Bree, C. Clonogenic assay of cells in vitro. *Nat Protoc*. **2006**, 1(5), 2315-2319.
4. Vanden Borre, P.; McFadden, D.G.; Gunda, V. et al. The Next Generation of Orthotopic Thyroid Cancer Models: Immunocompetent Orthotopic Mouse Models of BRAFV600E-Positive Papillary and Anaplastic Thyroid Carcinoma. *Thyroid*. **2014**, 24(4), 75-714.
5. Kang, M.; Quintana, J.; Hu, H. et al. Sustained and Localized Drug Depot Release using Radiation-Activated Scintillating Nanoparticles. *Adv Mater*. **2024**, doi:10.1002/adma.202312326.
6. Hu, H.; Ng, T.S.C.; Kang, M. et al. Thyroid Cancers Exhibit Oncogene-Enhanced Macropinocytosis that Is Restrained by IGF1R and Promote Albumin–Drug Conjugate Response. *Clin Cancer Res*. **2023**, 29(17), OF1-OF14.
7. Ng, T.S.C.; Hu, H.; Kronister, S. et al. Overcoming differential tumor penetration of BRAF inhibitors using computationally guided combination therapy. *Sci Adv*. **2022**, 8(17) eabl6339.
8. Kim, H.Y.; Li, R.; Ng, T.S.C. et al. Quantitative Imaging of Tumor-Associated Macrophages and Their Response to Therapy Using (64)Cu-Labeled Macrin. *ACS Nano*. **2018**, 12(12), 12015-12029.
9. Geng, J.; Zhang, Y.; Gao, Q. et al. Switching on prodrugs using radiotherapy. *Nat Chem*. **2021**, 13, 805-810.
10. Matikonda, S.S.; Fairhall, J.M.; Fiedler, F. et al. Mechanistic Evaluation of Bioorthogonal Decaging with trans-Cyclooctene: The Effect of Fluorine Substituents on Aryl Azide Reactivity and Decaging from the 1,2,3-Triazoline. *Bioconjugate Chem*. **2018**, 29(2) 324-334.
11. Zhang, L.; Bhatnagar, S.; Deschenes, E.; Thurber, G.M. Mechanistic and quantitative insight into cell surface targeted molecular imaging agent design. *Sci Rep*. **2016**, 6, 25424.
12. Hingorani, D. V.; Allevato, M. M.; Camargo, M. F.; Lesperance, J.; Quraishi, M. A.; Aguilera, J.; Franiak-Pietryga, I.; Scanderbeg, D. J.; Wang, Z.; Molinolo, A. A. et al. Monomethyl auristatin antibody and peptide drug conjugates for trimodal cancer chemo-radio-immunotherapy Nat. Commun. **2022**, 13, 3869.
13. Quintana, J. M.; Arboleda, D.; Hu, H.; Scott, E.; Luthria, G.; Pai, S.; Parangi, S.; Weissleder, R.; Miller, M. A. Radiation Cleaved Drug-Conjugate Linkers Enable Local Payload Release Bioconjugate Chem. **2022**, 33, 1474-1484.
14. Miller, M. A.; Mikula, H.; Luthria, G.; Li, R.; Kronister, S.; Prytyskach, M.; Kohler, R. H.; Mitchison, T.; Weissleder, R. Modular Nanoparticulate Prodrug Design Enables Efficient Treatment of Solid Tumors Using Bioorthogonal Activation ACS nano. **2018**, 12, 12814-12826.

15. Kratschmer, C.; Levy, M. Targeted Delivery of Auristatin-Modified Toxins to Pancreatic Cancer Using Aptamers. Mol. Ther.—Nucleic Acids. **2018**, 10, 227-236.
